# Author Correction: Engineered bioorthogonal POLY-PROTAC nanoparticles for tumour-specific protein degradation and precise cancer therapy

**DOI:** 10.1038/s41467-022-32722-1

**Published:** 2022-08-25

**Authors:** Jing Gao, Bo Hou, Qiwen Zhu, Lei Yang, Xingyu Jiang, Zhifeng Zou, Xutong Li, Tianfeng Xu, Mingyue Zheng, Yi-Hung Chen, Zhiai Xu, Huixiong Xu, Haijun Yu

**Affiliations:** 1 Department of Medical Ultrasound and Center of Minimally Invasive Treatment for Tumor, Shanghai Tenth People’s Hospital, Ultrasound Research and Education Institute, School of Medicine, Tongji University, Shanghai, 200072 China; 2 State Key Laboratory of Drug Research & Center of Pharmaceutics, Shanghai Institute of Materia Medica, Chinese Academy of Sciences, Shanghai, 201203 China; 3 School of Chemistry and Molecular Engineering, East China Normal University, Shanghai, 200241 China; 4 School of Chinese Materia Medica, Nanjing University of Chinese Medicine, Nanjing, 210023 China; 5 Institute for Advanced Studies (IAS), Wuhan University, Wuhan, 430072 China; 6 Department of Ultrasound, Zhongshan Hospital, Institute of Ultrasound in Medicine and Engineering, Fudan University, 200032 Shanghai, China

**Keywords:** Breast cancer, Biomedical engineering, Drug delivery

Correction to: *Nature Communications* 10.1038/s41467-022-32050-4, published online 26 July 2022

The original version of this Article contained an error in Figures 6k (PED + N3@PGDA7 + Laser) and 6l (PED + N3@PGDA7), in which the images were duplicates of adjacent images and the correct images were missing. This has been corrected in both the PDF and HTML versions of the Article.

