# Supplementary material for: Engineered bioorthogonal POLY-PROTAC nanoparticles for tumour-specific protein degradation and precise cancer therapy
Source: Nat Commun. 2022 Jul 26;13:4318. doi: 10.1038/s41467-022-32050-4 (PMC9325692; doi:10.1038/s41467-022-32050-4)
Supplement: Supplementary file 2 — Supplementary Information Engineered Bioorthogonal POLY-PROTAC Nanoparticles for Tumor-Specific Protein Degradation and Precise Cancer Therapy [file 41467_2022_32050_MOESM2_ESM.pdf]

Supplementary Information

Engineered Bioorthogonal POLY-PROTAC Nanoparticles for Tumor-Specific Protein Degradation and Precise Cancer Therapy

Jing Gao[1,2], Bo Hou[2,3], Qiwen Zhu[2], Lei Yang[4], Xingyu Jiang[4], Zhifeng Zou[2,3], Xutong Li[2], Tianfeng Xu[2], Mingyue Zheng[2], Yi-Hung Chen[5], Zhiai Xu[3,*], Huixiong Xu[6,*], Haijun Yu[2,4,*]

[1] Department of Medical Ultrasound and Center of Minimally Invasive Treatment for Tumor, Shanghai Tenth People's Hospital, Ultrasound Research and Education Institute, School of Medicine, Tongji University, Shanghai, 200072, China;

[2] State Key Laboratory of Drug Research & Center of Pharmaceutics, Shanghai Institute of Materia Medica, Chinese Academy of Sciences, Shanghai 201203, China;

[3] School of Chemistry and Molecular Engineering, East China Normal University, Shanghai 200241, China;

[4] School of Chinese Materia Medica, Nanjing University of Chinese Medicine, Nanjing, 210023, China

[5] Institute for Advanced Studies (IAS), Wuhan University Wuhan, 430072, China;

[6] Department of Ultrasound, Zhongshan Hospital, Institute of Ultrasound in Medicine and Engineering, Fudan University, 200032, Shanghai, China.

[*] Corresponding authors: Prof. Zhiai Xu, zaxu@chem.ecnu.edu.cn, Prof. Huixiong Xu, xuhuixiong2022@126.com; Prof. Haijun Yu, E-mail: hjyu@simm.ac.cn.

Supplementary Methods

Synthesis of ARV-771

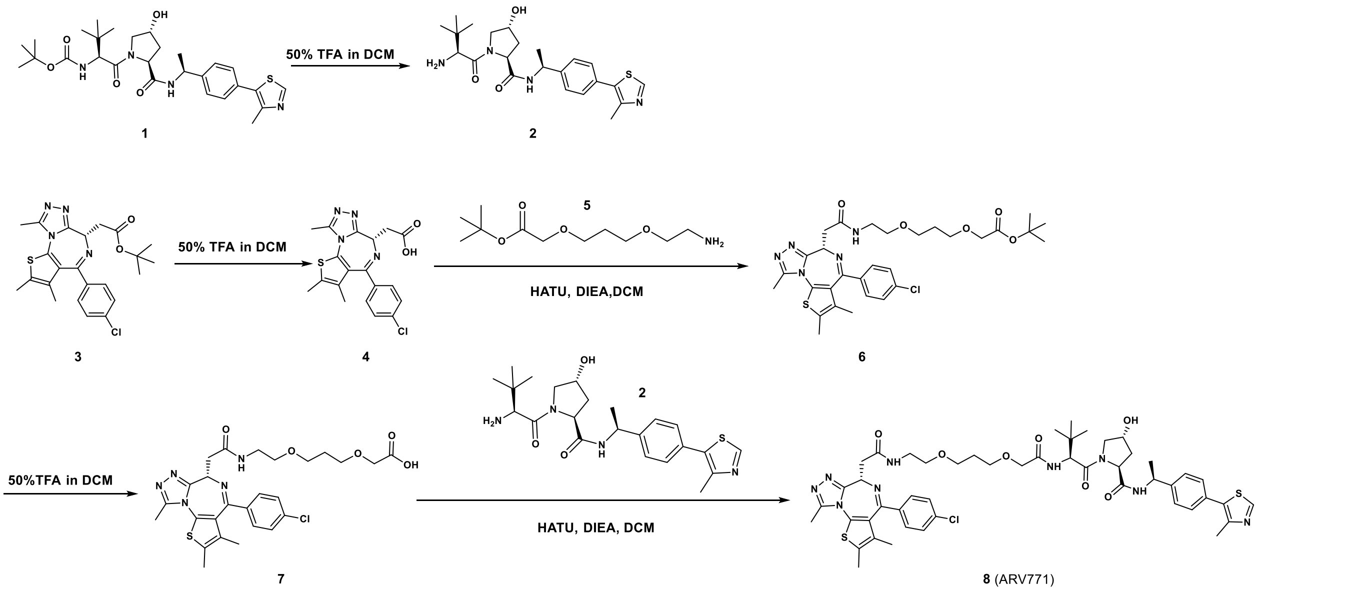

Synthesis of (2S,4R)-1-((S)-2-amino-3,3-dimethylbutanoyl)-4-hydroxy-N-((S)-1-(4-(4-methylthiazol-5-yl)phenyl)ethyl)pyrrolidine-2-carboxamide (2)

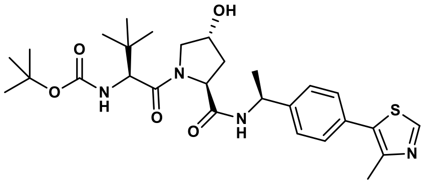

Compound **1** (commercially available, 500.0 mg, 0.92 mmol) was dissolved in DCM solution containing 50% of TFA (v/v), and stirred at room temperature for 3 h. The reaction was monitored by thin layer chromatography (TLC). The solvent was removed under pressure 3 h post-reaction. Thereafter, the crude product was redissolved in DCM and washed with water. The organic layer was collected and dried over anhydrous Na_2SO_4, the raw product was purified by silica gel chromatography (DCM: MeOH = 80:1~20:1) to obtain purified compound **2** (375.6 mg, yield 90%) as white solid. ^{1}H-NMR (CDCl3, 500 MHz) δ:1.03 (s, 9H), 1.38 (d, *J* = 7.0 Hz, 3H) 1.78 (m, 1H), 2.10 (dd, *J* = 12.95 Hz, 7.7 Hz, 1H), 2.45(s, 3H), 3.50 (dd, *J* = 10.95 Hz, 3.55 Hz, 1H), 3.67 (d, *J* = 11.05 Hz, 1H), 3.93 (s, 1H), 4.34 (s, 1H), 4.54 (m, 1H), 4.92 (m, 1H), 5.23 (d, *J* = 3.05 Hz, 1H), 7.38 (d, *J* = 8.2 Hz, 2H), 7.44 (d, *J* = 8.3 Hz, 2H), 7.99 (s, 2H), 8.56 (d, *J* = 7.8 Hz, 1H), 8.99 (s, 1H).

Synthesis of (S)-2-(4-(4-chlorophenyl)-2,3,9-trimethyl-6H-thieno[3,2-f][1,2,4]triazolo[4,3-a][1,4]diazepin-6-yl)acetic acid (4)

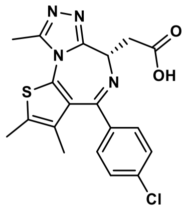

Compound **3** (commercially available, 500.0 mg, 1.1 mmol) was dissolved in 50% TFA (V/V)-containing DCM solution and stirred for 3 h at room temperature. After removal of the solution, the raw product was redissolved in DCM, washed with water, dried over anhydrous Na_2SO_4, and then purified by silica gel column chromatography (DCM: MeOH = 80:1~20:1) to obtain compound **4** as yellow solid (392.5 mg, yield 90%). ^{1}H-NMR ($CDCl_3$, 500 MHz) δ:1.09 (s, 3H), 2.42 (s, 3H), 2.71 (s, 3H), 3.59 (dd, *J* =12.55 Hz, 8.7 Hz, 1H), 3.71 (dd, *J* = 12.4 Hz,8.8 Hz, 1H), 4.62 (t, *J* = 8.7 Hz, 1H), 7.33 (d, *J* = 10.8 Hz, 2H), 7.43 (d, *J* = 10.55 Hz, 2H).

Synthesis of tert-butyl (S)-2-(3-(2-(2-(4-(4-chlorophenyl)-2,3,9-trimethyl-6H-thieno[3,2-f][1,2,4]triazolo[4,3-a][1,4]diazepin-6 yl) acetamido)ethoxy)propoxy) acetate (6)

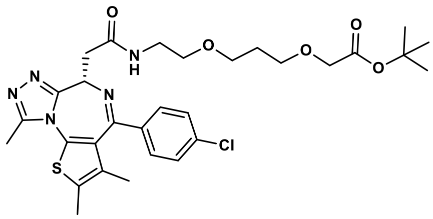

Compound **4** (200.4 mg, 0.50 mmol, 1.0 eq), compound **5** (commercially available, 128.2 mg, 0.55 mmol, 1.1 eq), HATU (239.3 mg, 0.625 mmol, 1.25 eq) and DIEA (322.5 mg, 2.5 mmol, 5.0 eq) were dissolved in DCM, and stirred overnight at room temperature. The reactant was washed with water and saturated NH_4Cl solution, and then dried with anhydrous Na_2SO_4. The crude production was purified via silica gel column chromatography (DCM: MeOH = 80:1~20:1) to obtain compound **6** as light-yellow oily liquid (180.5 mg, 59% yield). ^{1}H-NMR ($CDCl_3$, 500 MHz) δ:1.50 (s, 9H), 1.69 (s, 3H), 1.92 (m, 2H), 2.42 (m, 3H), 2.68 (s, 3H), 3.41 (dd, *J* = 14.5 Hz, 7.25 Hz, 1H), 3.49 (m, 1H), 3.56 (m, 4H), 3.61 (t, *J* = 6.3 Hz,

2H), 3.64 (t, J = 6.15 Hz, 2H), 3.99 (s, 2H), 4.66 (t, J = 7.0 Hz, 1H), 6.84 (s, 1H), 7.34 (d, J = 8.8 Hz, 2H), 7.43 (d, J = 8.55 Hz, 2H).

Synthesis of (S)-2-(3-(2-(2-(4-(4-chlorophenyl)-2,3,9-trimethyl-6H-thieno[3,2-f][1,2,4]triazolo[4,3-a][1,4]diazepin-6-yl)acetamido)ethoxy)propoxy)acetic acid (7)

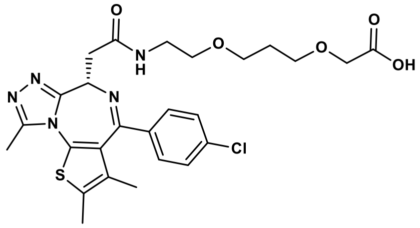

Compound **7** (180.5 mg, 0.29 mmol, 1.0 eq) was dissolved in DCM solution containing 50% TFA (V/V), and stirred at room temperature for 3 h. After removal of solution by vacuum evaporation, the crude product was diluted by DCM, washed with water, dried over anhydrous Na_2SO_4, and then purified by silica gel column chromatography (DCM: MeOH = 80:1~20:1) to obtain compound **7** as yellow solid (140.3 mg, 86% yield). ^{1}H-NMR ($CDCl_3$, 500 MHz) δ: 1.71 (s, 3H), 1.89 (s, 2H), 2.44 (s, 3H), 2.72 (s, 3H), 3.52 (m, 3H), 3.64 (t, J = 4.95 Hz, 2H), 3.70 (m, 4H), 3.77 (m, 1H), 4.13 (q, J =16.7 Hz, 2H), 4.75 (dd, J = 16.85 Hz, 6.2 Hz, 2H), 7.36 (d, J = 8.75 Hz, 2H), 7.43 (d, J = 8.45 Hz, 2H).

Synthesis of (2S,4R)-1-((S)-2-(tert-butyl)-15-((S)-4-(4-chlorophenyl)-2,3,9-trimethyl-6H-thieno[3,2-f][1,2,4]triazolo[4,3-a][1,4]diazepin-6-yl)-4,14-dioxo-6,10-dioxa-3, 13-diazapentadecanoyl)-4-hydroxy-N-((S)-1-(4-(4-methylthiazol-5-yl)phenyl) ethyl) pyrrolidine-2-carboxamide (8, ARV771)

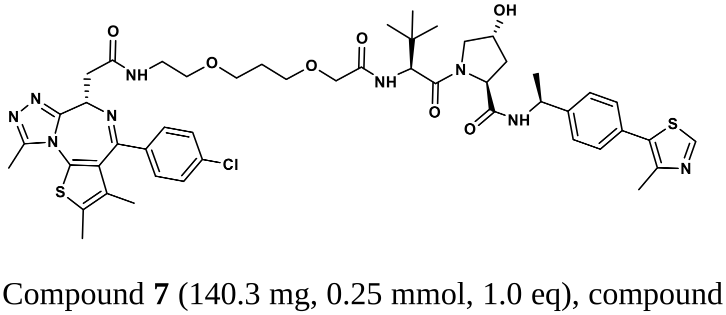

Compound **7** (140.3 mg, 0.25 mmol, 1.0 eq), compound **2** (133.2 mg, 0.30 mmol, 1.2 eq), HATU (142.5 mg, 0.38 mmol, 1.5 eq) and DIEA (161.3 mg, 1.25 mmol, 5.0 eq) were dissolved in DCM and stirred overnight. The crude product was washed with water and saturated NH_4Cl solution, dried over anhydrous Na_2SO_4 solution. After removal of solution, the raw product was then purified by silica gel column

chromatography (DCM: MeOH = 80:1~15:1) to obtain compound **8** as white solid (172.5 mg, 70% yield). ^{1}H-NMR ($CDCl_3$, 500 MHz) δ: 1.08 (s, 9H), 1.42 (d, *J* = 7.0 Hz, 3H), 1.67 (s, 3H), 1.83-1.92 (m, 2H), 2.16-2.23 (m, 5H), 2.40 (s, 3H), 2.50 (s, 3H), 2.63 (d, *J* = 7.4 Hz, 2H), 3.38-3.67 (m, 12H), 3.93 (d, *J* = 15.5 Hz, 1H), 4.03 (d, *J* = 15.5 Hz, 1H), 4.63-4.67 (m, 2H), 4.82-4.85 (m, 1H), 5.04-5.07 (m, 1H), 7.28-7.41 (m, 8H), 7.67 (d, *J* = 7.75 Hz, 2H), 8.88 (s, 1H). ^{13}C-NMR ($CDCl_3$, 125 MHz) δ: 11.8, 13.1, 14.4, 16.1, 22.1, 26.5, 29.5, 35.6, 36.5, 38.4, 39.5, 48.8, 54.2, 56.9, 57.1, 58.7, 67.6, 69.0, 69.4, 70.0, 70.2, 76.8, 77.0, 77.3, 126.5, 128.7, 129.4, 129.9 130.6, 130.8, 131.0, 131.7, 131.8, 136.5, 136.8, 143.4, 148.4, 150.0, 150.2, 155.7, 163.8, 170.3, 170.5, 170.6, 171.2. LC-MS m/z Calcd. for $C_{49}H_{60}ClN_9O_7S_2$ $[M+H]^+$ 986.4, Found 986.0.

Synthesis of ARV-771 mimic (mARV771)

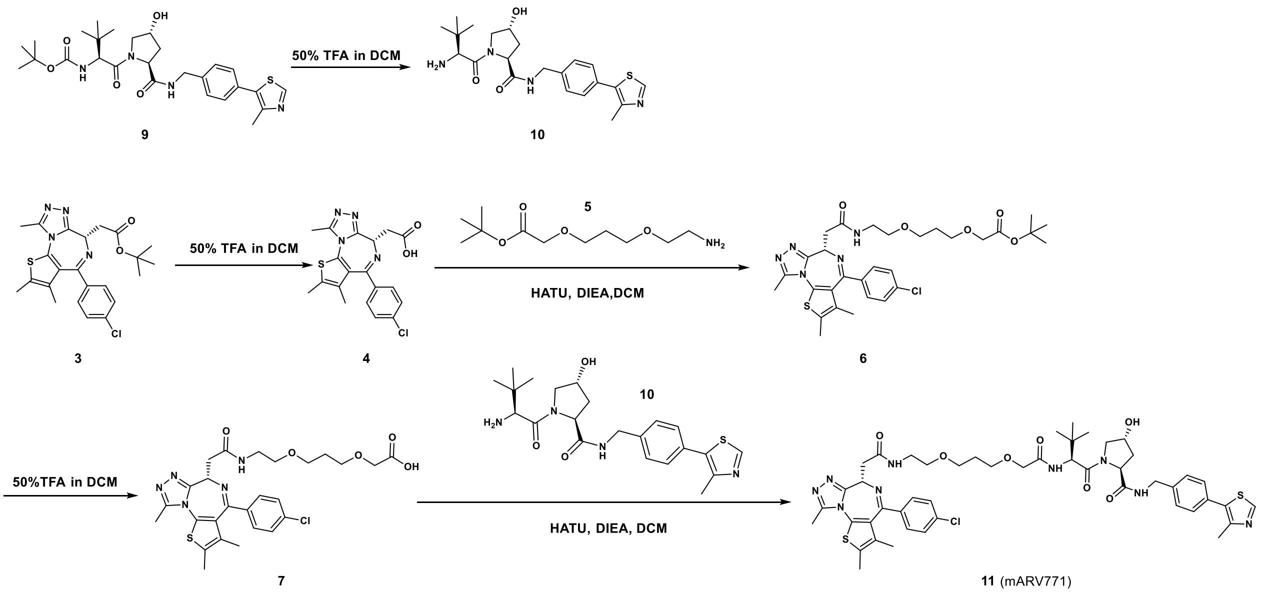

Synthesis of (2S,4R)-1-((S)-2-amino-3,3-dimethylbutanoyl)-4-hydroxy-N-(4-(4-methylthiazol-5-yl)benzyl)pyrrolidine-2-carboxamide (10)

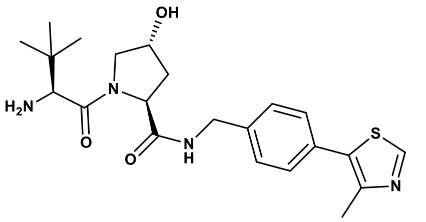

Compound **9** (commercially available, 500.0 mg, 0.94 mmol, 1.0 eq) was dissolved in DCM solution containing 50% of TFA (v/v), and stirred at room temperature for 3 h and then removed the solution by vacuum evaporation. The raw product was redissolved in DCM, washed with water, dried over anhydrous Na_2SO_4, and then purified by silica gel column chromatography (DCM: MeOH = 80:1~20:1) to obtain compound **10** as white solid (363.8 mg, 90% yield). ^{1}H-NMR ($CDCl_3$, 500 MHz) δ: 1.47 (s, 9H), 2.41 (s, 3H), 2.67 (s, 2H), 3.39 (m, 1H), 3.68 (d, *J* = 5.4 Hz, 2H), 3.74 (m, 2H), 4.04 (s, 2H), 4.66 (d, *J* = 7.0 Hz, 1H), 7.33 (d, *J* = 8.8 Hz, 2H), 7.42 (d, *J* = 8.5 Hz, 2H).

Synthesis of (2S,4R)-1-((S)-2-(tert-butyl)-15-((S)-4-(4-chlorophenyl)-2,3,9-trimethyl-6H-thieno[3,2-f][1,2,4]triazolo[4,3-a][1,4]diazepin-6-yl)-4,14-dioxo-6,10-dioxa-3,13-diazapentadecanoyl)-4-hydroxy-N-(4-(4-methylthiazol-5-yl)benzyl) pyrrolidine-2-carboxamide (11, mARV771)

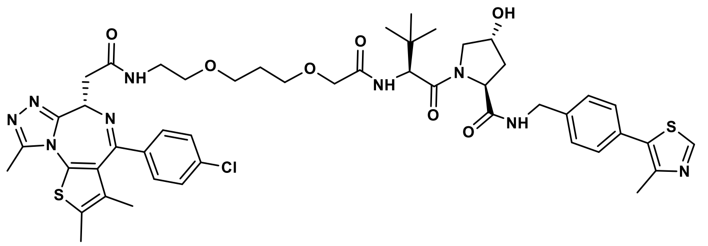

Compound **7** (140.3 mg, 0.25 mmol, 1.0 eq), compound **10** (120.5 mg, 0.30 mmol, 1.2 eq), HATU (142.5 mg, 0.38 mmol, 1.5 eq) and DIEA (161.3 mg, 1.25 mmol, 5.0 eq) were dissolved in DCM, stirred overnight at room temperature. The mixture was then washed with water and saturated NH_4Cl solution, and dried over anhydrous Na_2SO_4 solution. The crude production was further purified by silica gel column chromatography (DCM: MeOH = 80:1~15:1) to obtain compound **11** as white solid (177.2 mg, 73% yield). ^{1}H-NMR ($CDCl_3$, 500 MHz) δ: 1.02 (s, 9H), 1.65 (s, 3H), 1.78 (s, 2H), 1.96 (d, *J* = 9.8 Hz, 2H), 2.21 (m, 2H), 2.40 (s, 3H), 2.50 (s, 3H), 2.62 (s, 3H), 3.32-3.71 (m, 10H), 3.92-4.04 (m, 2H), 4.44 (dd, *J* = 15.3 Hz, 6.4 Hz, 1H), 4.54 (s, 1H), 4.64 (dd, *J* = 8.05 Hz, 6.0 Hz, 1H), 4.68 (d, *J* =9.35 Hz, 1H), 4.83 (t, *J* = 8.1 Hz, 1H), 7.23-7.36 (m, 8H), 7.60-7.66 (m, 2H), 8.67 (s, 1H). ^{13}C-NMR ($CDCl_3$, 125 MHz) δ: 11.8, 12.0, 13.1, 14.4, 16.1, 17.4, 18.6, 26.5, 29.3, 29.7, 30.3, 35.6, 37.1, 38.1, 39.6, 41.9, 42.9, 53.7, 54.2, 56.9, 57.2, 59.0, 67.8, 68.2, 69.3, 70.2, 70.3, 76.8, 77.1, 77.3, 127.8, 128.7, 129.2, 130.0, 130.8, 131.1, 131.7, 131.9, 136.5,

136.9, 138.4, 148.3, 150.0, 150.2, 155.7, 164.0, 170.4, 170.6, 171.1, 171.6. LC-MS m/z Calcd. for $C_{48}H_{58}ClN_9O_7S_2$ $[M+H]^+$ 972.4, Found 972.1.

Synthesis of MZ1

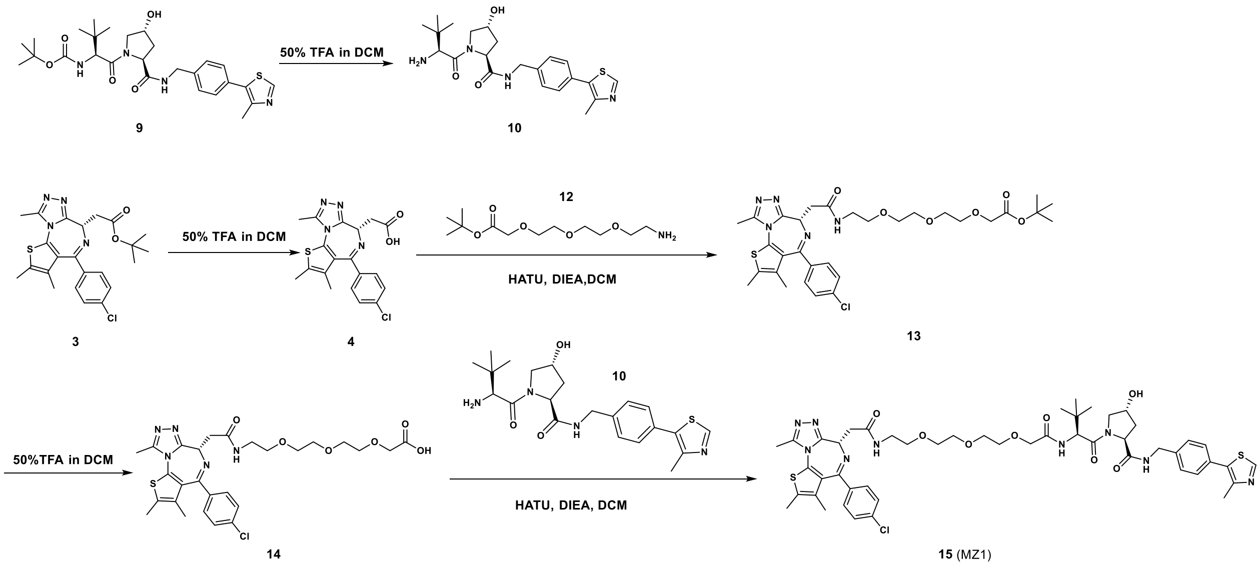

Synthesis of tert-butyl (S)-1-(4-(4-chlorophenyl)-2,3,9-trimethyl-6H-thieno[3,2-f][1,2,4]triazolo[4,3-a][1,4]diazepin-6-yl)-2-oxo-6,9,12-trioxa-3-azatetradecan-14-oate (13)

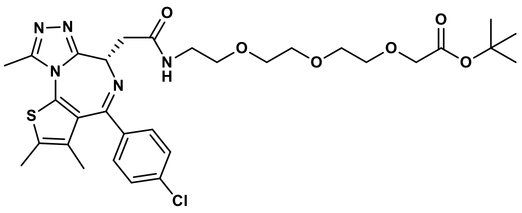

Compound **4** (200.4 mg, 0.50 mmol, 1 eq), compound **12** (commercially available, 154.5 mg, 0.55 mmol, 1.1 eq), HATU (239.3 mg, 0.625 mmol, 1.25 eq) and DIEA (322.5 mg, 2.5 mmol, 5.0 eq) were dissolved in DCM, and stirred overnight at room temperature. The reactive solution was then diluted with DCM, washed with water and saturated NH_4Cl solution, and then dried over anhydrous Na_2SO_4. After removal of solution, the crude product was purified via silica gel column chromatography (DCM: MeOH = 80:1~20:1) to obtain compound **6** as light-yellow oily liquid (177.5 mg, 55% yield). ^{1}H-NMR ($CDCl_3$, 500 MHz) δ: 1.04 (s, 9H), 1.25 (s, 3H), 2.27 (s, 3H), 2.51 (s, 3H), 3.58-3.60 (m, 2H), 3.82-4.21 (m, 10H), 4.32 (dd, *J* = 15.05 Hz, 5.6 Hz, 2H), 4.50 (s, 2H), 4.58 (m, 2H), 7.32 (d, *J* = 8.35 Hz, 2H), 7.35 (d, *J* = 8.35 Hz, 2H), 8.87 (s, 1H).

Synthesis of (S)-1-(4-(4-chlorophenyl)-2,3,9-trimethyl-6H-thieno[3,2-f][1,2,4]triazolo[4,3-a][1,4]diazepin-6-yl)-2-oxo-6,9,12-trioxa-3-azatetradecan-14-oic acid (14)

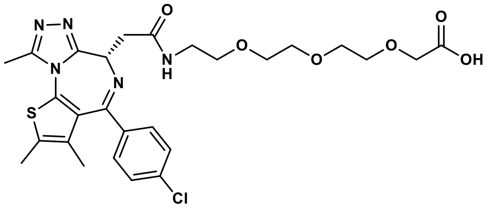

Compound **13** (177.5 mg, 0.275 mmol) was dissolved in DCM containing 50% of TFA (v/v), and stirred at room temperature for 3 h and then removed the solvent and TFA by vacuum evaporation. Then, the crude production was diluted with DCM, washed with water, dried over anhydrous Na_2SO_4, and then purified by silica gel column chromatography (DCM: MeOH = 80:1~20:1) to give compound **14** as light-yellow solid (132.7 mg, 82% yield). ^{1}H-NMR ($CDCl_3$, 500 MHz) δ: 1.69 (s, 3H), 2.41 (s, 3H), 2.68 (s, 3H), 3.47 (m, 2H), 3.57-3.77 (m, 12H), 4.18 (m, 2H), 4.73 (t, J = 7.05 Hz, 1H), 7.33 (d, J = 8.8 Hz, 2H), 7.41 (d, J = 8.5 Hz, 2H), 7.74 (t, J = 5.1 Hz, 1H).

Synthesis of (2S,4R)-1-((S)-2-(tert-butyl)-17-((S)-4-(4-chlorophenyl)-2,3,9-trimethyl-6H-thieno[3,2-f][1,2,4]triazolo[4,3-a][1,4]diazepin-6-yl)-4,16-dioxo-6,9,12-trioxa-3,15-diazaheptadecanoyl)-4-hydroxy-N-(4-(4-methylthiazol-5-yl)benzyl) pyrrolidine-2-carboxamide (15, MZ1)

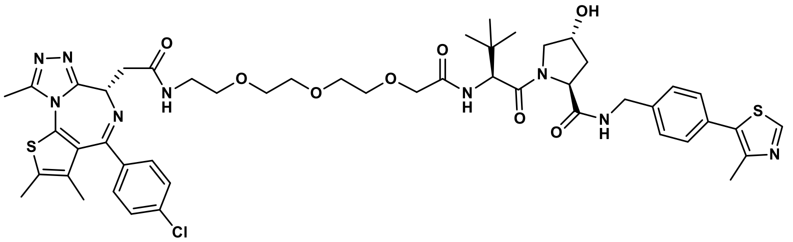

Compound **14** (132.7 mg, 0.23 mmol, 1.0 eq), compound **10** (108.5 mg, 0.27 mmol, 1.2 eq), HATU (127.5 mg, 0.34 mmol, 1.5 eq) and DIEA (145.8 mg, 1.13 mmol, 5.0 eq) were dissolved in DCM, and then stirred overnight. The mixture was washed through water and saturated NH_4Cl solution, dried over anhydrous Na_2SO_4 solution, filtered. And then the raw product was purified by silica gel column chromatography (DCM: MeOH = 80:1~15:1) to obtained compound **15** as white solid (168.8 mg, 75% yield). ^{1}H-NMR ($CDCl_3$, 500 MHz) δ: 1.07 (s, 9H), 1.47 (s, 3H), 1.67 (s, 3H), 2.42 (m, 2H), 2.40-2.45 (m, 4H), 2.52 (s, 3H), 2.63 (s, 3H), 3.36-3.40 (m, 2H), 3.48-3.58 (m, 2H), 3.61-3.74 (m, 12H), 4.07-4.11 (m, 1H), 4.30 (m,

1H), 4.46 (m, 1H), 4.67 (m, 1H), 4.73 (d, J = 9.3 Hz, 1H), 4.81 (t, J = 7.7 Hz, 1H), 5.08 (t, J = 7.2 Hz, 1H), 7.30-7.39 (m, 8H), 7.41 (m, 1H), 7.54 (s, 1H), 7.86 (s, 1H), 8.67 (s, 1H). ^{13}C-NMR ($CDCl_3$, 125 MHz) δ: 11.8, 13.1, 14.4, 16.1, 22.2, 26.5, 35.6, 35.8, 38.1, 39.8, 48.8, 54.2, 56.8, 57.1, 58.7, 69.9, 70.2, 70.3, 70.4, 70.8, 71.6, 76.8, 77.0, 77.3, 126.5, 128.7, 129.5, 130.0, 130.7, 130.8,130.9, 131.1, 131.9, 136.5, 136.8, 143.4, 148.5, 149.8, 150.2, 155.9, 163.9, 170.0, 170.8, 170.9, 171.4. LC-MS m/z Calcd. for $C_{49}H_{60}ClN_9O_8S_2$ $[M+H]^+$ 1002.4, Found 1002.1.

Synthesis of MZ1 mimic (16, mMZ1)

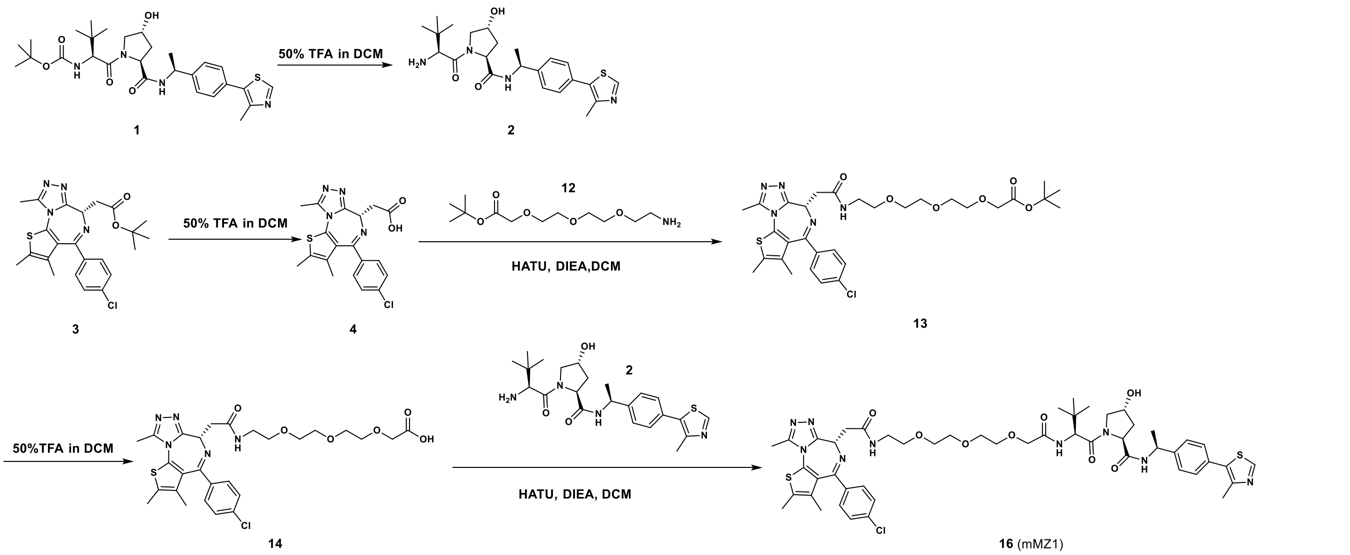

Synthesis of (2S,4R)-1-((S)-2-(tert-butyl)-17-((S)-4-(4-chlorophenyl)-2,3,9-trimethyl-6H-thieno[3,2-f][1,2,4]triazolo[4,3-a][1,4]diazepin-6-yl)-4,16-dioxo-6,9,12-trioxa-3,15-diazaheptadecanoyl)-4-hydroxy-N-((S)-1-(4-(4-methylthiazol-5-yl) phenyl)ethyl)pyrrolidine-2-carboxamide (16, mMZ1)

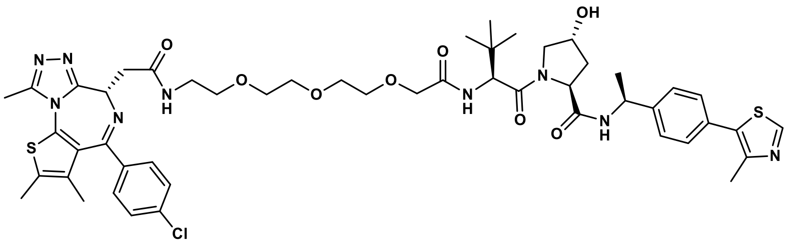

Compound **14** (132.7 mg, 0.23 mmol, 1.0 eq), compound **2** (120.4 mg, 0.27 mmol, 1.2 eq), HATU (127.5 mg, 0.34 mmol, 1.5 eq) and DIEA (145.8 mg, 1.13 mmol, 5.0 eq) were dissolved in DCM and stirred overnight. The reaction solution was washed with water and saturated NH_4Cl solution, then dried over anhydrous Na_2SO_4 solution. The crude product was purified by silica gel column chromatography (DCM:

MeOH = 80:1~15:1) to obtain compound **16** as white solid (166.4 mg, 72% yield). ^{1}H-NMR ($CDCl_3$, 500 MHz) δ: 1.03 (s, 9H), 1.43 (d, *J* = 6.95 Hz, 1H), 1.66 (s, 3H), 2.22 (m, 1H), 2.39 (s, 3H), 2.44 (m, 1H), 2.49 (m, 1H), 2.52 (s, 3H), 2.59 (m, 1H), 2.65 (s, 3H), 3.46-3.48 (m, 2H), 3.53-3.58 (m, 14H), 4.12-4.16 (m, 1H), 4.45 (s, 1H), 4.66 (m, 1H), 4.69-4.76 (m, 2H), 5.07 (m, 1H), 7.23-7.39 (m, 8H), 7.41 (m, 1H), 7.58 (d, *J* = 7.8 Hz, 1H), 7.63 (s, 1H), 8.67 (s, 1H). ^{13}C-NMR ($CDCl_3$, 125 MHz) δ: 8.68, 11.3, 12.7, 13.2, 13.7, 14.0, 15.6, 18.6, 22.2, 26.0, 28.9, 29.0, 29.3, 29.9, 30.0, 31.5, 35.1, 35.5, 36.1, 37.6, 39.2, 42.6, 42.9, 45.0, 53.0, 53.7, 56.3, 56.7, 58.5, 64.4, 65.7, 69.5, 69.8, 69.9, 70.0, 76.4, 76.6, 76.8, 127.5, 128.3, 128.9, 130.0, 130.2, 130.5, 130.6, 131.3, 131.4, 136.1, 136.3, 137.9, 147.9, 149.4, 149.9, 155.3, 163.5, 169.4, 170.3, 170.6, 171.0. LC-MS m/z Calcd. for $C_{50}H_{62}ClN_9O_8S_2$ $[M+H]^+$ 1016.4, Found 1016.1.

Synthesis of ARV771 and MZ1 methacrylate (Me-ARV771 and Me-MZ1)

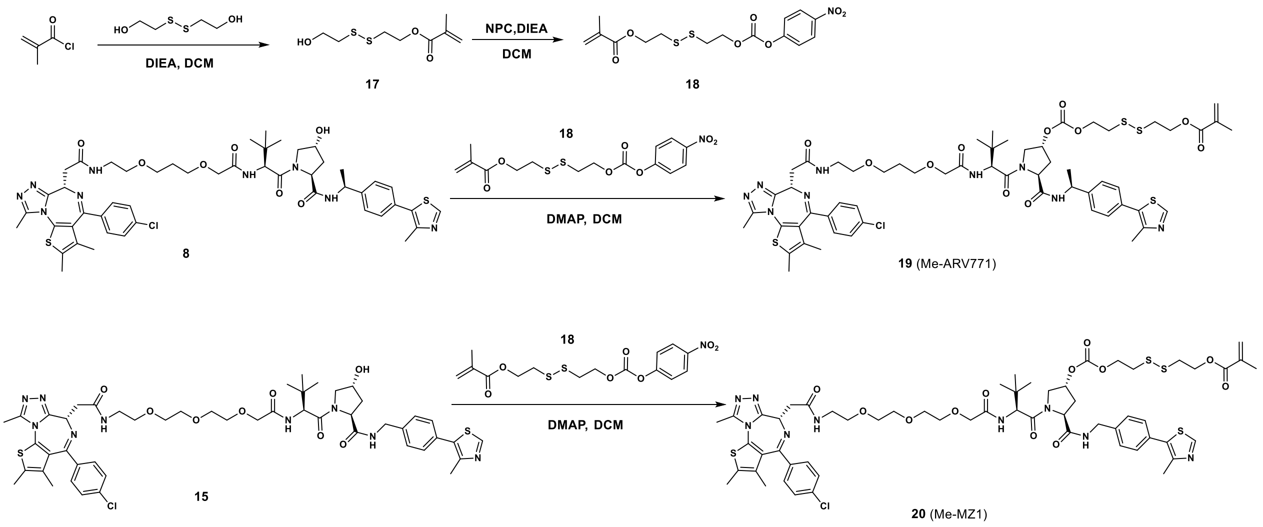

Synthesis of 2-((2-hydroxyethyl) disulfaneyl)ethyl methacrylate (17) and 2-((2-(((4-nitrophenoxy)carbonyl)oxy)ethyl)disulfaneyl)ethyl methacrylate (18)

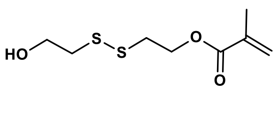

2,2'-disulfanediylbis(ethan-1-ol) (commercially available, 308.0 mg, 2.0 mmol, 1.0 eq) and DIEA (775.4 mg, 6.0 mmol, 3.0 eq) were dissolved in DCM. Then, the solution of methacryloyl chloride (commercially available, 208.0 mg, 2.0 mmol, 1.0 eq) in DCM was added dropwise into the mixture, and stirred for 24 h. Then, the mixture solution was washed with water and saturated NH_4Cl solution and the organic solvent

was collected and dried by anhydrous Na_2SO_4. The crude product was further depurated by silica gel column chromatography (n-hexane: ethyl acetate = 4:1) to give compound **17** as light-yellow oily liquid (310.8 mg, 70% yield). ^{1}H-NMR ($CDCl_3$, 500 MHz) δ: 1.95 (s, 3H), 2.89 (t, *J* = 5.8 Hz, 2H), 2.98 (t, *J* = 6.7 Hz, 2H), 3.98 (m, 2H), 4.43 (m, 3H), 5.60 (s, 1H), 6.14 (s, 1H).

Synthesis of 2-((2-(((((3R,5S)-1-((S)-2-(tert-butyl)-15-((S)-4-(4-chlorophenyl)-2,3,9-trimethyl-6H-thieno[3,2-f][1,2,4]triazolo[4,3-a][1,4]diazepin-6-yl)-4,14-dioxo-6,10-dioxa-3,13-diazapentadecanoyl)-5-(((S)-1-(4-(4-methylthiazol-5-yl)phenyl) ethyl)carbamoyl)pyrrolidin-3-yl)oxy)carbonyl)oxy)ethyl) disulfaneyl) ethyl methacrylate (19, Me-ARV771)

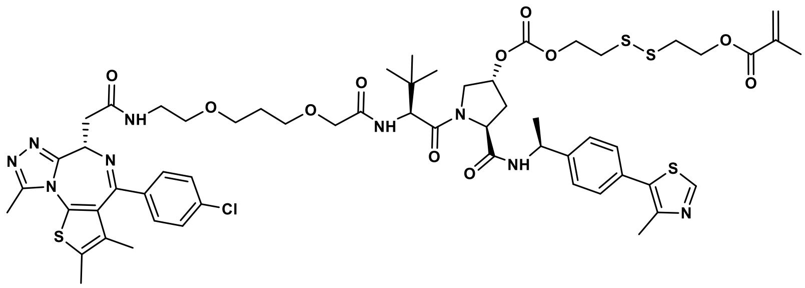

First, to synthesize 2-((2-(((4-nitrophenoxy)carbonyl)oxy)ethyl)disulfaneyl)ethyl methacrylate (**18**), the bis(4-nitrophenyl) carbonate (commercially available, 851.3 mg, 2.8 mmol, 1.2 eq) and DIEA (1085.6 mg, 8.4 mmol, 3 eq) were dissolved in DCM, and then the DCM solution of compound **17** (310.8 mg, 1.4 mmol, 1 eq) was added dropwise into above solution, stirred under argon protection at room temperature for 6 h. The reaction was monitored by TLC analysis and stopped continued for 6 h. The solution was diluted by DCM, and then washed by water and NH_4Cl saturated solution, dried over anhydrous Na_2SO_4 to obtain raw production by filtration.

compound **8** (453.6 mg, 0.46 mmol, 1.0 eq), compound **18** (267.1 mg, 0.69 mmol, 1.5 eq), DMAP (84.18 mg, 0.69 mmol, 1.5 eq) and DIEA (118.7 mg, 0.92 mmol, 2.0 eq) were dissolved in DCM, and stirred overnight at room temperature. The mixture was washed with water and saturated NH_4Cl solution, dried over anhydrous Na_2SO_4, and purified by silica gel column chromatography (DCM: MeOH = 80:1~15:1) to give compound **19** as white solid (380.1 mg, 67% yield). ^{1}H-NMR ($CDCl_3$, 500 MHz) δ: 1.08 (s, 9H), 1.37 (d, *J* = 6.95 Hz, 1H), 1.69 (s, 3H), 1.82 (s, 3H), 1.84-1.94 (m, 5H), 2.40 (s, 3H), 2.49 (s, 3H), 2.58

(m, 4H), 2.96 (m, 4H), 3.86-4.01 (m, 11H), 4.30 (m, 1H), 4.39-4.42 (m, 4H), 4.63-4.67 (m, 2H), 4.86 (t, *J* = 7.7 Hz, 1H), 5.04 (m, 1H), 5.26 (m, 1H), 5.58 (s, 1H), 6.13 (s, 1H), 7.23-7.32 (m, 8H), 7.41 (m, 2H), 7.89 (d, *J* = 7.75 Hz, 1H), 8.65 (s, 1H). ^{13}C-NMR ($CDCl_3$, 125 MHz) δ: 11.7, 13.1, 14.4, 16.1, 18.3, 21.9, 26.5, 29.4, 30.7, 33.4, 35.6, 36.9, 37.3, 38.7, 39.7, 48.9, 53.8, 54.3, 56.5, 58.4, 62.5, 62.9, 65.9, 68.0, 69.1, 69.5, 70.2, 76.7, 77.1, 77.3, 126.1, 126.5, 128.7, 129.4, 129.9, 130.6, 130.8, 131.0, 131.6, 131.9, 136.0, 136.6, 136.8, 143.2, 148.4, 149.8, 150.2, 154.2, 155.8, 163.5, 167.2, 169.6, 169.8, 170.7, 171.0. LC-MS m/z Calcd. for $C_{58}H_{72}ClN_9O_{11}S_4$ $[1/2M+H]^+$ 617.7, Found 617.4.

Synthesis of 2-((2-(((((3R,5S)-1-((S)-2-(tert-butyl)-17-((S)-4-(4-chlorophenyl)-2,3,9-trimethyl-6H-thieno[3,2-f][1,2,4]triazolo[4,3-a][1,4]diazepin-6-yl)-4,16-dioxo-6,9,12-trioxa-3,15-diazaheptadecanoyl)-5-((4-(4-methylthiazol-5-yl)benzyl) carbamoyl)pyrrolidin-3-yl)oxy)carbonyl)oxy)ethyl)disulfaneyl)ethyl methacrylate (20, Me-MZ1)

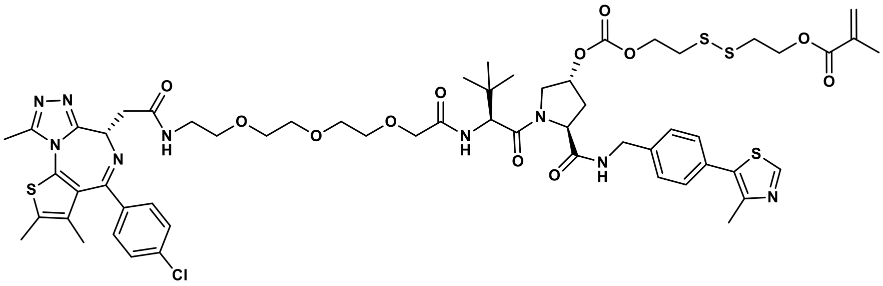

Compound **15** (450.5 mg, 0.45 mmol, 1.0 eq), compound **18** (263.2 mg, 0.68 mmol, 1.5 eq), DMAP (83.0 mg, 0.68 mmol, 1.5 eq) and DIEA (116.1 mg, 0.90 mmol, 2.0 eq) were dissolved in DCM, stirred overnight at room temperature. After the reaction finished, the mixture solution was washed by water and saturated NH_4Cl solution, dried over anhydrous Na_2SO_4, filtered and purified by silica gel column chromatography (DCM: MeOH = 80:1~15:1) to give compound **20** as white solid (365.4 mg, 65% yield). ^{1}H-NMR ($CDCl_3$, 500 MHz) δ: 0.99 (s, 9H), 1.67 (s, 3H), 1.94 (s, 3H), 2.36 (m, 1H), 2.39 (s, 3H), 2.50 (s, 3H), 2.59 (s, 3H), 2.61 (m, 1H), 2.96 (m, 4H), 3.39 (m, 2H), 3.47 (m, 2H), 3.51-3.77 (m, 14H), 4.34 (m, 1H), 4.40 (t, *J* = 6.65 Hz, 4H), 4.47-4.56 (m, 2H), 4.63 (m, 1H), 4.87 (t, *J* = 7.75 Hz, 1H), 5.58 (s, 1H), 6.12 (s, 1H), 7.25-7.34 (m, 8H), 7.38-7.40 (m, 2H), 7.96 (d, *J* = 5.9 Hz, 1H), 8.67 (s, 1H). ^{13}C NMR ($CDCl_3$, 125 MHz) δ: 11.7, 13.1, 14.4, 16.1, 18.3, 26.4, 29.7, 30.3, 33.7, 35.4, 36.8, 37.2, 38.6, 39.6, 43.1, 53.8, 54.3, 56.7, 58.6,

62.5, 65.9, 70.0, 70.2, 70.6, 70.9, 76.8, 77.0, 77.3, 126.1, 128.0, 128.7, 129.4, 130.6, 130.8, 131.0, 131.7, 132.0, 136.0, 136.6, 136.7, 138.3, 148.4, 149.8, 150.3, 154.2, 155.7, 163.7, 167.2, 170.0, 170.7, 170.8, 170.9. LC-MS m/z Calcd. for $C_{58}H_{72}ClN_9O_{12}S_4$ $[1/2M+H]^+$ 625.7, Found 625.5.

Synthesis of GSH-insensitive ARV771 methacrylate (Me-O-ARV771)

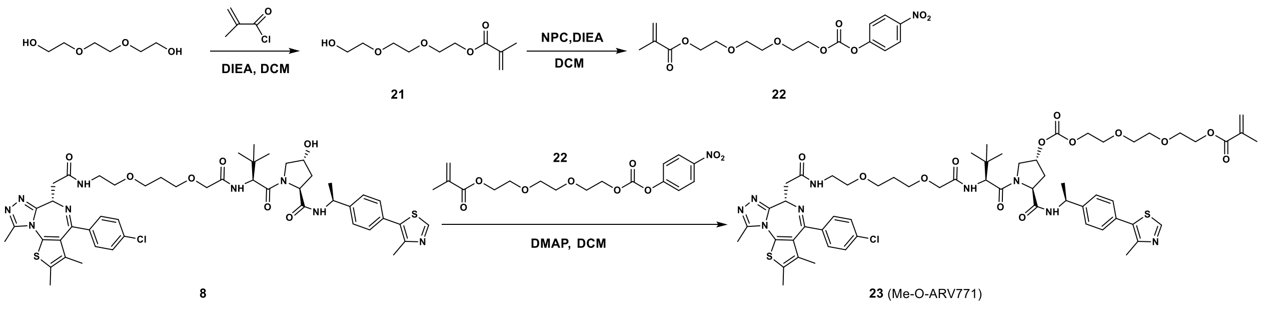

Synthesis of 2-(2-(2-hydroxyethoxy)ethoxy)ethyl methacrylate (21)

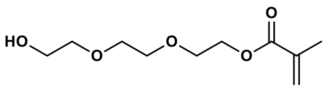

The 2,2'-(ethane-1,2-diylbis(oxy))bis(ethan-1-ol) (commercially available, 300.2 mg, 2.0 mmol, 1.0 eq) and DIEA (775.4 mg, 6.0 mmol, 3.0 eq) were dissolved in DCM. Then, the solution of methacryloyl chloride (commercially available, 208.0 mg, 2.0 mmol, 1.0 eq) in DCM was added dropwise into the mixture, and stirred for 24 h. The reaction solution was washed by water and saturated NH_4Cl solution. Then, the organic solvent was collected and dried by anhydrous Na_2SO_4. The raw production was further depurated by silica gel column chromatography (n-hexane: ethyl acetate = 4:1) to give compound **17** as colorless oily liquid (318.5 mg, 73% yield). ^{1}H-NMR ($CDCl_3$, 500 MHz) δ: 1.95 (s, 3H), 3.60-3.68 (m, 6H), 3.73 (m, 2H), 3.76 (m, 2H), 4.32 (m, 2H), 6.16 (s, 1H), 6.58 (s, 1H).

Synthesis of 2-(2-(2-(((((3R,5S)-1-((S)-2-(tert-butyl)-15-((S)-4-(4-chlorophenyl)-2,3,9-trimethyl-6H-thieno[3,2-f][1,2,4]triazolo[4,3-a][1,4]diazepin-6-yl)-4,14-dioxo-6,10-dioxa-3,13-diazapentadecanoyl)-5-(((S)-1-(4-(4-methylthiazol-5-yl)phenyl)ethyl)carbamoyl)pyrrolidin-3-yl)oxy)carbonyl)oxy)ethoxy)ethoxy)ethyl methacrylate (23, Me-O-ARV771)

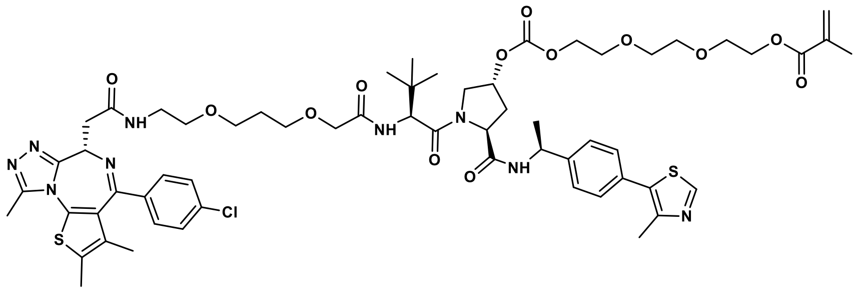

Firstly, the 2-(2-(2-(((4-nitrophenoxy) carbonyl)oxy)ethoxy)ethoxy)ethyl methacrylate (**22**) was synthesized. The bis(4-nitrophenyl) carbonate (commercially available, 851.3 mg, 2.8 mmol, 1.2 eq) and DIEA (1085.6 mg, 8.4 mmol, 3.0 eq) were dissolved in DCM, and then the solution of compound **21** (305.4 mg, 1.4 mmol, 1.0 eq) in DCM was added dropwise into above solution. The reaction was continued for 6 h with the stir steadily under argon protection at room temperature. After the reaction completed, the mixture was diluted by DCM, and subsequently washed with water and saturated NH_4Cl solution, dried over anhydrous Na_2SO_4.

Afterwards, compound **8** (98.6 mg, 0.10 mmol, 1.0 eq), compound **22** (58.1 mg, 0.15 mmol, 1.5 eq), DMAP (18.3 mg, 0.15 mmol, 1.5 eq) and DIEA (25.8 mg, 0.20 mmol, 2.0 eq) were dissolved in DCM, and stirred overnight at room temperature. The mixture was then washed with water and saturated NH_4Cl solution. The organic phase was collected and dried over anhydrous Na_2SO_4. Afterwards, the raw product was purified by silica gel column chromatography (DCM: MeOH = 80:1~15:1) to gain compound **23** as white solid (81.1 mg, 66% yield). ^{1}H-NMR ($CDCl_3$, 500 MHz) δ: 1.07 (s, 9H), 1.25 (s, 3H), 1.38 (d, J = 6.9 Hz, 1H), 1.53 (s, 1H),1.67 (s, 1H), 1.69 (s, 3H), 1.95 (s, 3H), 2.35 (m, 2H), 2.49 (s, 3H), 2.56 (m, 2H), 2.61 (s, 3H), 3.43-4.02 (m, 20H), 4.22-4.34 (m, 4H), 4.66 (m, 2H), 4.84 (m, 1H), 5.04(m, 1H), 5.26 (m, 1H), 5.57 (s, 1H), 6.12 (s, 1H), 7.28-7.42 (m, 8H), 7.41 (m, 2H), 7.87 (m, 1H), 8.66 (s, 1H). ^{13}C-NMR ($CDCl_3$, 125 MHz) δ: 11.7, 13.1, 14.4, 16.1, 21.9, 26.5, 26.9, 29.2, 29.7, 30.1, 34.0, 35.8, 38.4, 39.6, 48.8,

54.2, 56.7, 58.7, 67.9, 69.2, 69.4, 70.3, 73.3, 76.8, 77.0, 77.3, 126.4, 128.7, 129.3, 129.9, 130.5, 130.9, 131.0, 131.2, 131.7, 136.4, 136.9, 143.3, 148.4, 150.0, 150.2, 155.8, 163.7, 170.0, 170.7, 170.8, 172.2. ESI m/z Calcd. for $C_{60}H_{76}ClN_9O_{13}S_2$ $[M+H]^+$ 1230.5, Found 1230.7. Calcd. for $C_{60}H_{76}ClN_9O_{13}S_2$ $[M+Na]^+$ 1252.5, Found 1252.8.

Synthesis of macromolecular chain transfer agents mPEG$_{113}$-CTA (26), mPEG$_{113}$-GALGLPG-CTA (27) and N$_3$-PEG$_{113}$-GALGLPG-CTA (30)

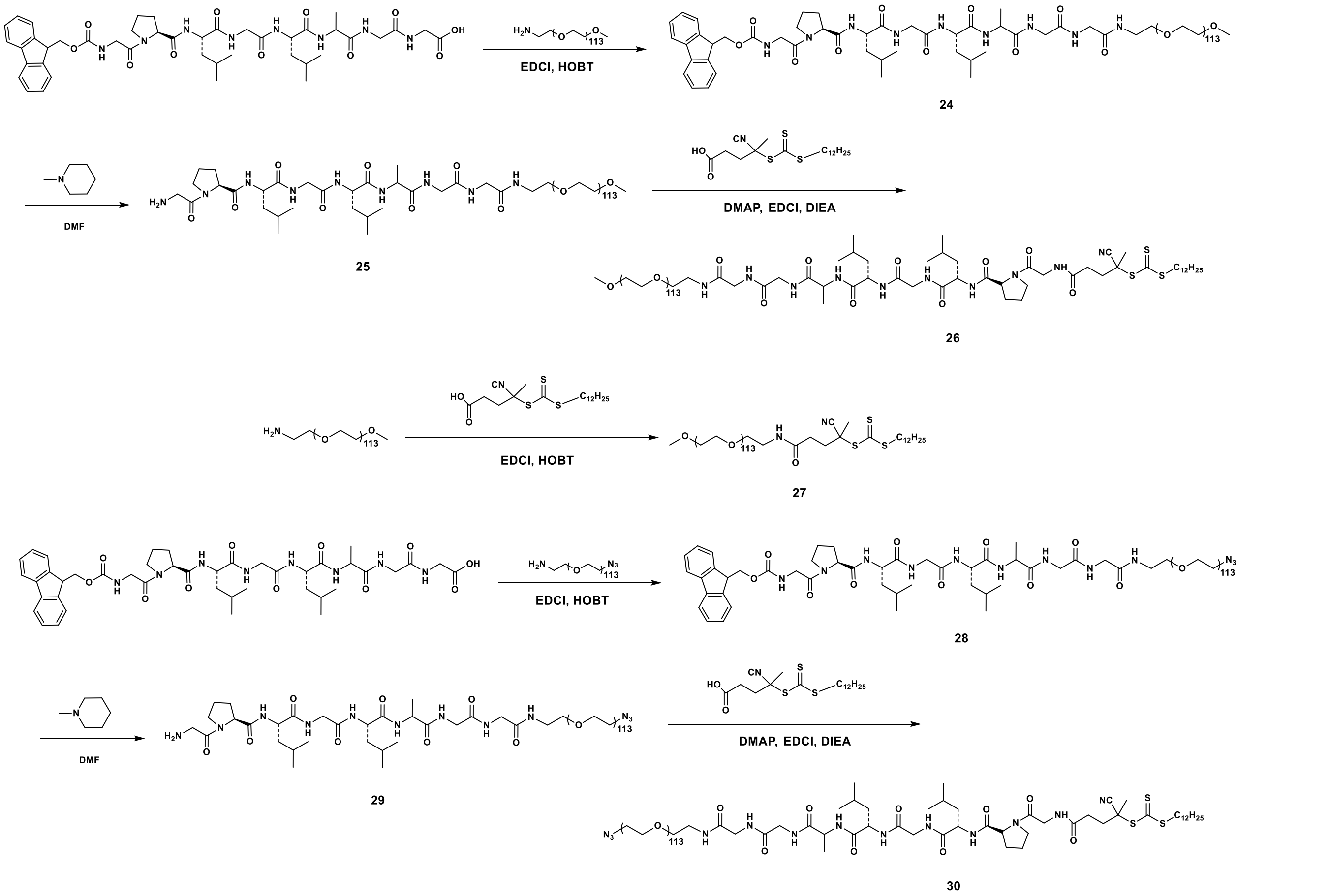

Synthesis of mPEG$_{113}$-CTA chain transfer agent (27)

CDP (121.10 mg, 0.30 mM), EDCI (115.1 mg, 0.60 mM), HOBT (81.07 mg, 0.60 mM) and DIEA (77.54 mg, 0.60 mM) were dissolved in anhydrous DMF and reacted for 90 min. Then, mPEG$_{113}$-NH$_2$ (500.0 mg, 0.10 mM) was dissolved in anhydrous DMF and added into above reactant solution. After 24 h reaction at RT, the crude product was dialyzed against ethanol and DI water. The yellowish end-product was obtained by lyophilization.

Synthesis of $mPEG_{113}$-GALGLPG-CTA (26) and N_3-PEG_{113}-GALGLPG-CTA (30)

To synthesize $mPEG_{113}$-GPLGLAG and N_3-PEG_{113}-GALGLPG, Fmoc-GPLGLAG (250.0 mg, 0.30 mM), EDCI (72.40 mg, 0.37 mM), HOBT (51.01 mg, 0.37 mM) and DIEA (77.54 mg, 0.60 mM) were dissolved in anhydrous DMF, and stirred about 90 min to activate the carboxyl group. Then, $mPEG_{113}$-NH_2 (500.0 mg, 0.10 mM) or N_3-PEG_{113}-NH_2 (500.0 mg, 0.10 mM) was dissolved in anhydrous DMF and dropwise added into above solution. The reaction was continued for 24 h at room temperature. Next, 20% (v/v) 4-Methylpiperidine was added into above mentioned mixture and stirred overnight to remove the Fmoc group. The crude product was purified by dialyzing against ethanol and DI water, and then lyophilized to obtain final product as off-white powder.

Next, CDP (121.1 mg, 0.30 mM), EDCI (115.1 mg, 0.60 mM), HOBT (81.07 mg, 0.60 mM) and DIEA (77.54 mg, 0.60 mM) were dissolved in anhydrous DMF and the mixture solution was stirred for 90 min. Then, $mPEG_{113}$-GALGLPG (566.0 mg, 0.10 mM) or N_3-PEG_{113}-GALGLPG (566.0 mg, 0.10 mM) were dissolved in anhydrous DMF and added into above reactant solution. After 24 h reaction at RT, the crude product was dialyzed against ethanol and DI water. The yellowish end-product was obtained by lyophilization. The chemical structure of the resultant products was validated by ^{1}H-NMR spectra.

Synthesis of GSH-liable POLY-PROTACs of ARV771

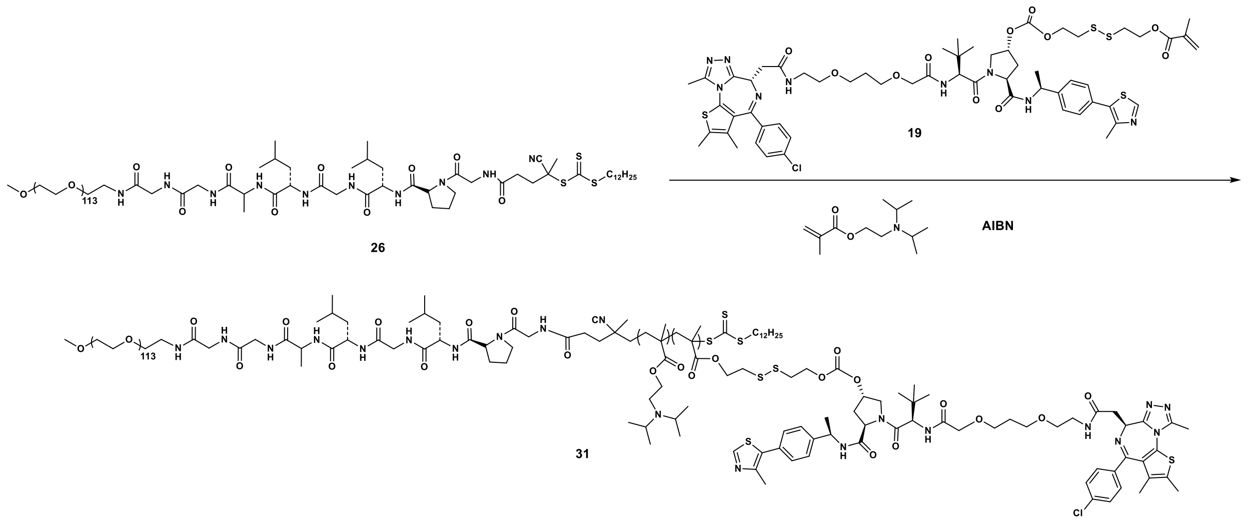

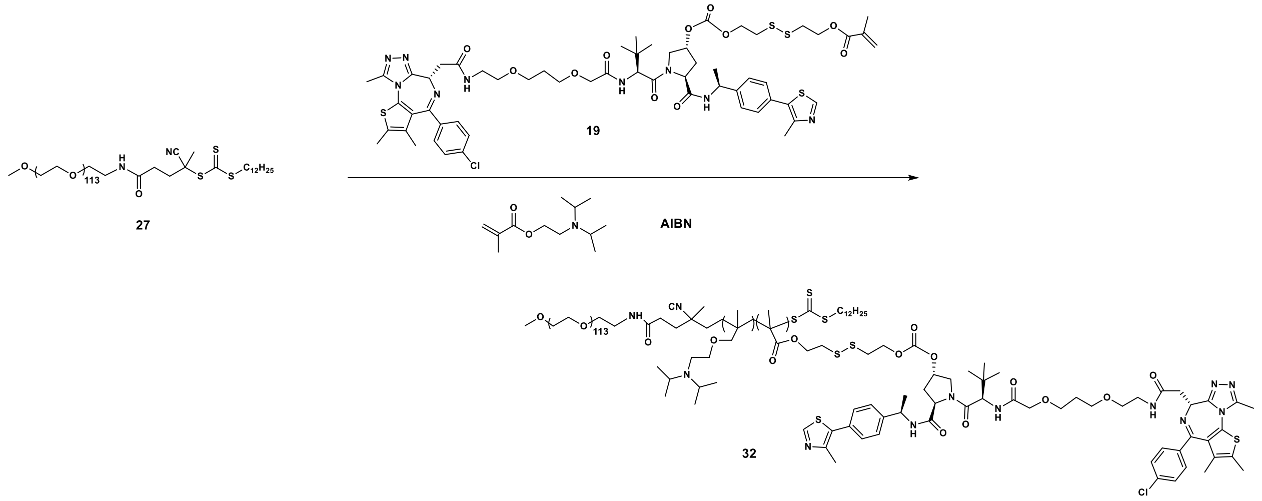

The reduction-sensitive POLY-PROTACs of ARV771 were prepared by reversible addition-fragmentation chain transfer (RAFT) polymerization. Briefly, mPEG$_{113}$-CTA (200.0 mg, 0.037 mmol) or mPEG$_{113}$-GALGLPG-CTA (200.0 mg, 0.033 mmol), DPA (466.2 mg, 2.2 mmol), Me-ARV771 (182.5 mg, 0.15 mmol) and AIBN (0.54 mg, 0.0037 mmol) were dissolved in 1.5 mL of anhydrous DMF. The polymerization for continued 24 h under 70 °C. The product was purified via dialyzing against DI water and lyophilization. The chemical structure and molecular weights of the resultant products were examined by ^{1}H-NMR spectra and gel permeability chromatography (GPC), respectively.

Synthesis of GSH-activatable POLY-PROTACs of MZ1

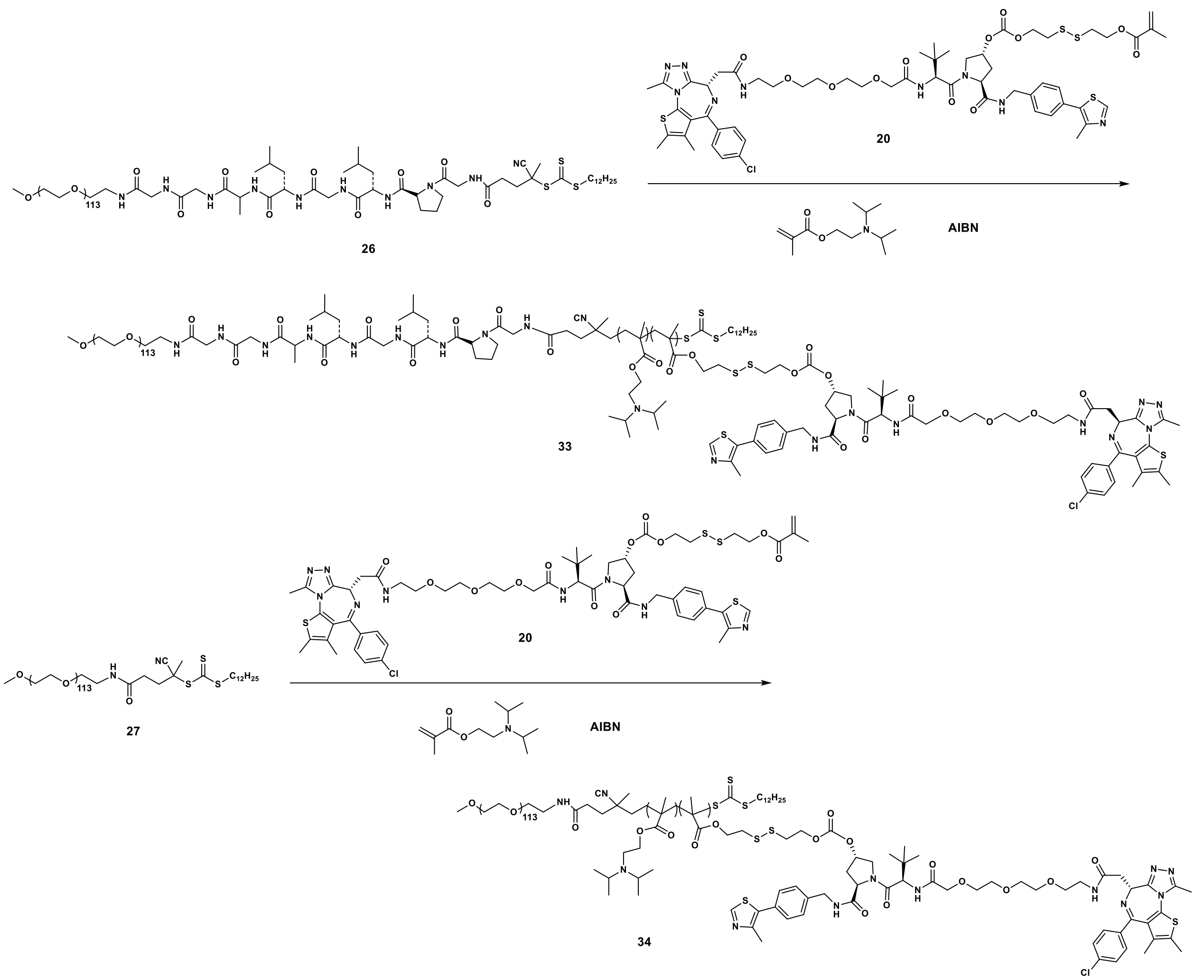

To synthesize POLY-PROTACs of MZ1, mPEG$_{113}$-CTA (200.0 mg, 0.037 mmol) or mPEG$_{113}$-GALGLPG-CTA (200.0 mg, 0.033 mmol), DPA (466.2 mg, 2.2 mmol), Me-MZ1 (189.0 mg, 0.15 mmol) and AIBN (0.54 mg, 0.0037 mmol) were dissolved in 1.5 mL of anhydrous DMF and stirred for 24 h under 70 °C. The final product was gained via dialyzing against DI water and lyophilization. The chemical structure and molecular weights of the resultant products were examined by ^{1}H-NMR spectra and GPC, respectively.

Synthesis of mPEG$_{113}$-*b*-P(DPA$_m$-*r*-HEMA$_n$) and mPEG$_{113}$-GALGLPG-*b*-P(DPA$_m$-*r*-HEMA$_n$)

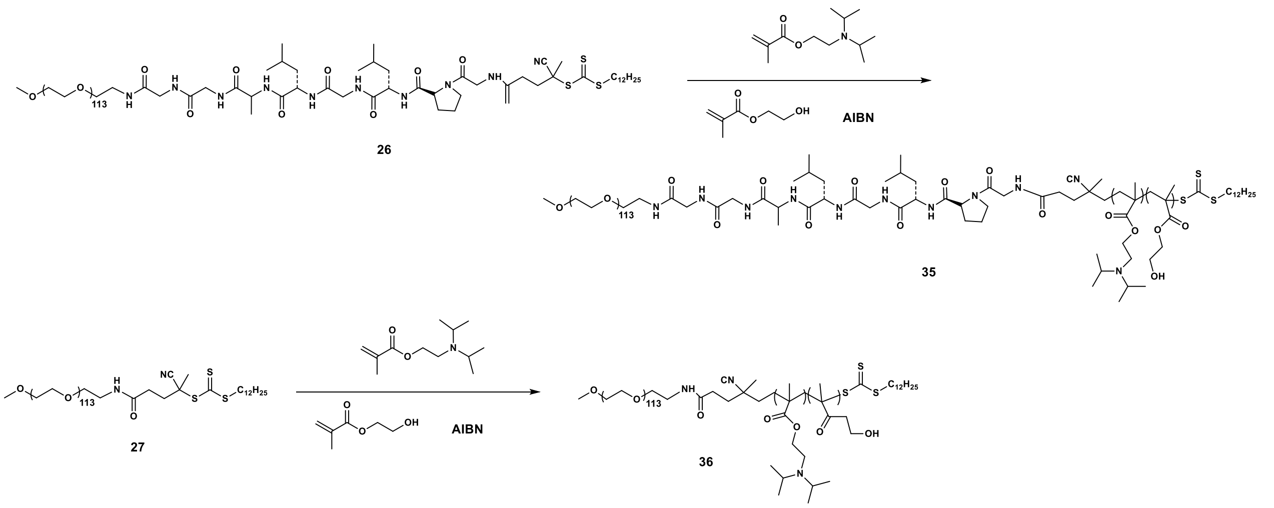

mPEG$_{113}$-CTA (200.0 mg, 0.037 mmol) or mPEG$_{113}$-GALGLPG-CTA (200.0 mg, 0.033 mmol), DPA (466.2 mg, 2.2 mmol), HEMA (48.10 mg, 0.37 mmol) and AIBN (0.54 mg, 0.0037 mmol) were dissolved in 1.5 mL of anhydrous DMF. The RAFT polymerization was sustained 24 h under 70 °C. The product was obtained through dialyzing against DI water and lyophilization. The chemical structure and molecular weights of the resultant products were examined by ^{1}H-NMR spectra and GPC, respectively.

Synthesis of mPEG$_{113}$-P(DPA $_m$-*r*-PPa$_n$) and mPEG$_{113}$-GALGLPG- P(DPA $_m$-*r*-PPa$_n$)

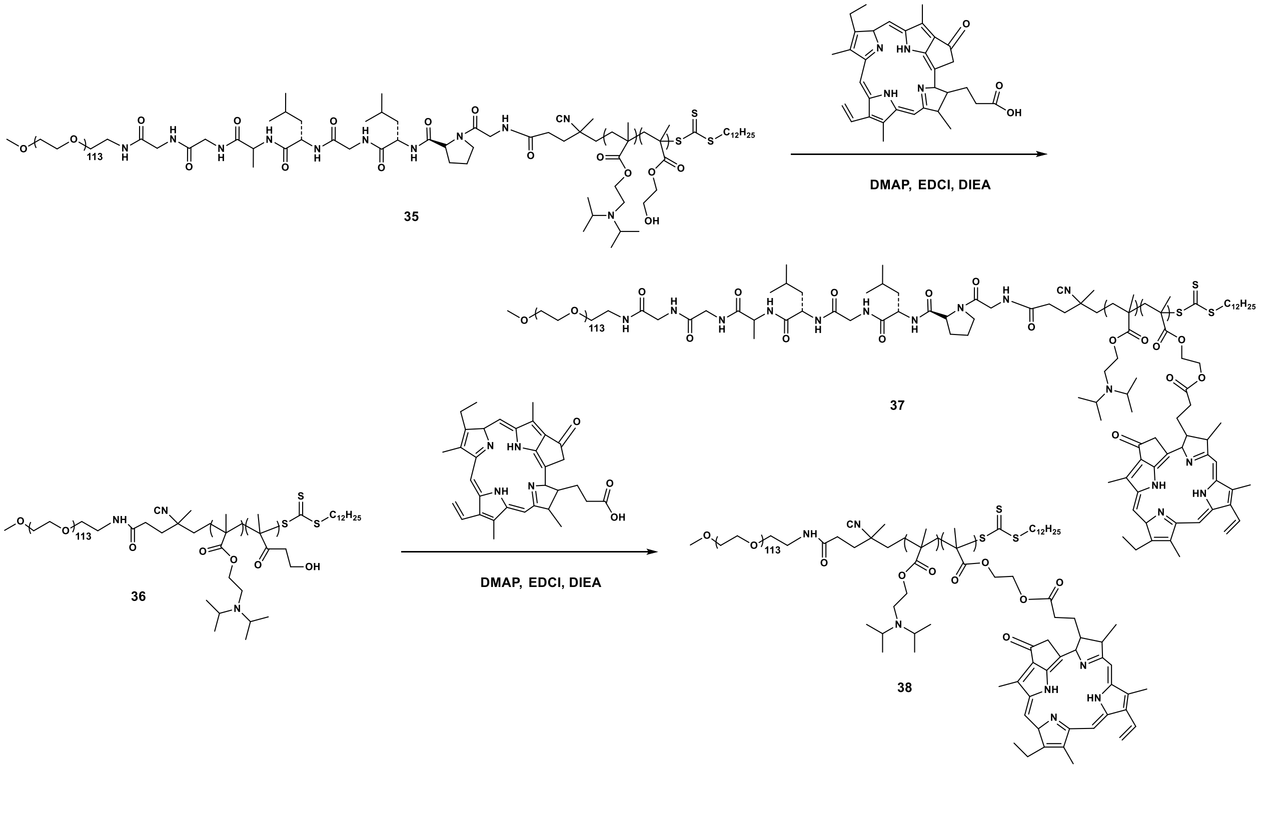

Briefly, PPa (93.24 mg, 0.175 mM), DMAP (42.95 mg, 0.35 mM), EDCI (67.10 mg, 0.35 mM), and DIEA (45.27 mg, 0.35 mM) were dissolved in anhydrous DMF and the reaction was continued for 90 min. Afterwards, mPEG$_{113}$-*b*-P(DPA$_m$-*r*-HEMA$_n$) (200.0 mg, 0.012 mmol) or mPEG$_{113}$-GALGLPG-*b*-P(DPA$_m$-*r*-HEMA$_n$) (201.4 mg, 0.012 mmol) dissolved in anhydrous DMF was added dropwise into above mixture, and stirred for 24 h at RT. The reaction solution was then dialyzed against DMSO and water, and lyophilized to obtain PPa-labled diblock copolymers. The chemical structure and molecular weights of the resultant products were examined by ^{1}H-NMR spectra and GPC, respectively.

Synthesis of GSH-insensitive POLY-PROTAC of mPEG$_{113}$-GALGLPG-P(DPA$_m$-*r*-O-ARV771$_n$)

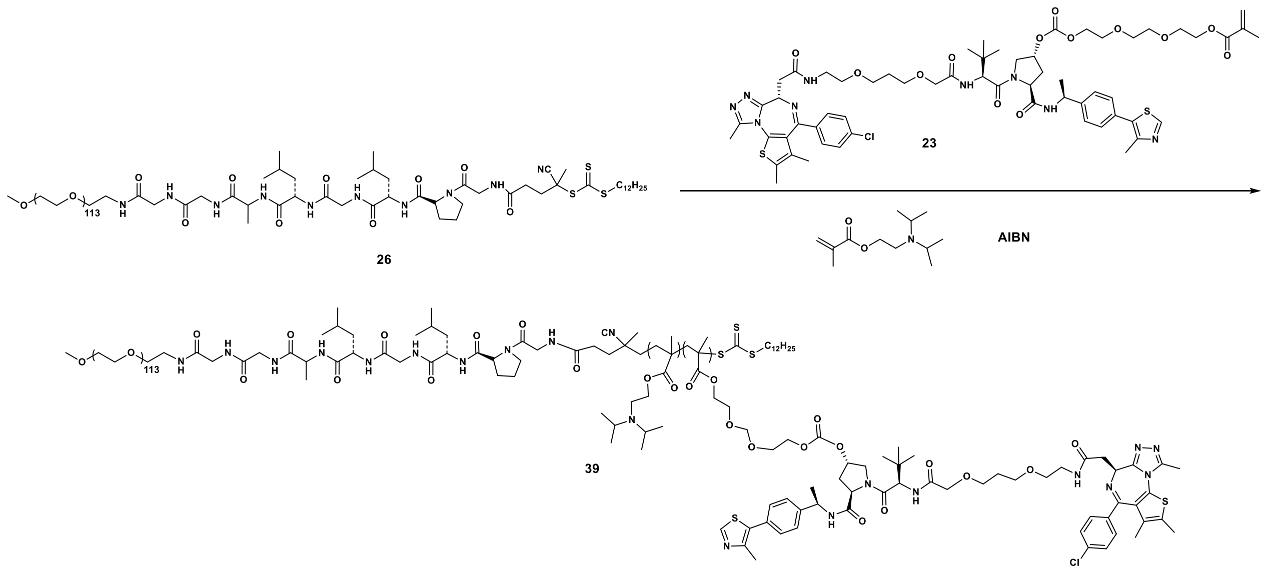

The mPEG$_{113}$-GALGLPG-CTA (200.0 mg, 0.033 mmol), DPA (466.2 mg, 2.2 mmol), Me-O-ARV771 (179.8 mg, 0.15 mmol) and AIBN (0.54 mg, 0.0037 mmol) were dissolved in 1.5 mL of anhydrous DMF. Undergoing stirred persistently for 24 h at 70°C, the reaction solution was purified via dialyzing against DI water and lyophilized to obtain the final product. The chemical structure and molecular weights of the resultant products were examined by ^{1}H-NMR spectra and GPC, respectively.

Synthesis of $mPEG_{113}$-P(EPA_m-*r*-$DBCO_n$) and $mPEG_{113}$-P(EPA_m-*r*-PPa_n)

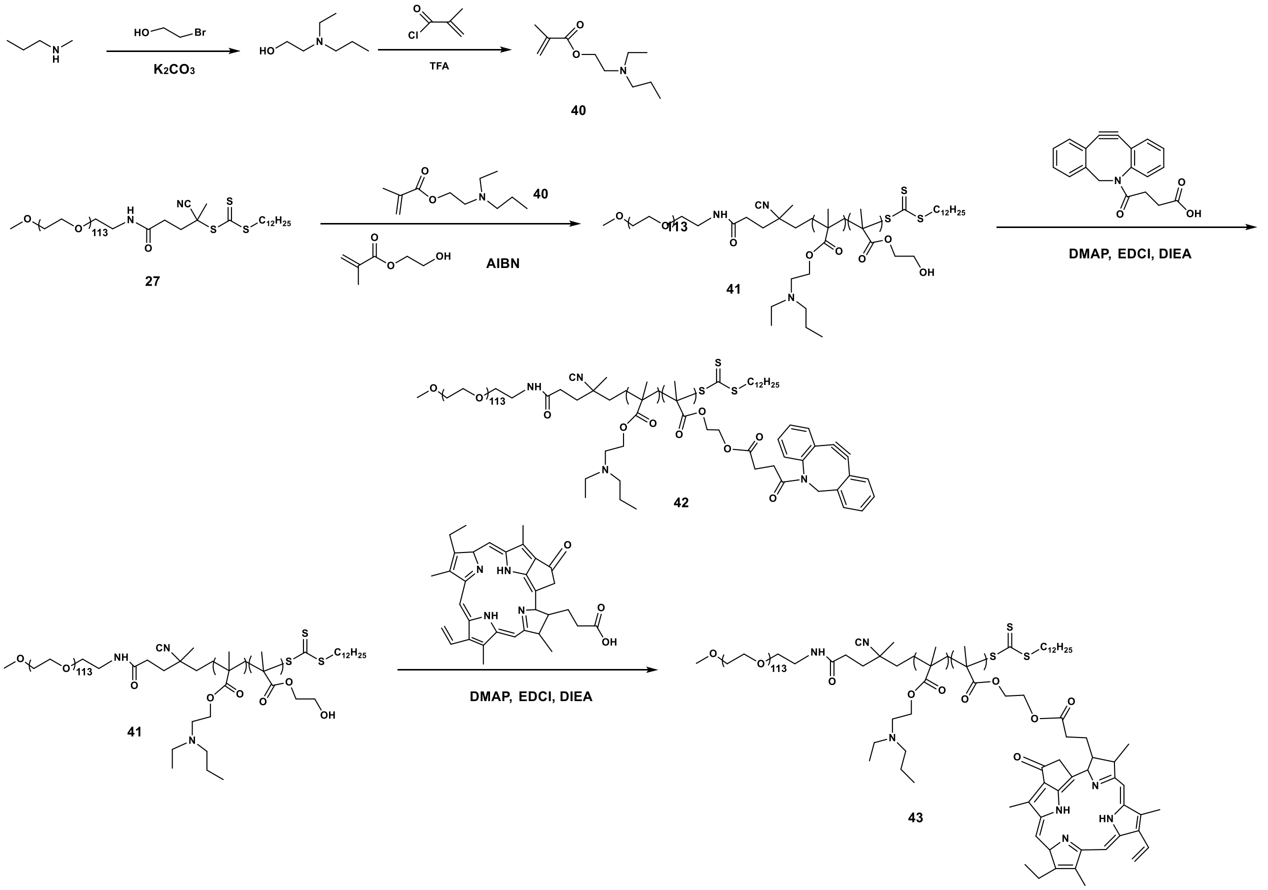

To synthesize ethylpropylaminoethyl (EPA) methacrylate, N-ethylpropan-1-amine (3.5 g, 0.04 mol), 2-bromoethanol (5.0 g, 0.04 mol) and potassium carbonate (16.6 g, 0.12 mol) were dissolved in acetonitrile, the mixture solution was stirred overnight at RT. After filtered to remove the potassium carbonate, the reaction mixture was condensed by vacuum evaporation. Secondly, the above crude product was dissolved in anhydrous DCM, methacryloyl chloride (5.2 g, 0.05 mol) in anhydrous DCM was added dropwise under constant pressure and ice bath. After the reaction was finished, the triethylamine-HCl salts and solvent was removed by filtration and vacuum evaporation. The final product was obtained by vacuum distillation as colorless liquid.

To prepare $mPEG_{113}$-*b*-P(EPA_m-*r*-$HEMA_n$) diblock copolymer, $mPEG_{113}$-CTA (200.0 mg, 0.037 mmol), EPA methacrylate (517.4 mg, 2.6 mmol), HEMA (48.10 mg, 0.37 mmol) and AIBN (0.54 mg, 0.0037 mmol) were dissolved in 1.5 mL of anhydrous DMF. The RAFT polymerization was continued for 24 h under 70 °C. Then, the final product was purified by dialyzing against DI water and freeze dried.

To prepare mPEG$_{113}$-P(EPA$_m$-*r*-DBCO$_n$) and mPEG$_{113}$-P(EPA$_m$-r-PPa$_n$), DBCO-acid (50.47 mg, 0.165 mM) or PPa (29.37 mg, 0.055mM), DMAP (8.060 mg, 0.33 mM), EDCI (12.65 mg, 0.33 mM), and DIEA (8.8 mg, 0.33 mM) were dissolved in anhydrous DMF. After stirring for 90 min, the DMF solution of mPEG$_{113}$-*b*-P(EPA$_m$-*r*-HEMA$_n$) (200.0 mg, 0.011 mmol) was added dropwise into the mixture, and stirred continued for 24 h at RT. Then, the product was obtained by dialyzing against DMSO/DI water, and lyophilization. The chemical structure of the intermediates and final products was examined by ^{1}H-NMR spectra. The molecular weights of the resultant diblock copolymers were determined by GPC measurement.

Synthesis of N$_3$-PEG$_{113}$-GALGLPG-*b*-P(DPA$_m$-*r*-HEMA$_n$)

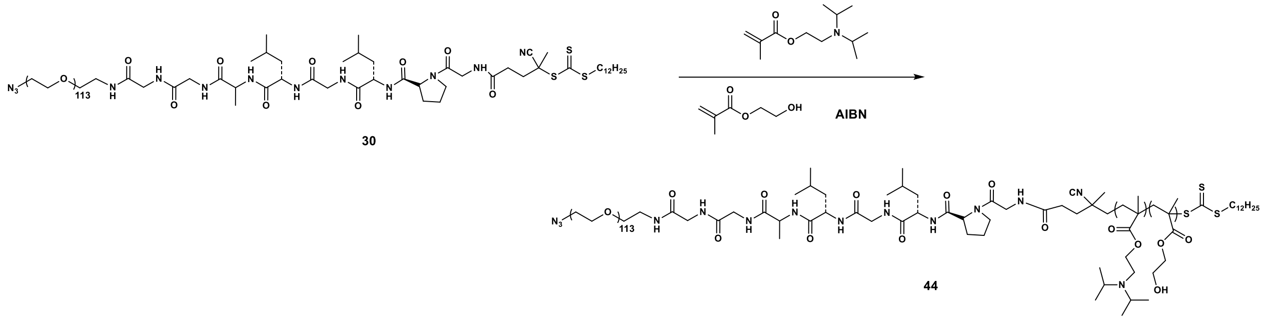

N$_3$-PEG$_{113}$-GALGLPG-CTA (200.0 mg, 0.033 mmol), DPA (466.2 mg, 2.2 mmol), HEMA (48.1 mg, 0.37 mmol) and AIBN (0.54 mg, 0.0037 mmol) were dissolved in 1.5 mL of anhydrous DMF. The RAFT polymerization was continued for 24 h at 70 °C, and the final product was purified by dialyzing against DI water and lyophilizing. The chemical structure and molecular weights of the resultant diblock copolymer were determined by ^{1}H-NMR spectra and GPC measurement, respectively.

Supplementary Table 1. ^{1}H-NMR spectrum and GPC-determined molecular weights of the diblock copolymers and POLY-PROTACs synthesized in this study.

Copolymer	^{1}H-NMR	GPC (Da)		PDI
	M*n* (Da)	M*w* (Da)	M*n* (Da)	(M*w*/M*n*)
PGD7	17412	17776	13864	1.28
PD7	17649	17109	13549	1.26
PGDM	17444	17508	13487	1.29
PDM	16829	16249	12789	1.27
PGDH	16913	16598	13266	1.25
PGDA	19324	18756	13106	1.43
PGDO7	16966	15334	11724	1.30
N_3PGDH	15430	14669	11808	1.24
PED	19400	19243	15566	1.23

* GPC measurment was perfomed with a mobile phase of tetrahydrofuran (THF) at a flow rate of 1.0 mL/min and temperature of 35 ℃.

PGD7: mPEG$_{113}$-GALGLPG-*b*-P(DPA$_{42}$-*r*-ARV771$_{2}$);

PD7: mPEG$_{113}$-*b*-P(DPA$_{46}$-*r*-ARV771$_{2}$);

PGDM: mPEG$_{113}$-GALGLPG-*b*-P(DPA$_{45}$-*r*-MZ1$_{2}$);

PDM: mPEG$_{113}$-*b*-P(DPA$_{42}$-*r*-MZ1$_{2}$);

PGDH: mPEG$_{113}$-GALGLPG-*b*-P(DPA$_{48}$-*r*-HEMA$_{7}$);

PGDA: mPEG$_{113}$-GALGLPG-*b*-P(DPA$_{48}$-*r*-HEMA$_{7}$-PPa$_{4}$);

PGDO7: mPEG$_{113}$-GALGLPG-*b*-P(DPA$_{40}$-*r*- oARV771$_{2}$);

N_3PGDH: N_3-PEG$_{113}$-GALGLPG-*b*-P(DPA$_{41}$-*r*-HEMA$_{7}$);

PED: mPEG$_{113}$-*b*-P(EPA$_{60}$-*r*-HEMA$_{6}$-DBCO$_{4}$).

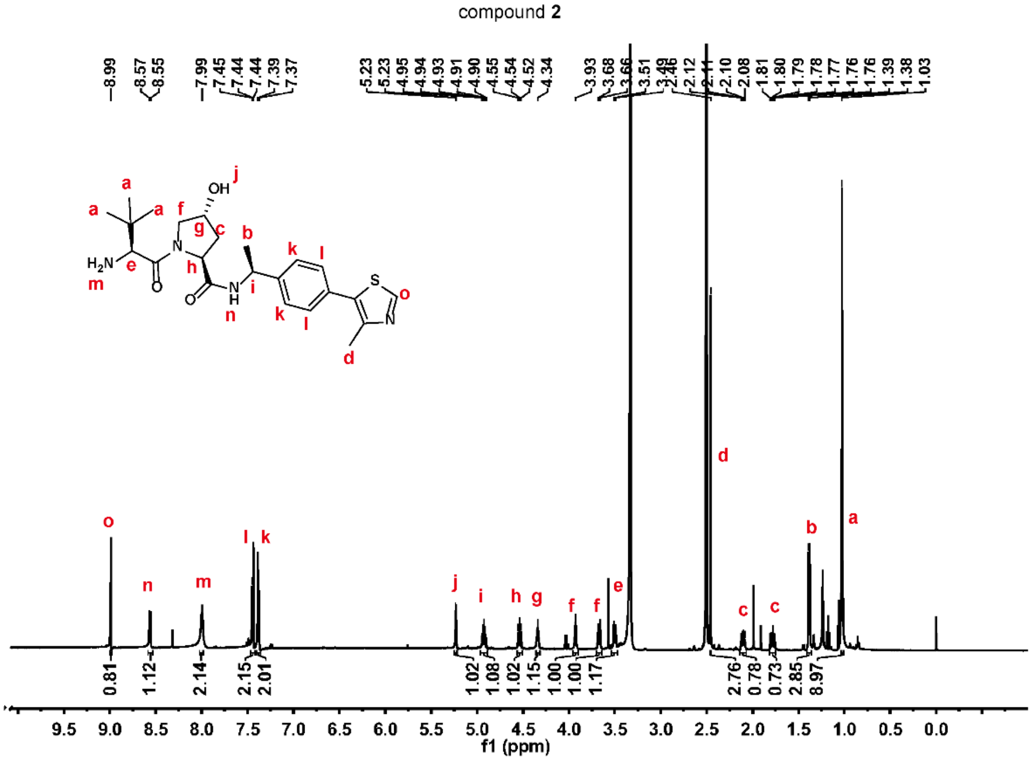

Supplementary Figure 1. ^{1}H-NMR spectrum of compound **2** ($CDCl_3$).

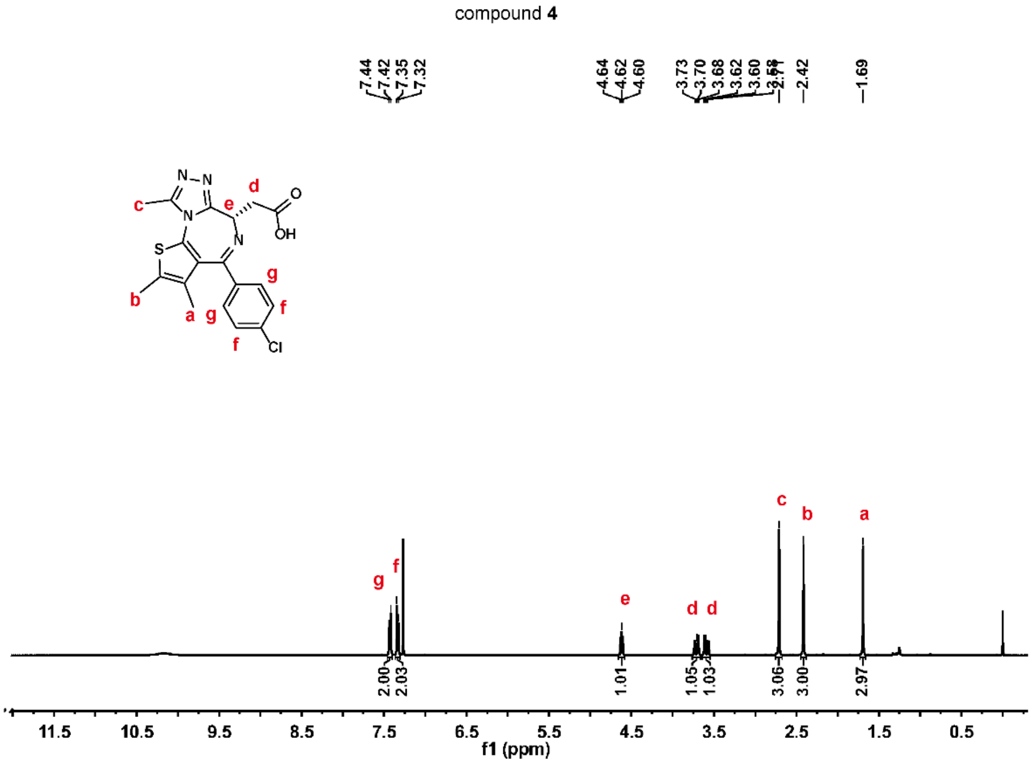

Supplementary Figure 2. ^{1}H-NMR spectrum of compound **4** ($CDCl_3$).

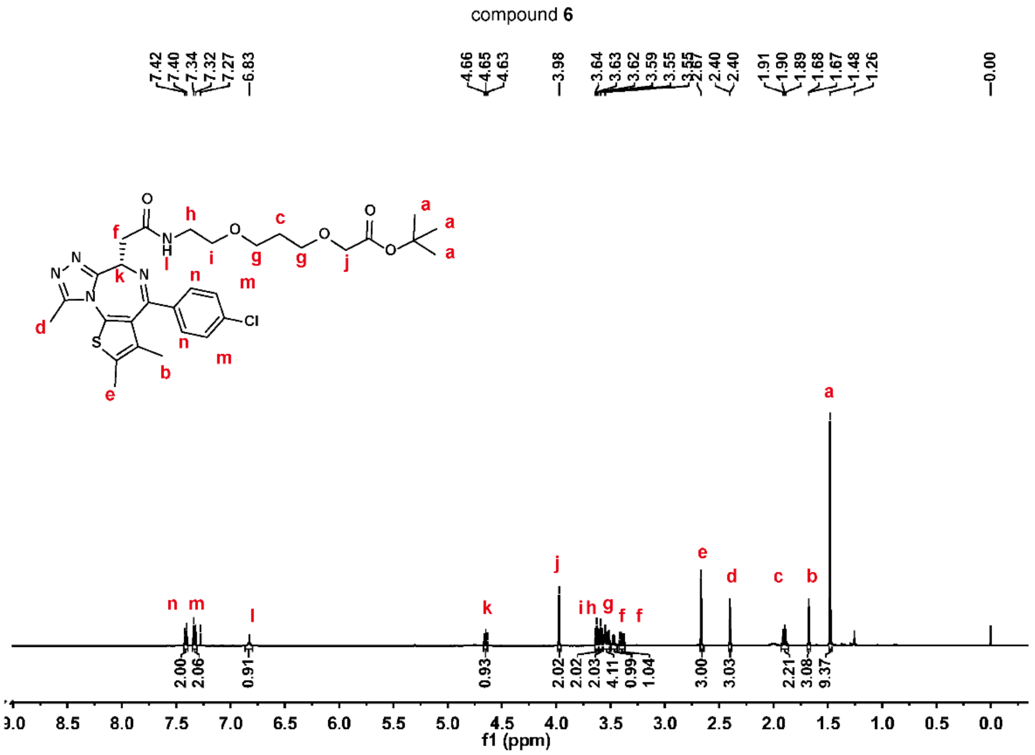

Supplementary Figure 3. ^{1}H-NMR spectrum of compound **6** ($CDCl_3$).

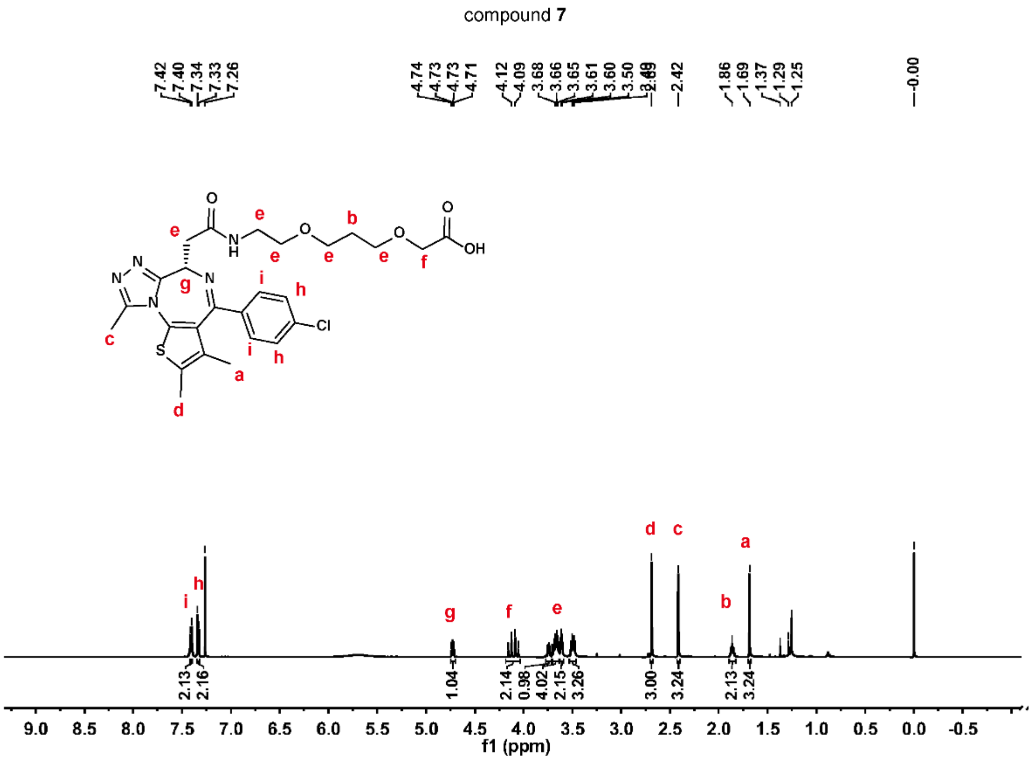

Supplementary Figure 4. ^{1}H-NMR spectrum of compound **7** ($CDCl_3$).

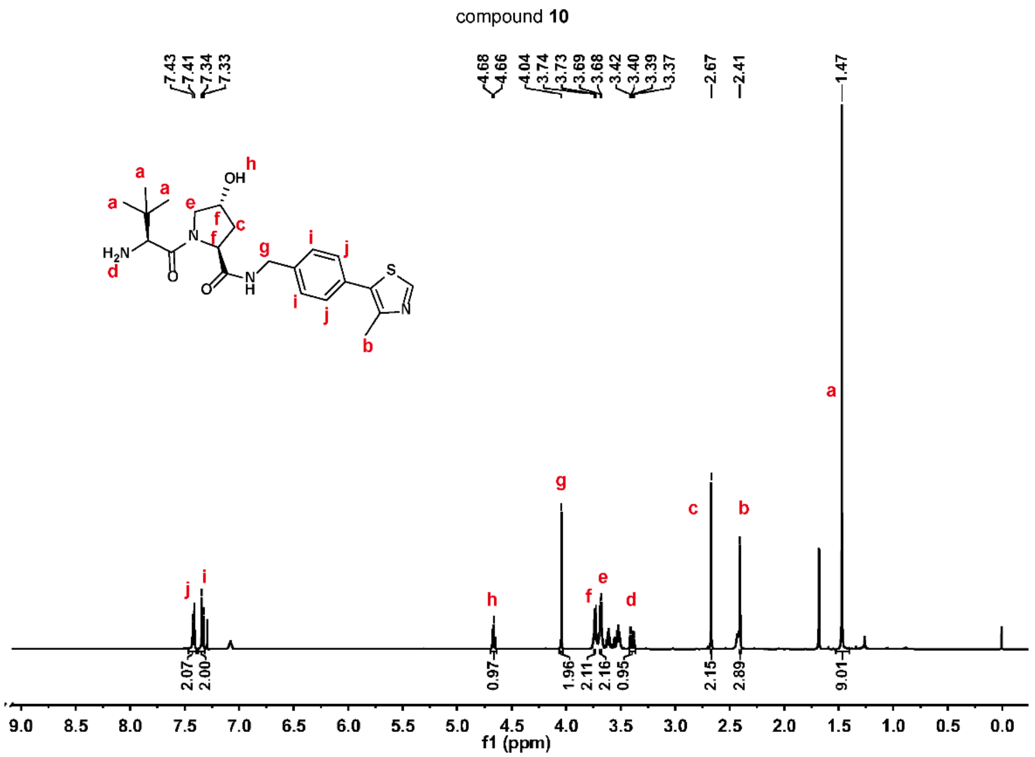

Supplementary Figure 5. ^{1}H-NMR spectrum of compound **10** ($CDCl_3$).

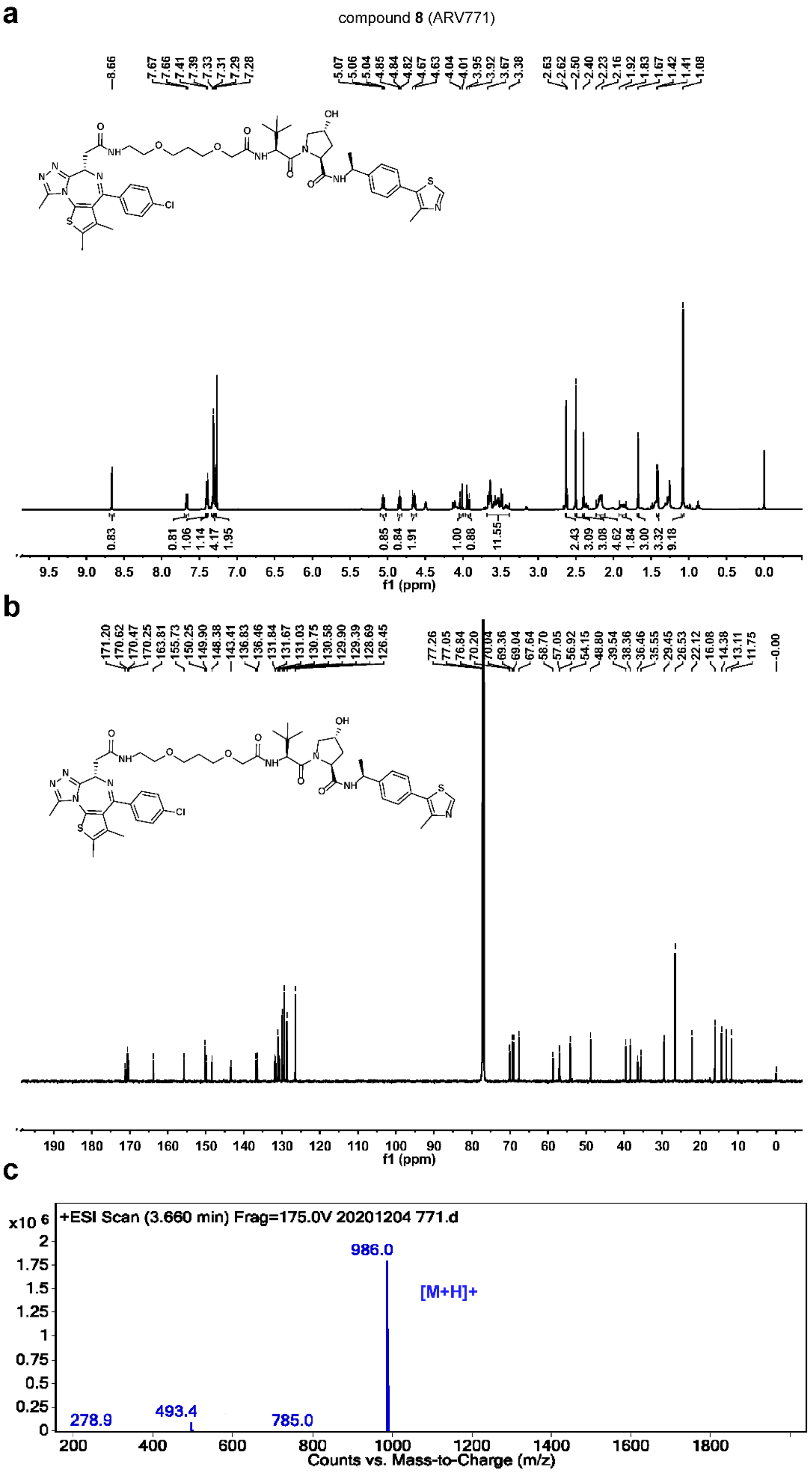

Supplementary Figure 6. a. ^{1}H-NMR spectrum, **b.** ^{13}C-NMR spectrum ($CDCl_3$), and **c**. mass spectrum of ARV771.

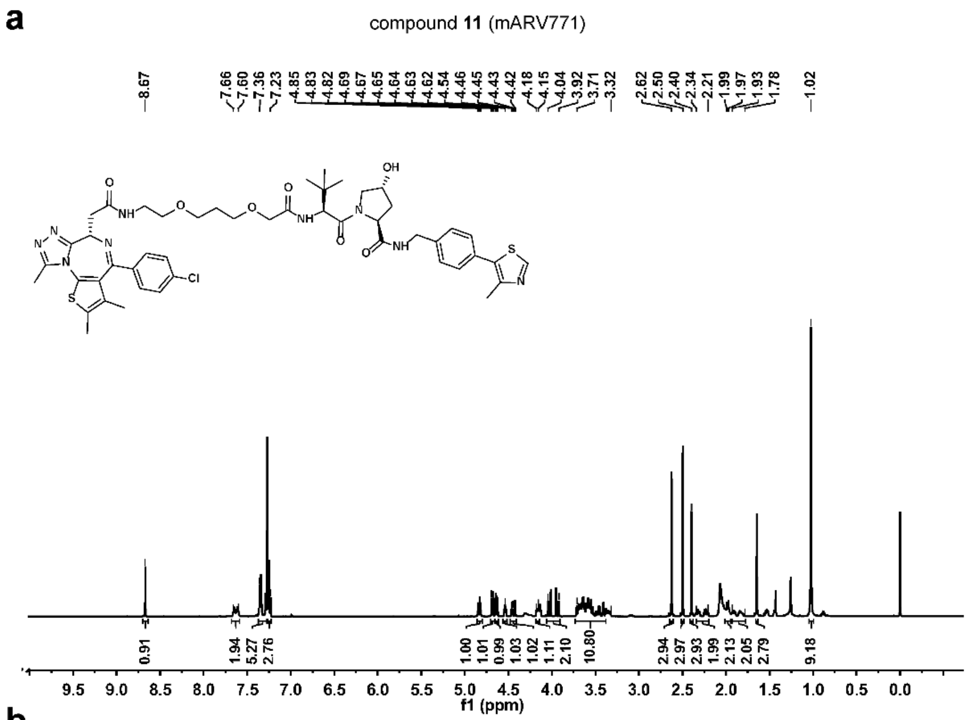

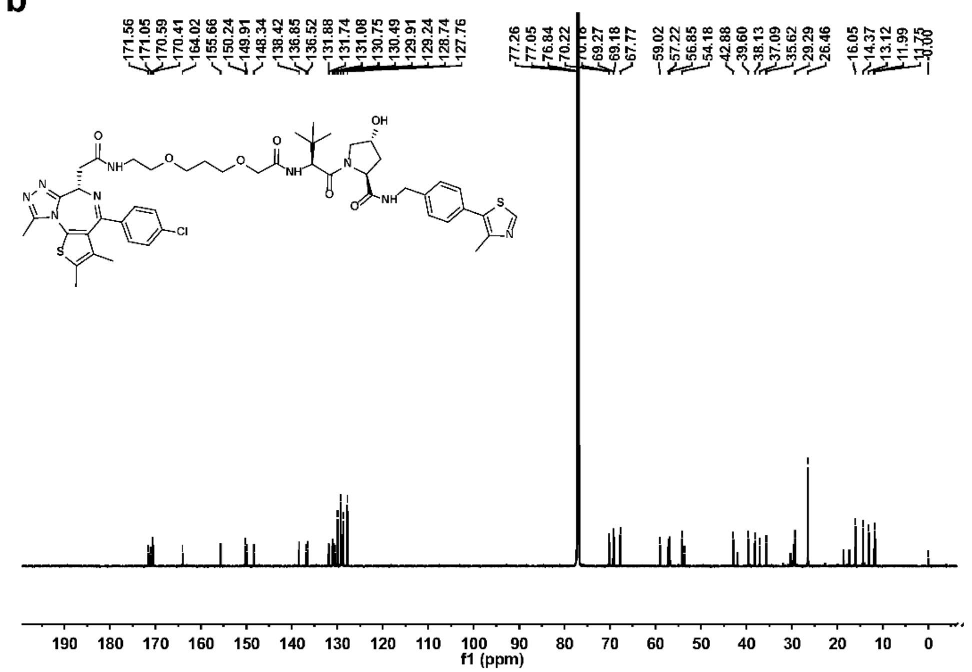

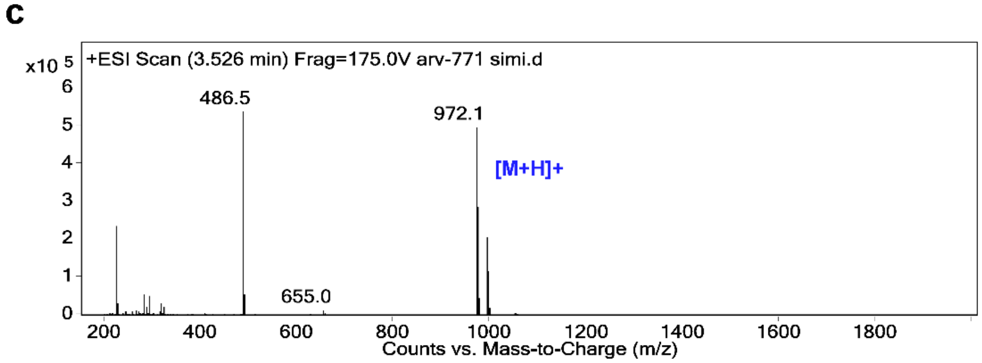

Supplementary Figure 7. a. ^{1}H-NMR spectrum ($CDCl_3$), **b.** ^{13}C-NMR spectrum, and **c**. mass spectrum of mARV771.

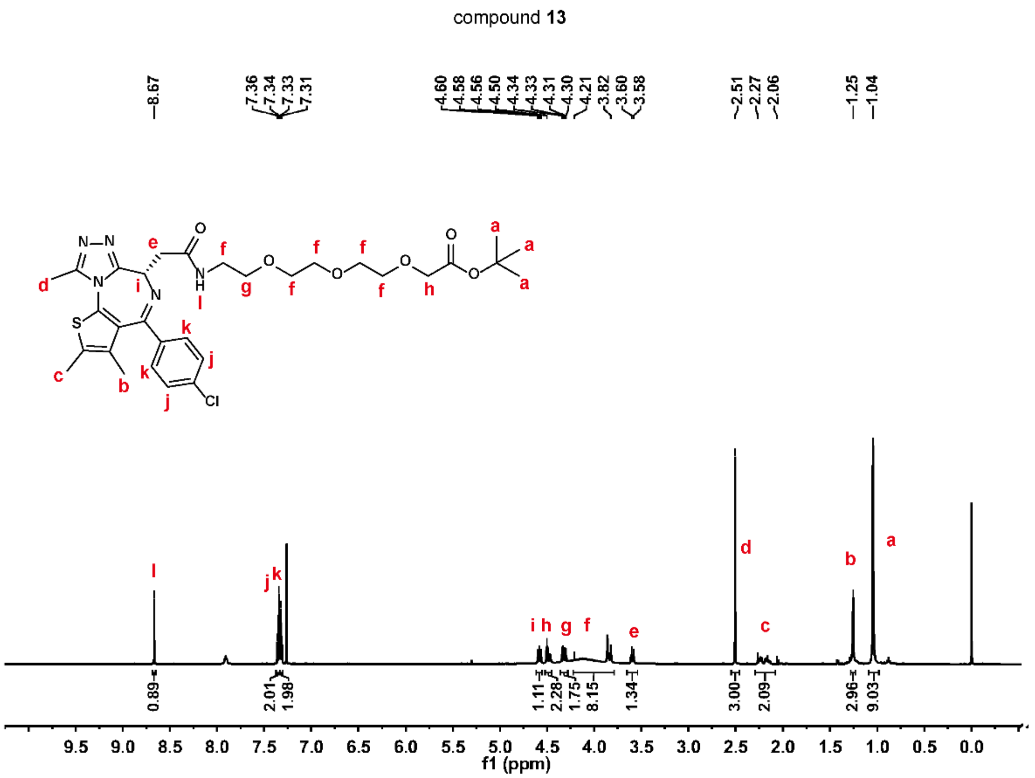

Supplementary Figure 8. ^{1}H-NMR spectrum of compound **13** ($CDCl_3$).

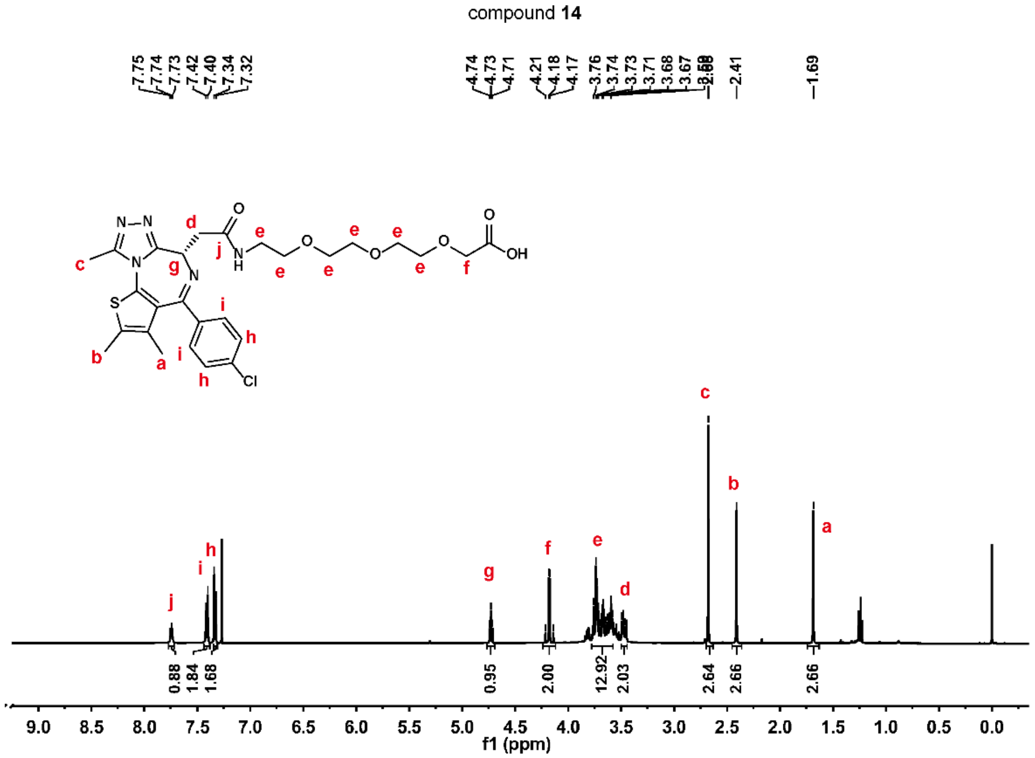

Supplementary Figure 9. ^{1}H-NMR spectrum of compound **14** ($CDCl_3$).

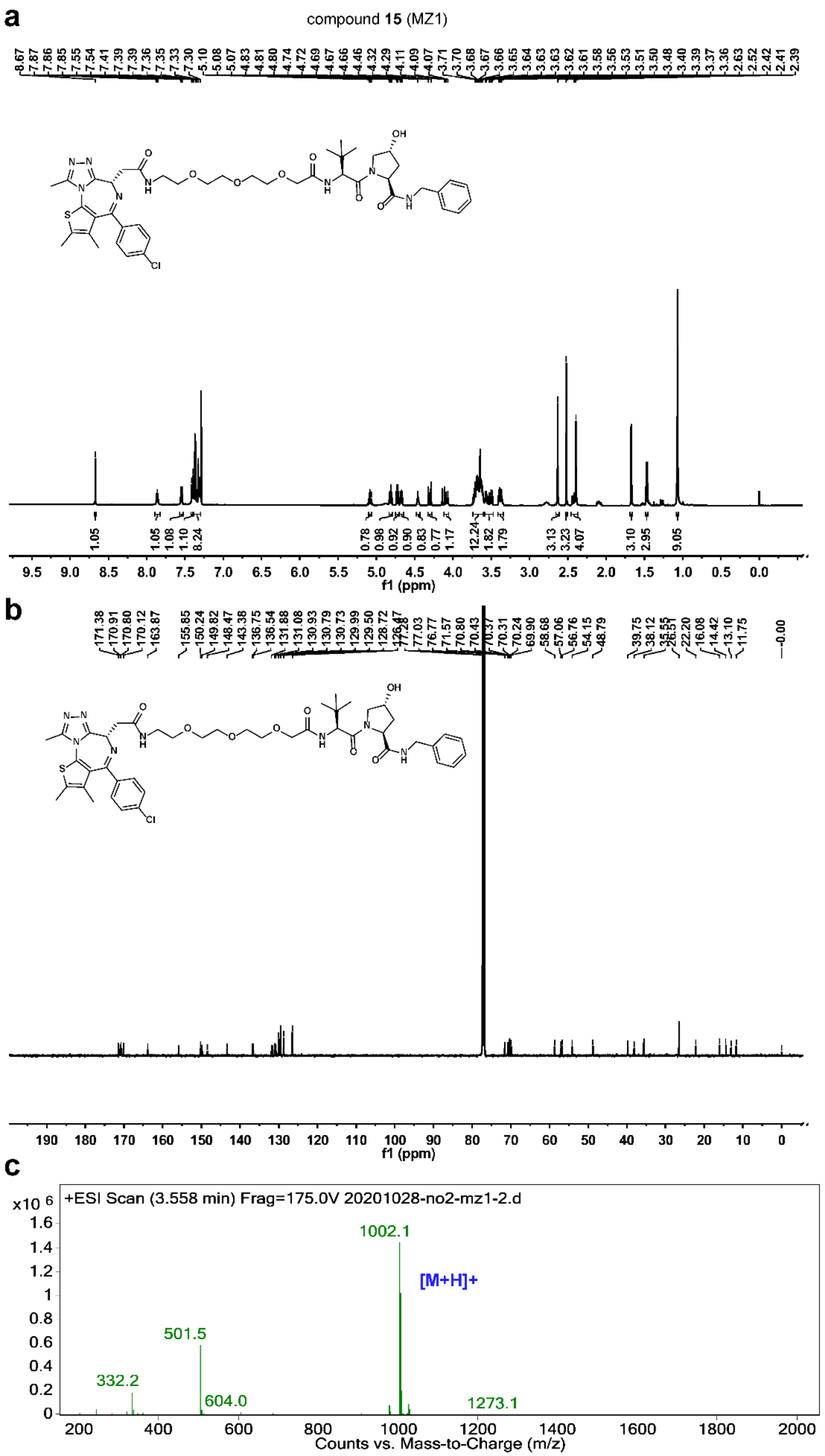

Supplementary Figure 10. a, ^{1}H-NMR spectrum ($CDCl_3$), **b,** ^{13}C-NMR spectrum ($CDCl_3$), and **c**. mass spectrum of MZ1.

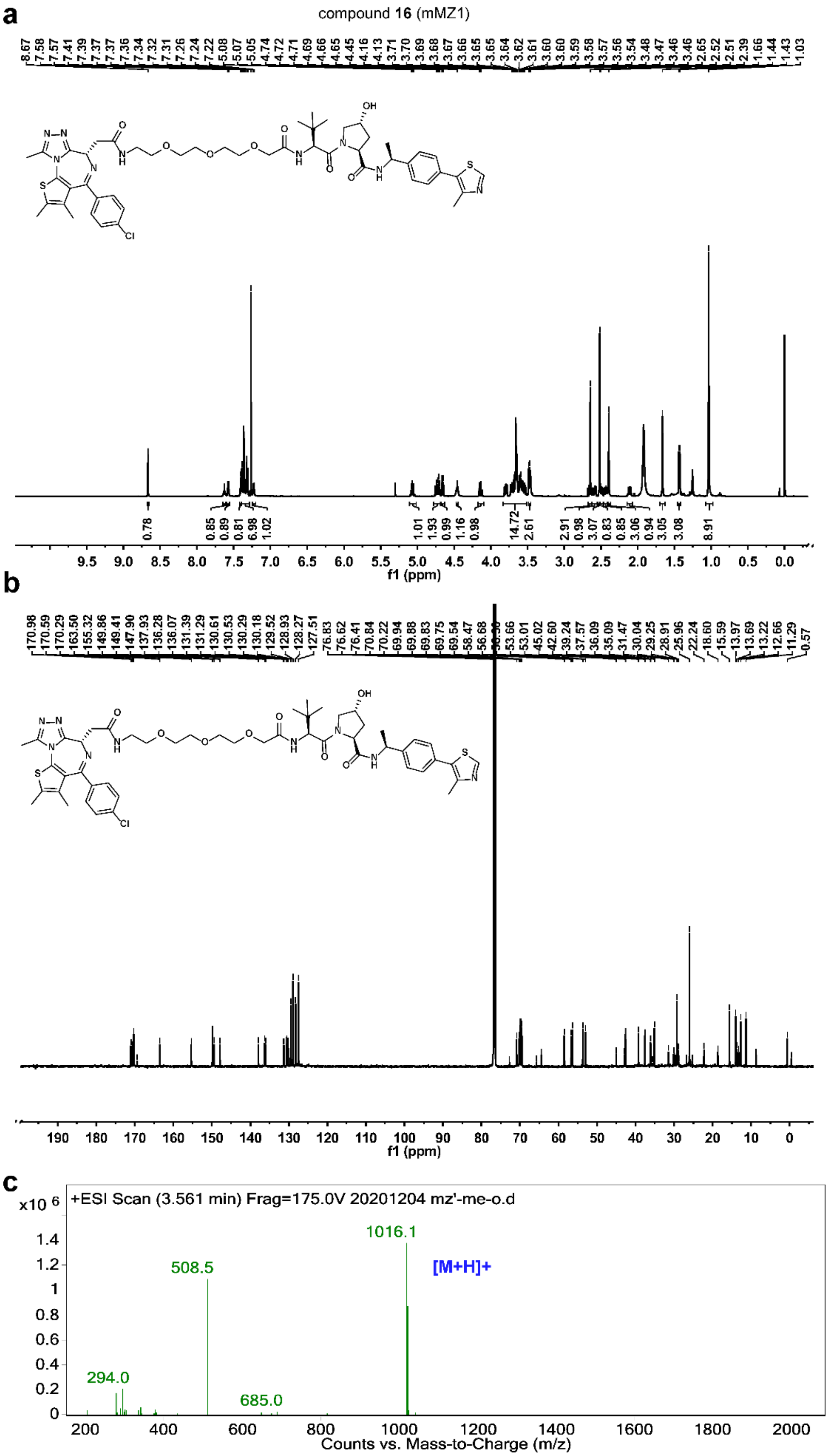

Supplementary Figure 11. a, ^{1}H-NMR spectrum ($CDCl_3$), **b,** ^{13}C-NMR spectrum ($CDCl_3$), and **c**. mass spectrum of mMZ1.

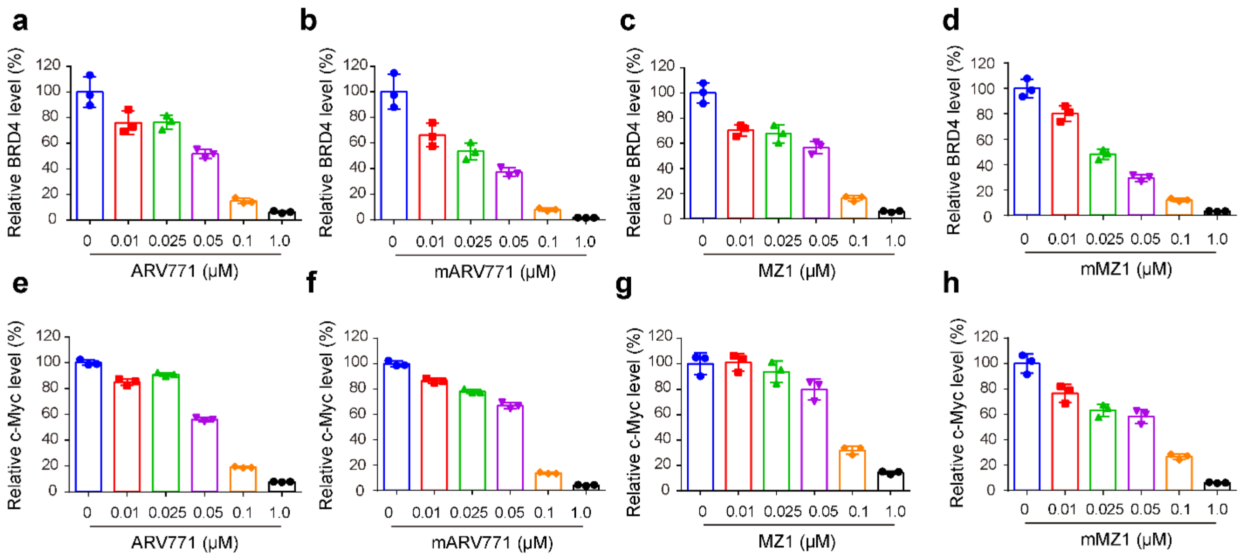

Supplementary Figure 12. a-d. Normalized BRD4 expression in MDA-MB-231 cells with the treatment of (**a**) ARV771, (**b**) mARV771, (**c**) MZ1 and (**d**) mMZ1. **e-h.** The normalized c-Myc level in MDA-MB-231 cell with the treatment of (**e**) ARV771, (**f**) mARV771, (**g**) MZ1, and (**h**) mMZ1 for 24 h. All data are presented as mean ± SD. (n = 3 biologically independent cells)

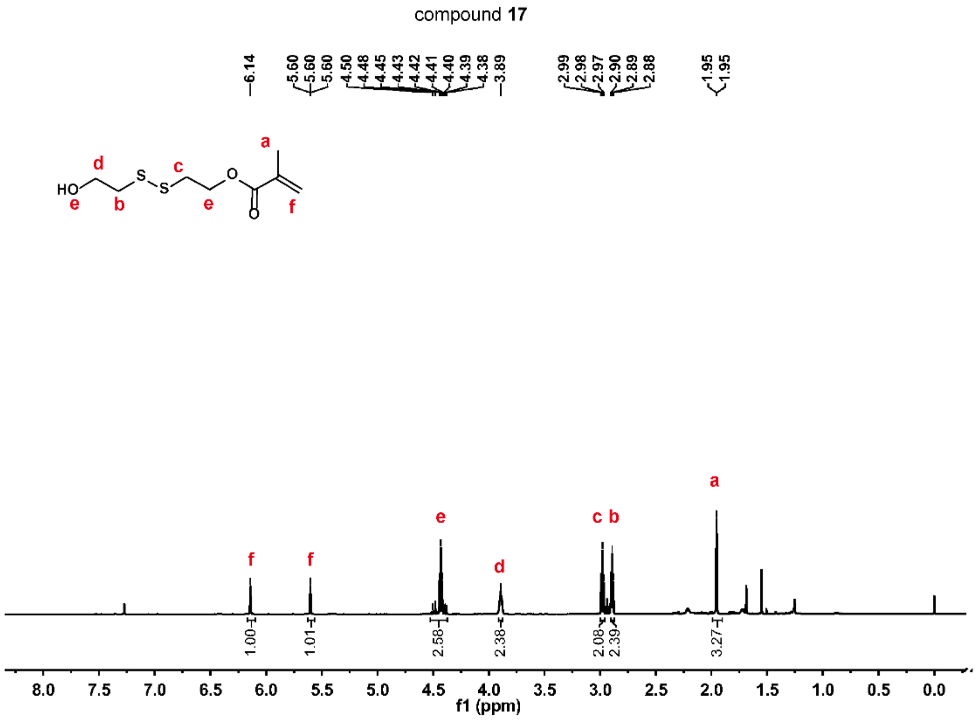

Supplementary Figure13. ^{1}H-NMR spectrum of compound **17** ($CDCl_3$).

a

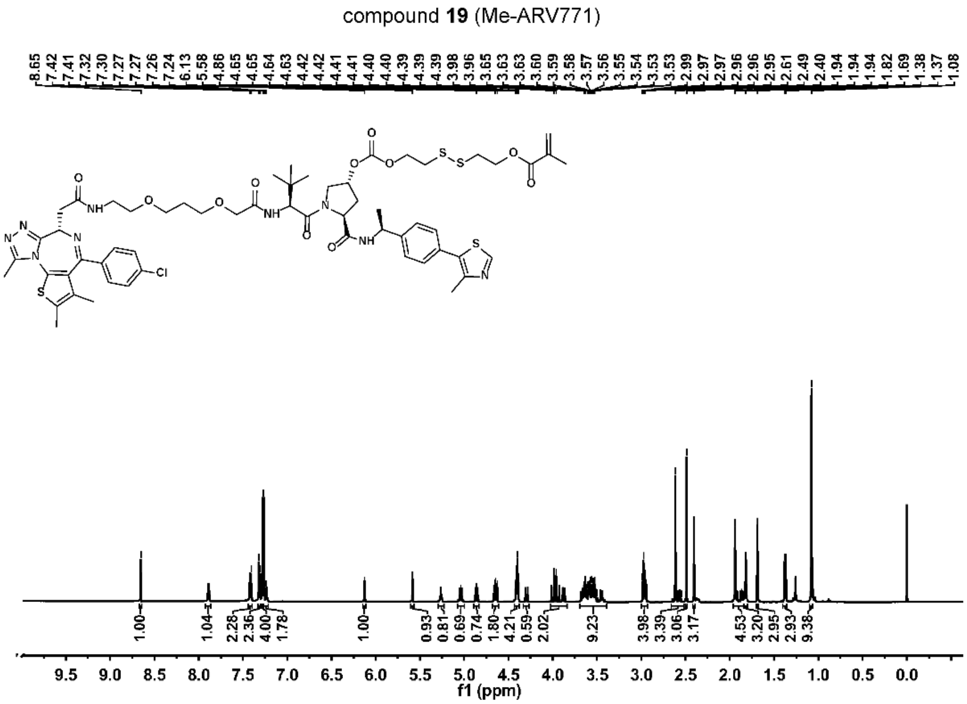

b

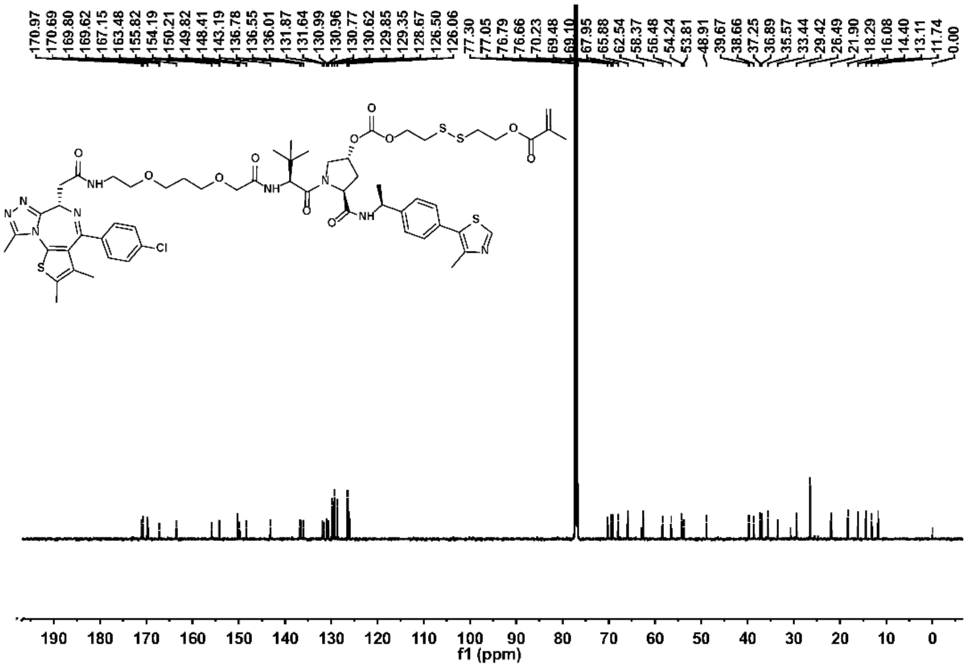

c

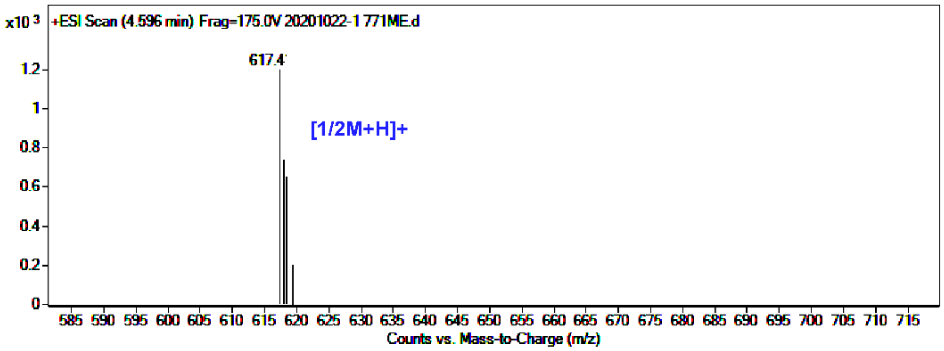

Supplementary Figure 14. a. ^{1}H-NMR spectrum ($CDCl_3$), **b.** ^{13}C-NMR spectrum ($CDCl_3$), and **c**. mass spectrum of Me-ARV771.

a

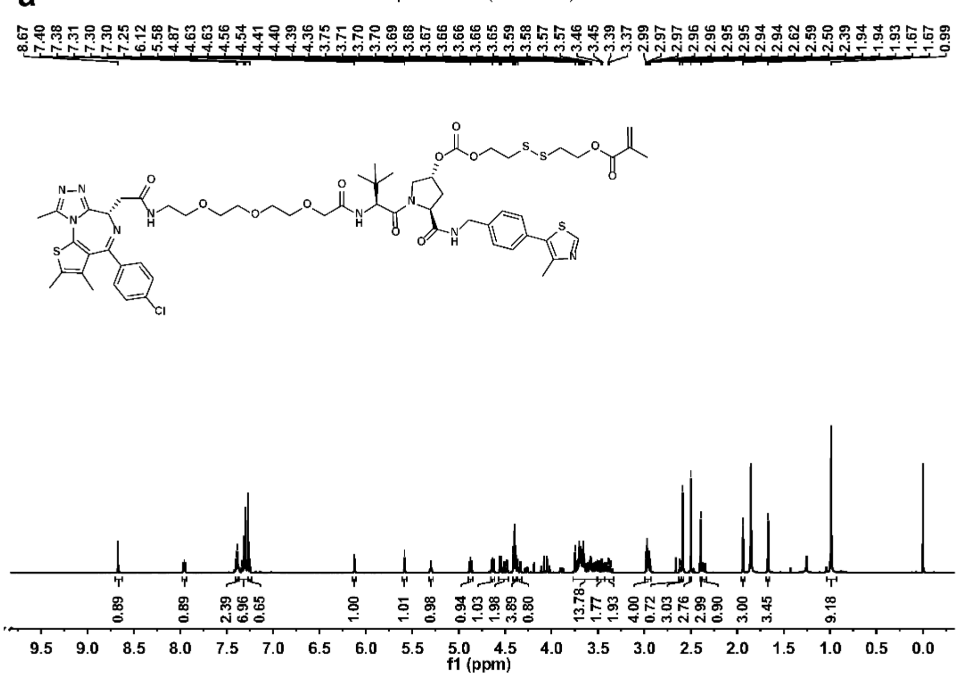

b

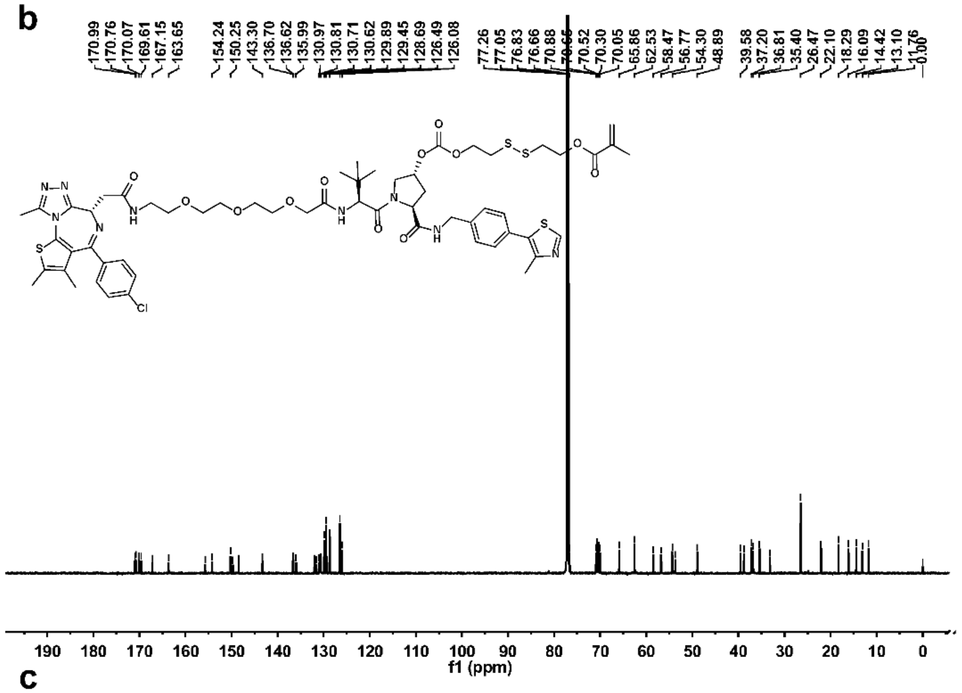

c

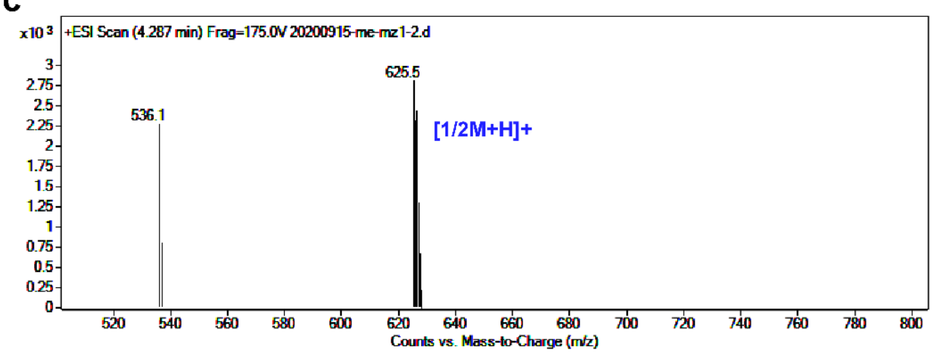

Supplementary Figure 15. a. ^{1}H-NMR spectrum ($CDCl_3$), **b.** ^{13}C-NMR spectrum ($CDCl_3$), and **c** mass spectrum of Me-MZ1.

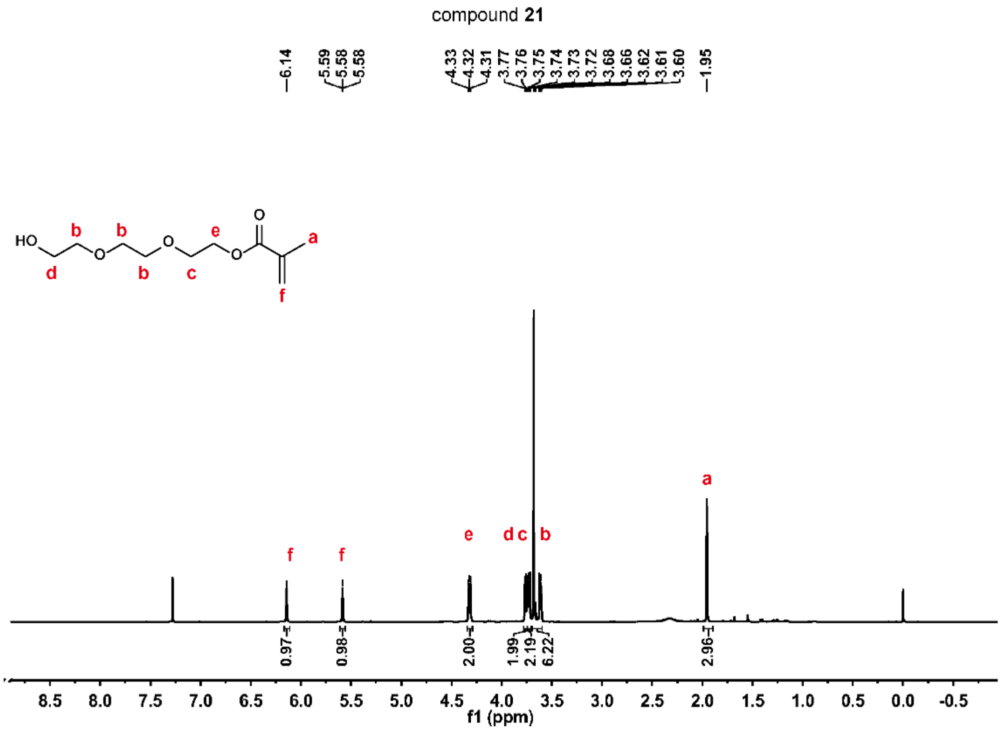

Supplementary Figure 16. ^{1}H-NMR spectrum of compound **21** ($CDCl_3$).

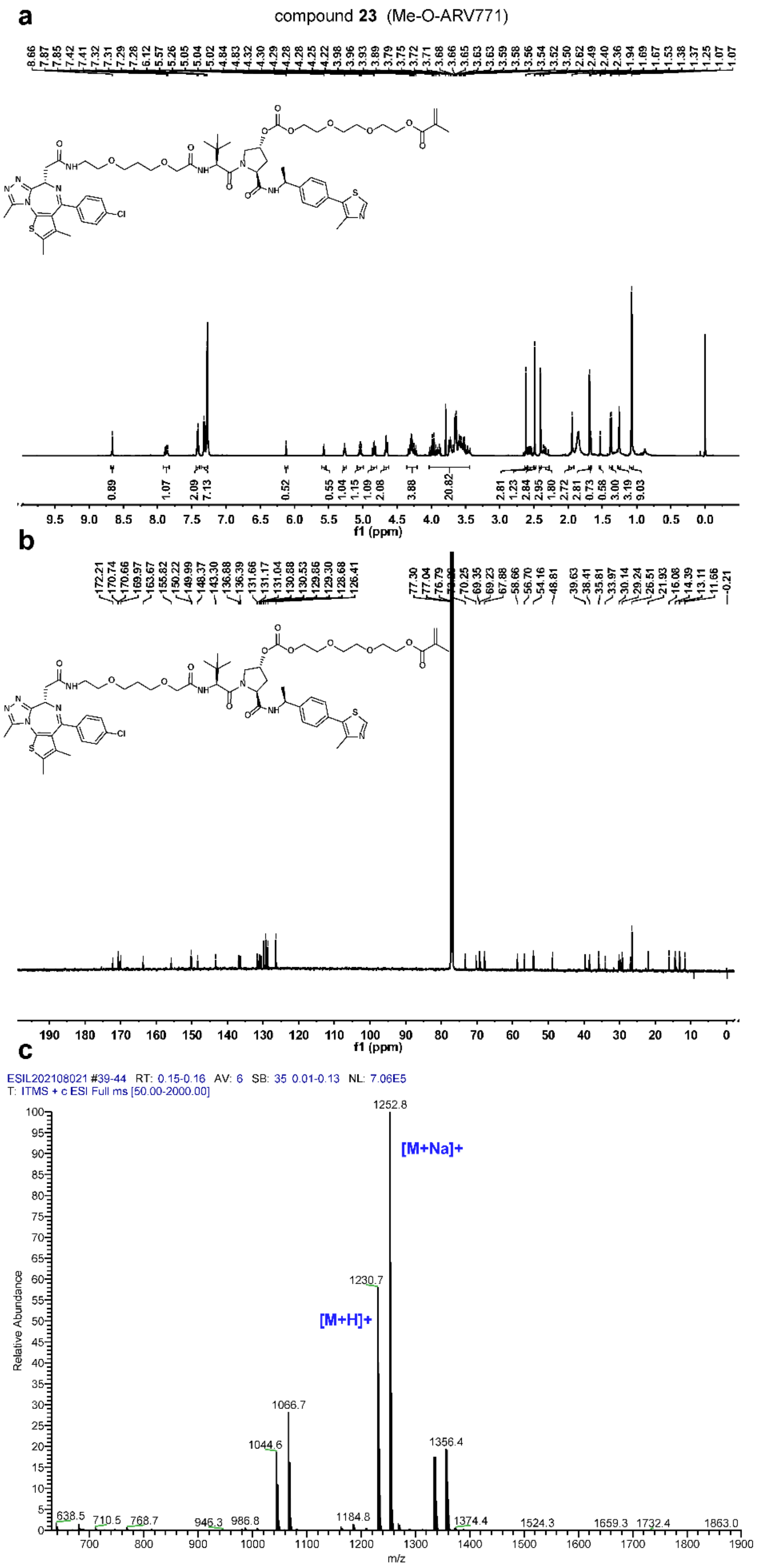

Supplementary Figure 17. a. ^{1}H-NMR spectrum ($CDCl_3$), **b**. ^{13}C-NMR spectrum ($CDCl_3$), and **c**. mass spectrum of Me-O-ARV771.

compound **24**

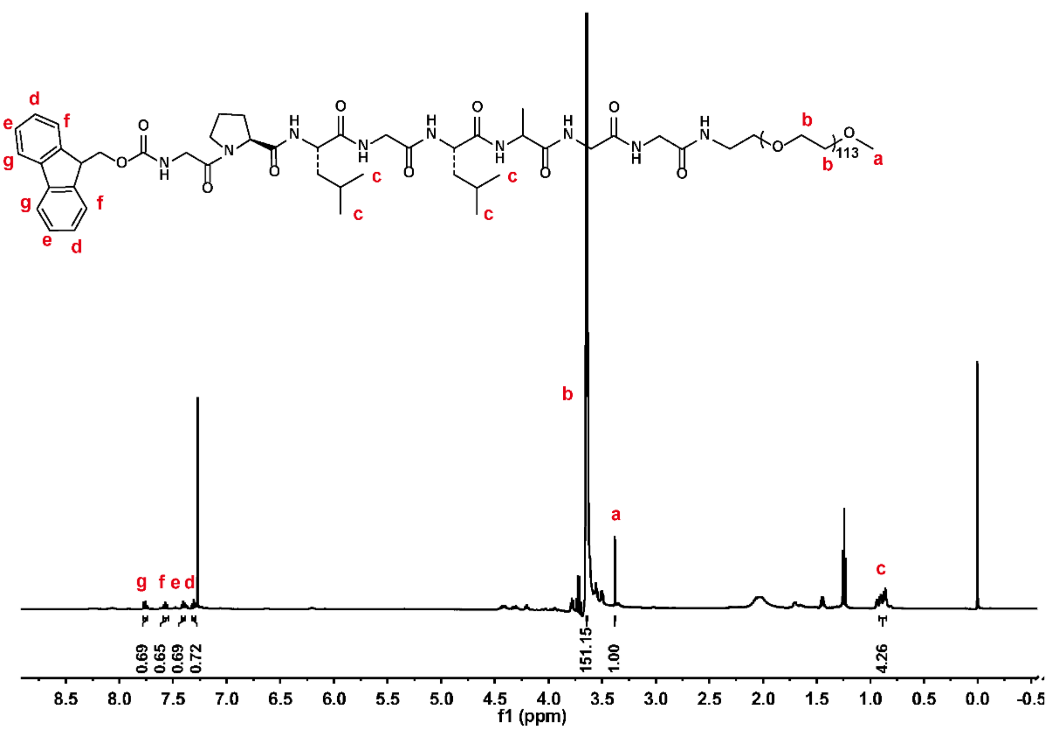

Supplementary Figure 18. ^{1}H-NMR spectrum of compound **24** ($CDCl_3$).

compound **25**

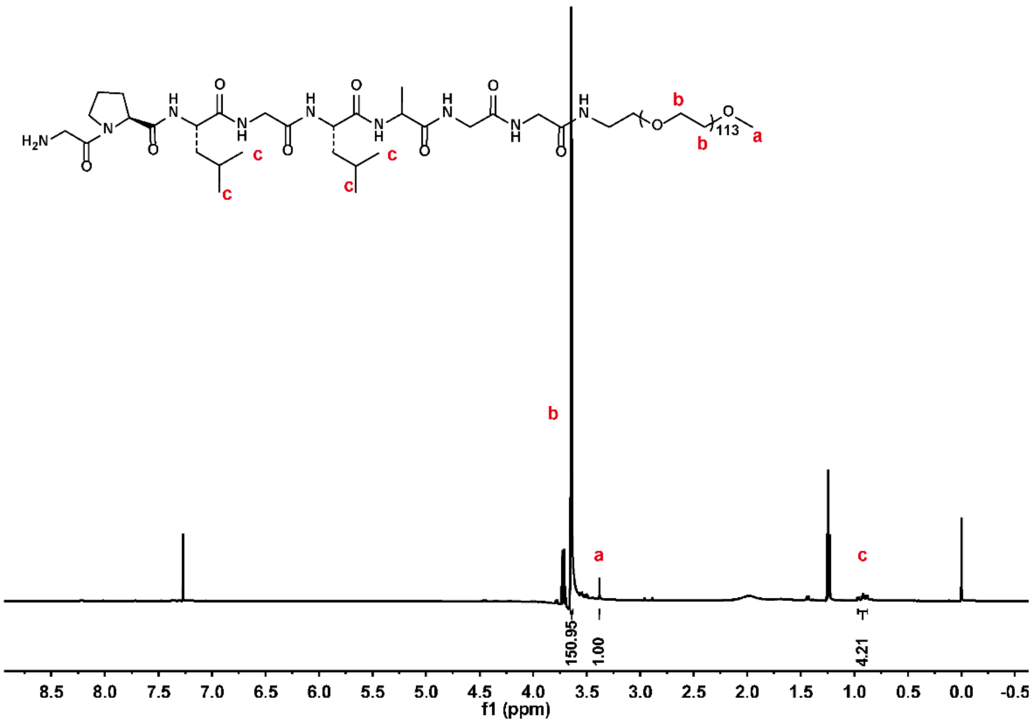

Supplementary Figure 19. ^{1}H-NMR spectrum of compound **25** ($CDCl_3$).

compound **26**

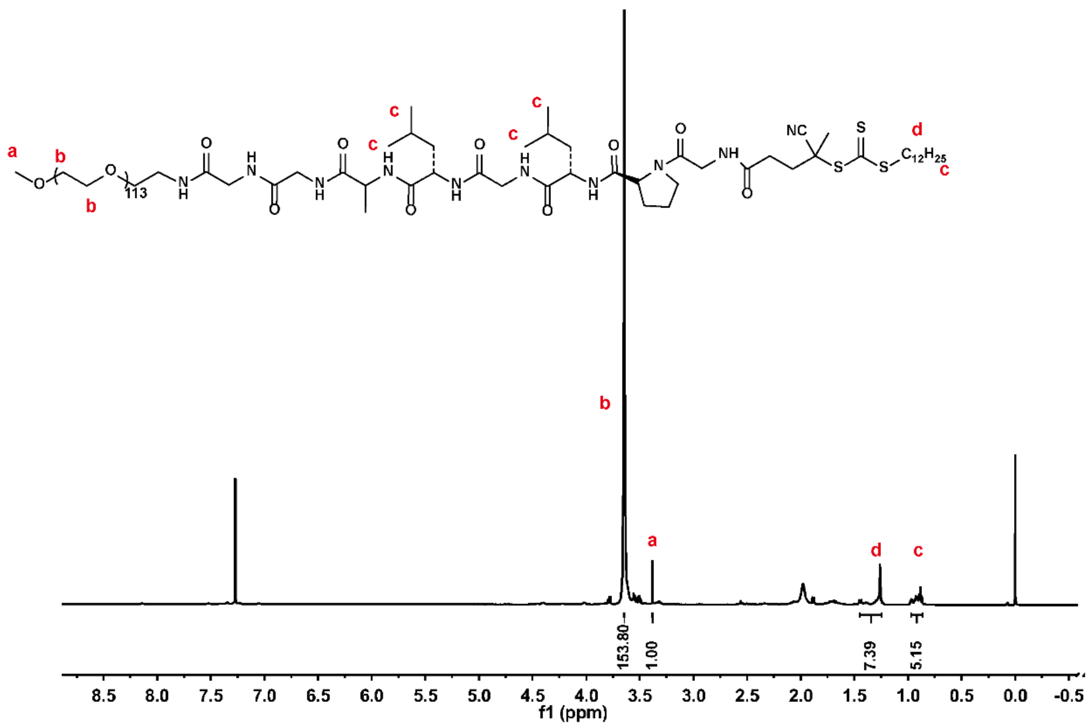

Supplementary Figure 20. ^{1}H-NMR spectrum of compound **26** ($CDCl_3$).

compound **27**

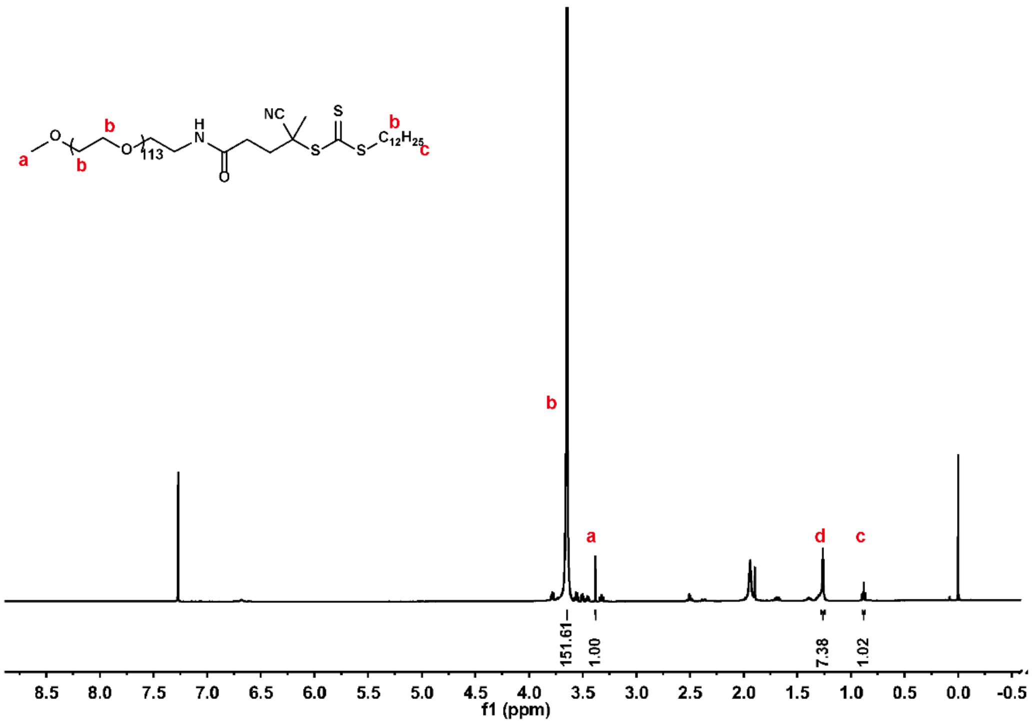

Supplementary Figure 21. ^{1}H-NMR spectrum of compound **27** ($CDCl_3$).

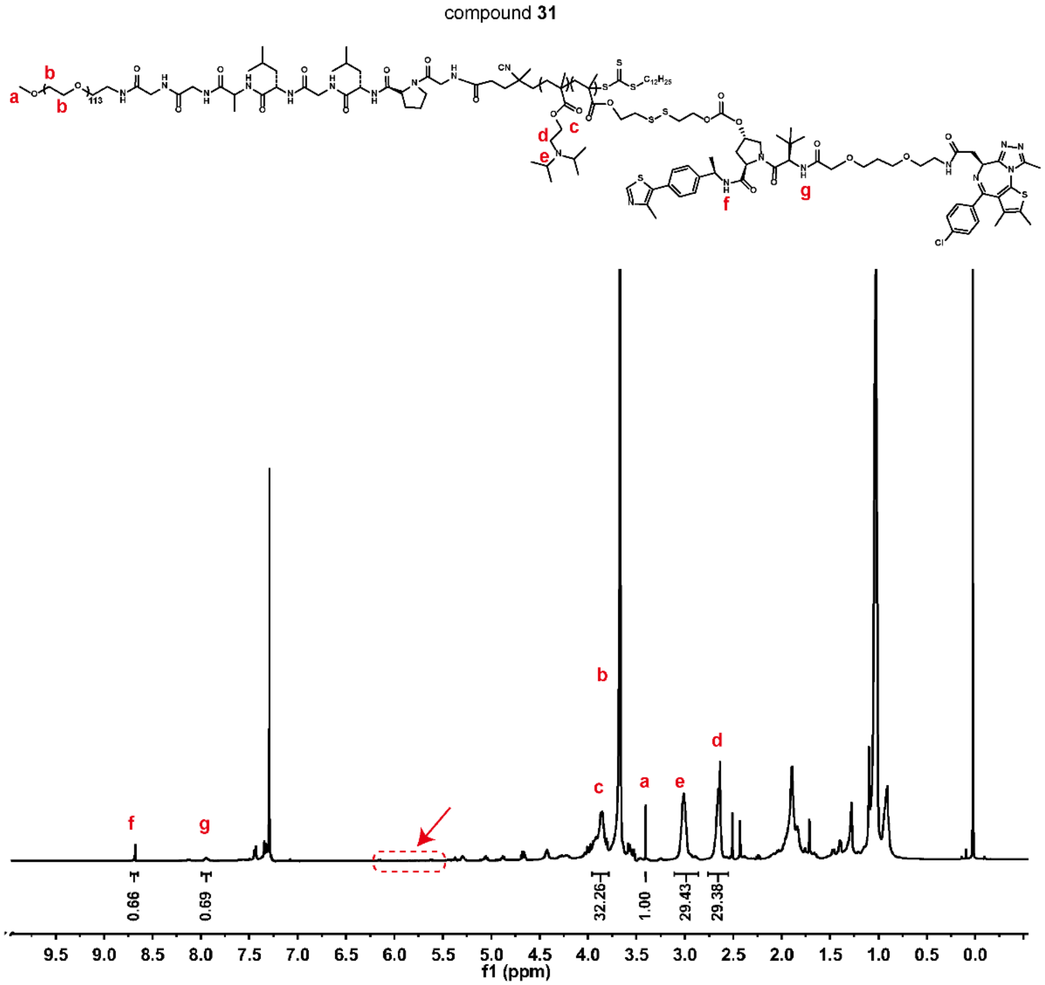

Supplementary Figure 22. ^{1}H-NMR spectrum of compound **31** ($CDCl_3$).

compound **32**

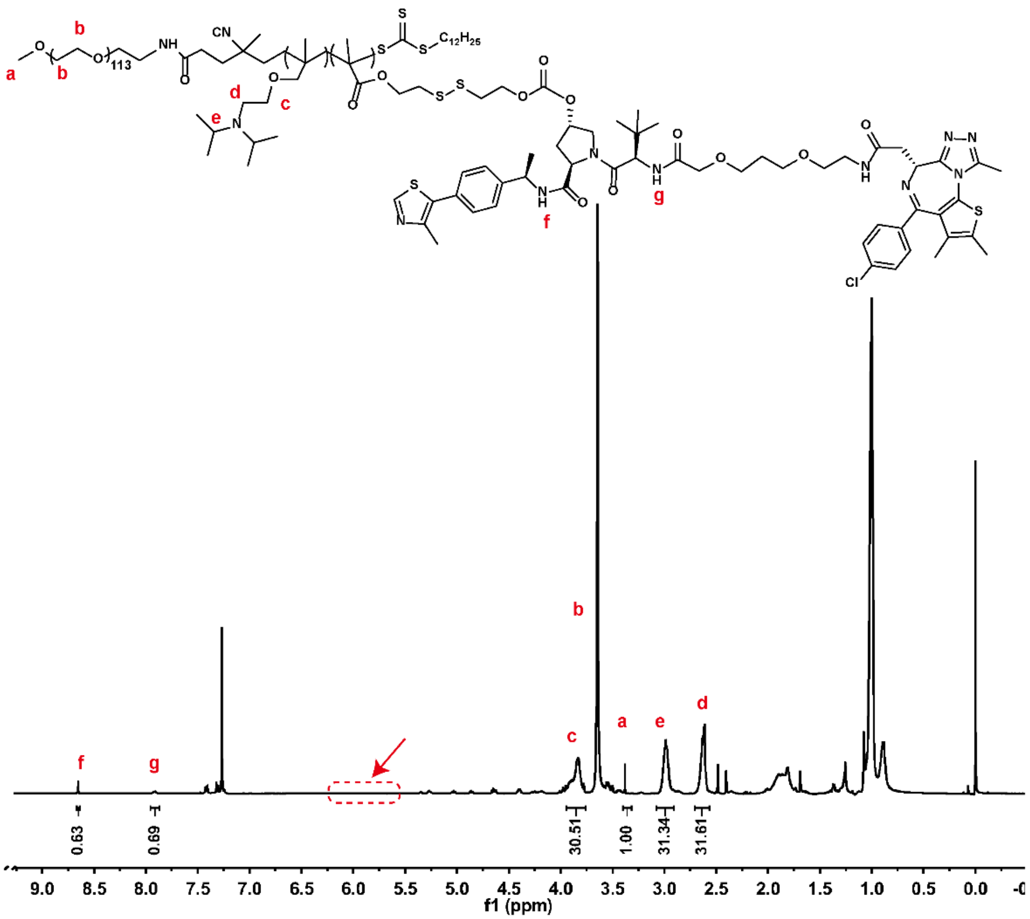

Supplementary Figure 23. ^{1}H-NMR spectrum of compound **32** ($CDCl_3$).

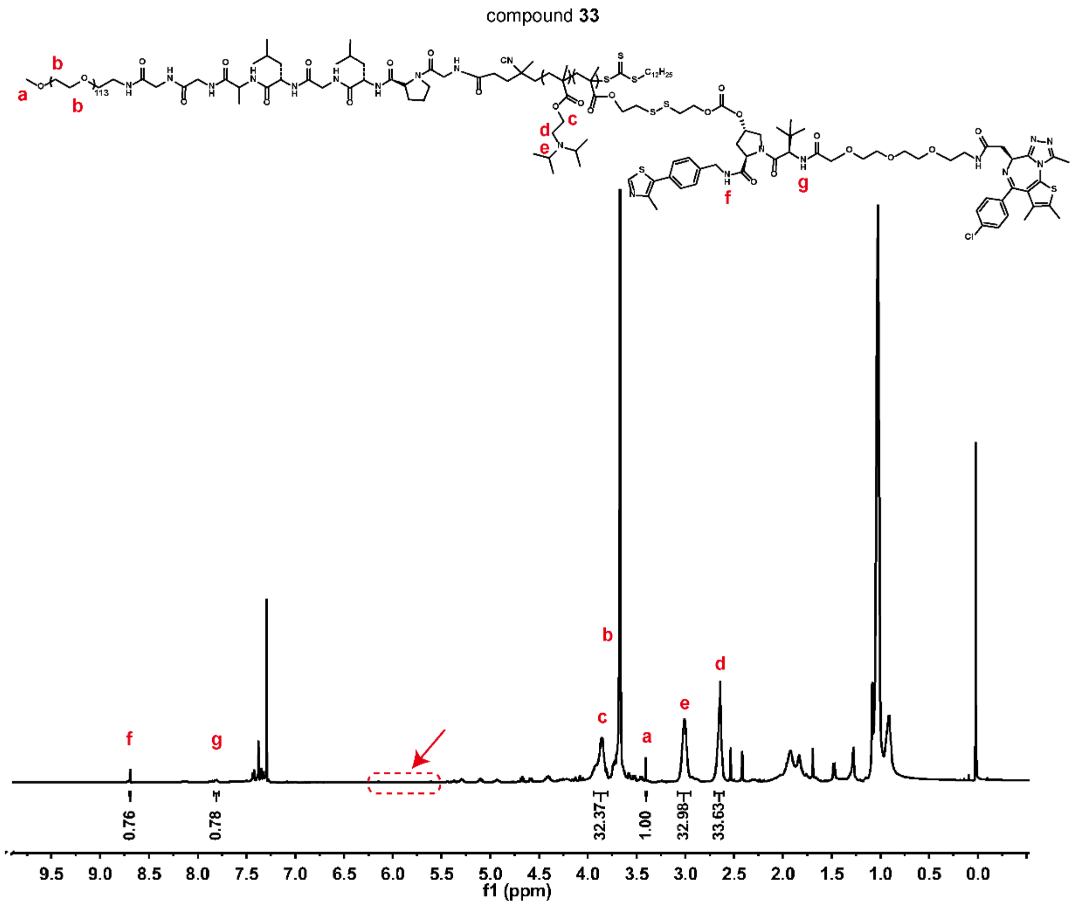

Supplementary Figure 24. ^{1}H-NMR spectrum of compound **33** ($CDCl_3$).

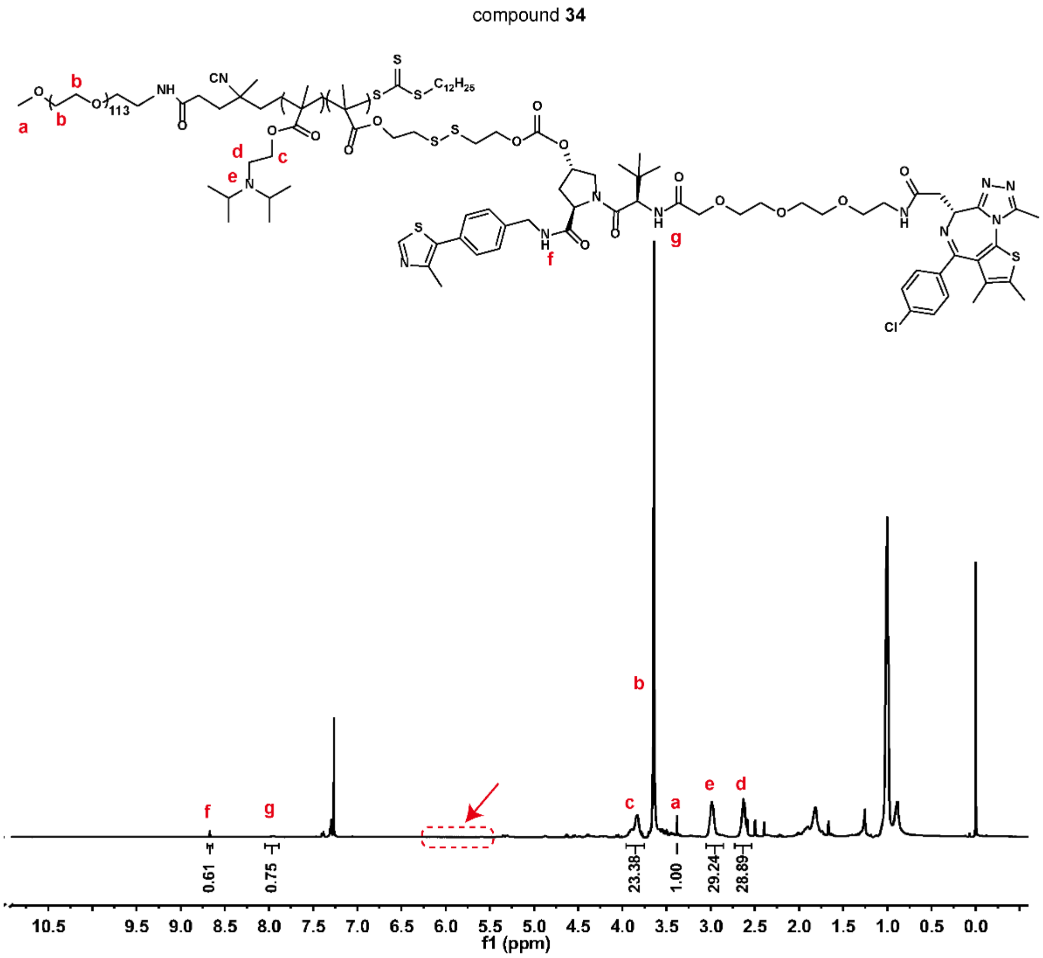

Supplementary Figure 25. ^{1}H-NMR spectrum of compound **34** ($CDCl_3$).

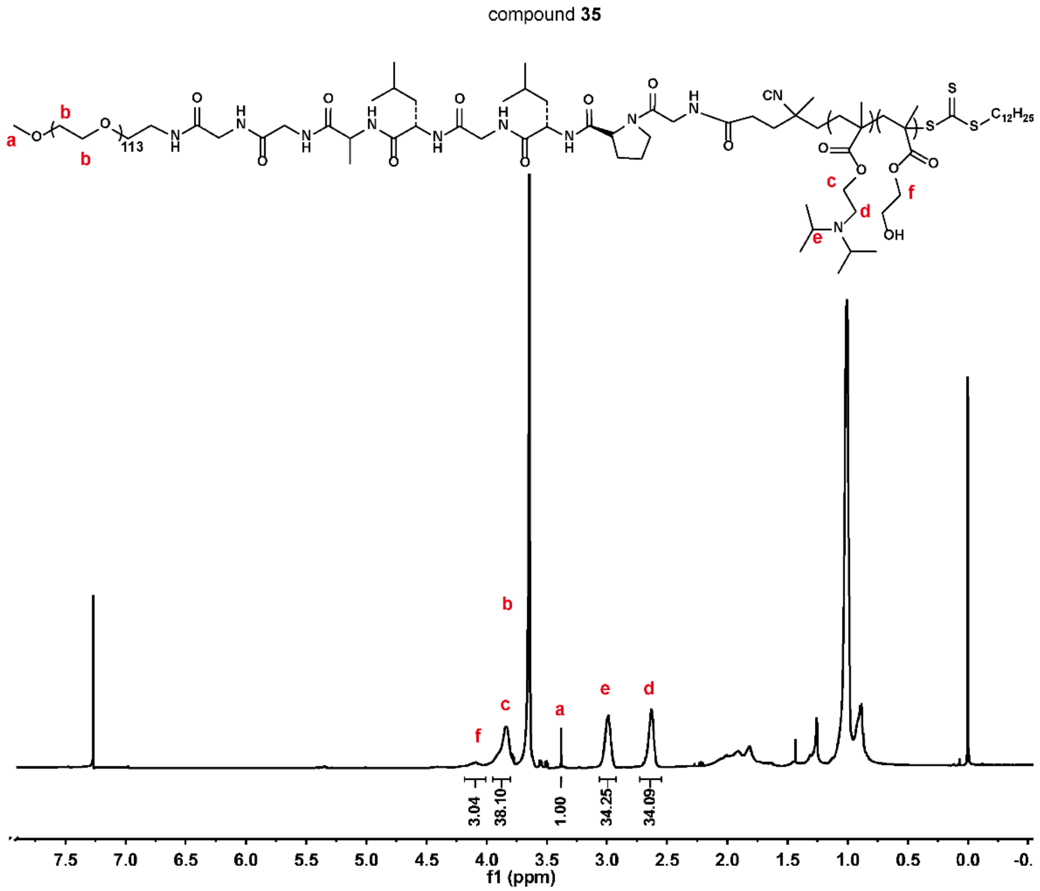

Supplementary Figure 26. The ^{1}H-NMR spectrum of compound **35** ($CDCl_3$).

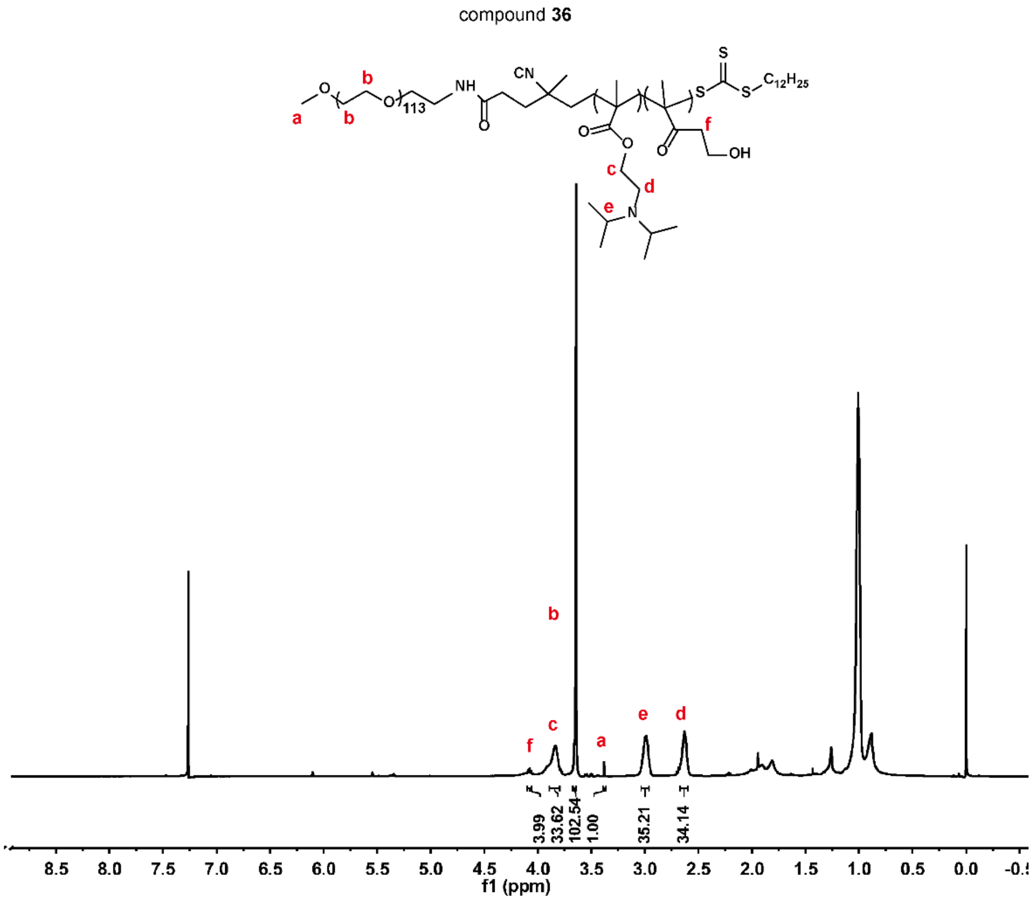

Supplementary Figure 27. ^{1}H-NMR spectrum of compound **36** ($CDCl_3$).

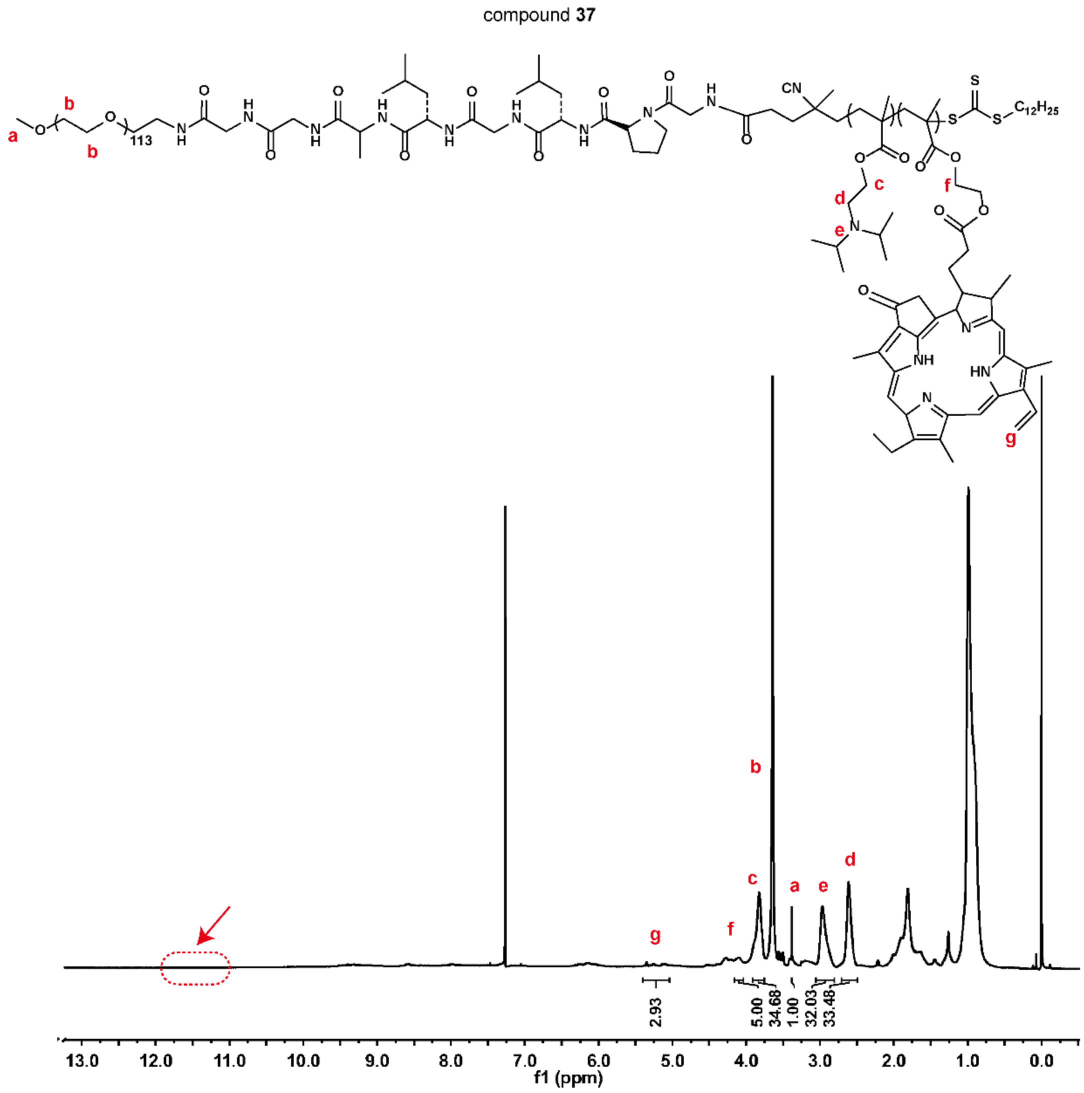

Supplementary Figure 28. ^{1}H-NMR spectrum of compound **37** ($CDCl_3$).

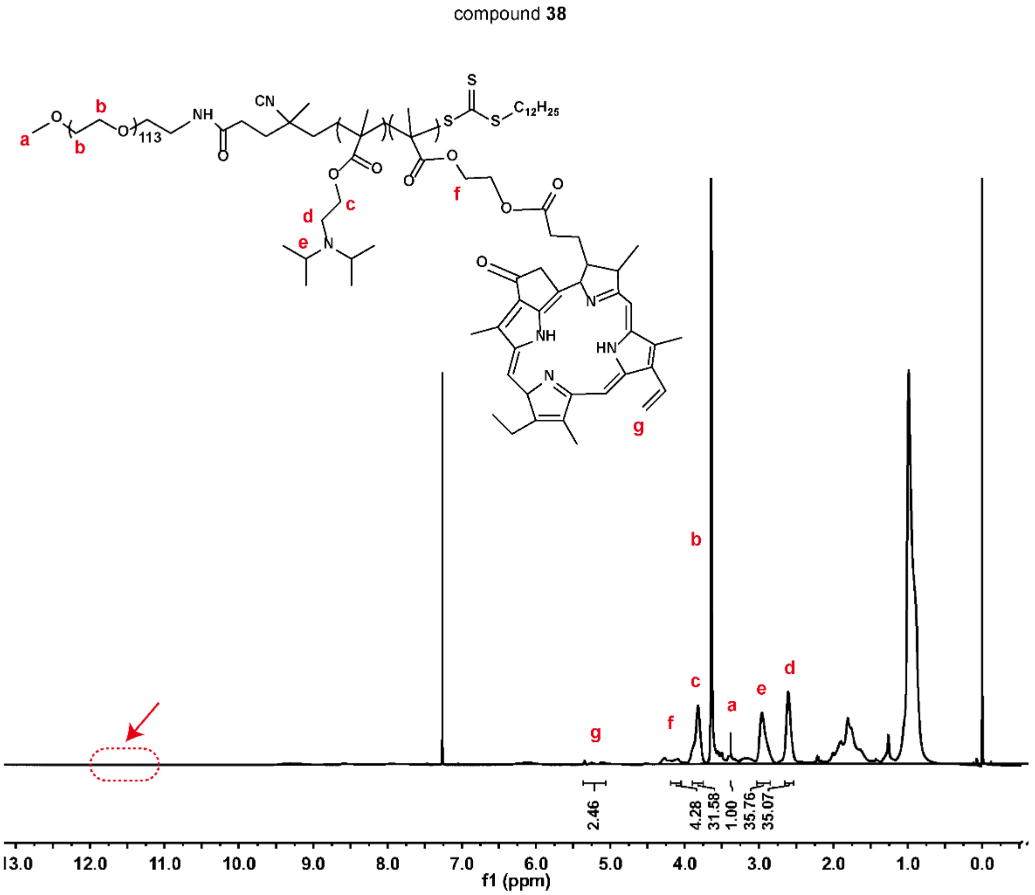

Supplementary Figure 29. ^{1}H-NMR spectrum of compound **38** ($CDCl_3$).

compound **39**

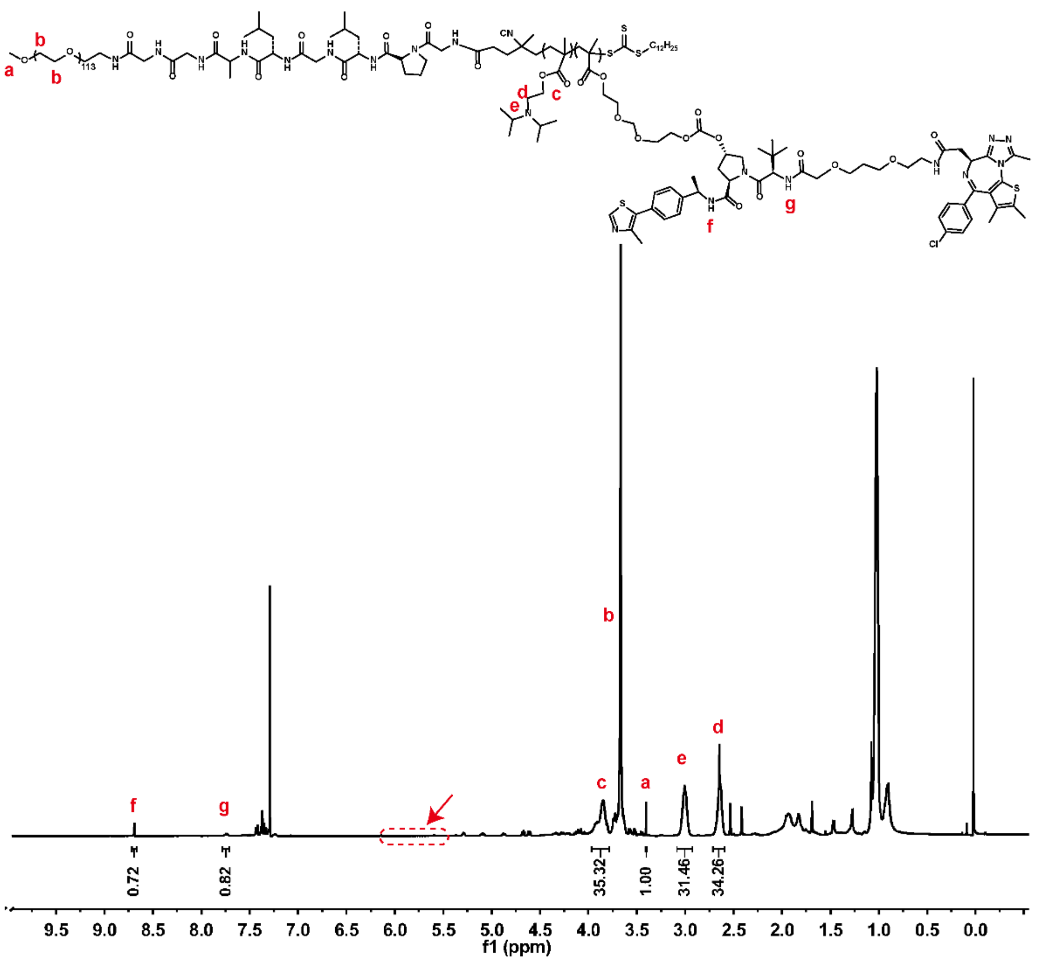

Supplementary Figure 30. ^{1}H-NMR spectrum of compound **39** ($CDCl_3$).

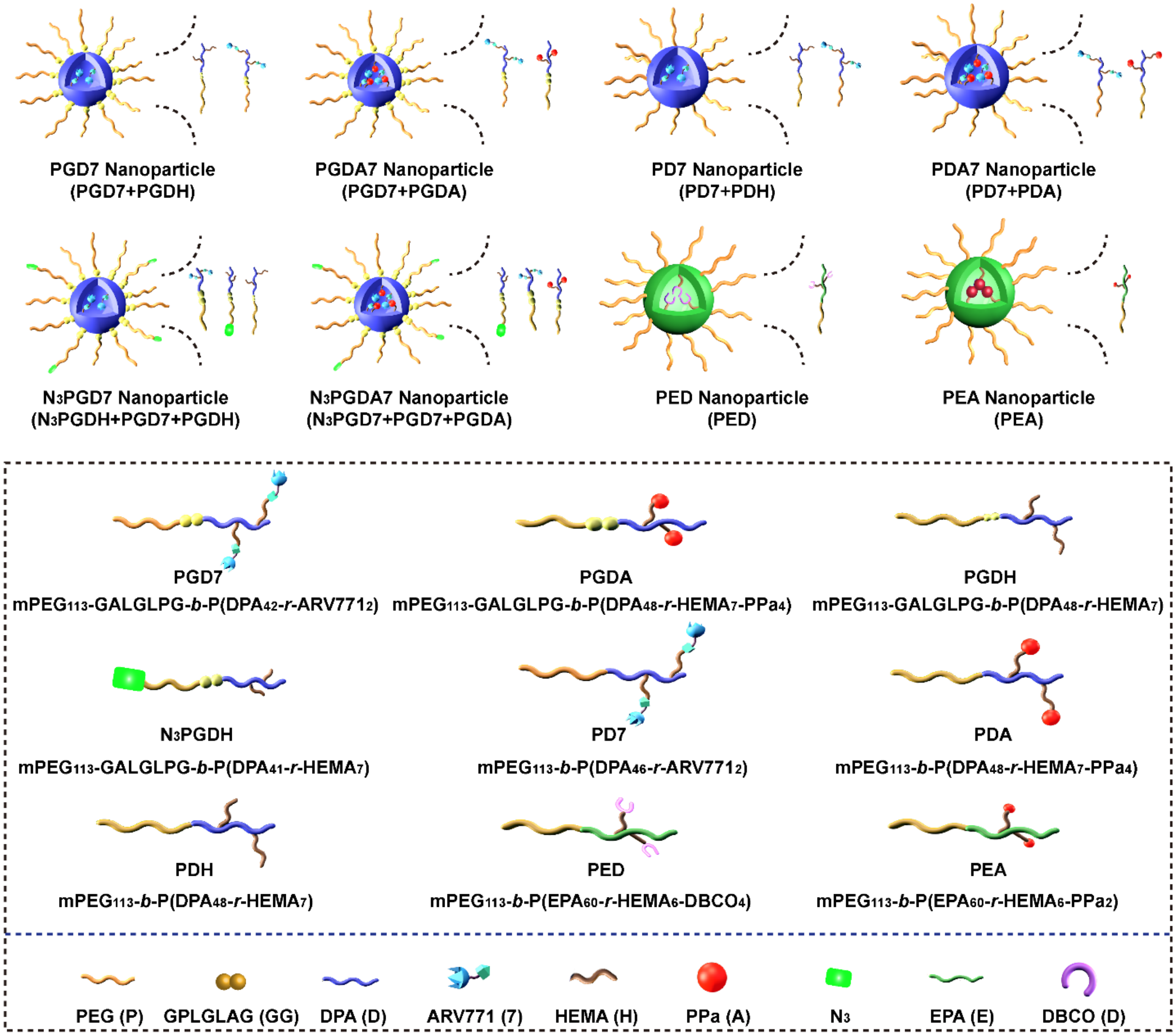

Supplementary Figure 31. Carton illustration of nanoparticle compositions and acronyms investigated throughout the study.

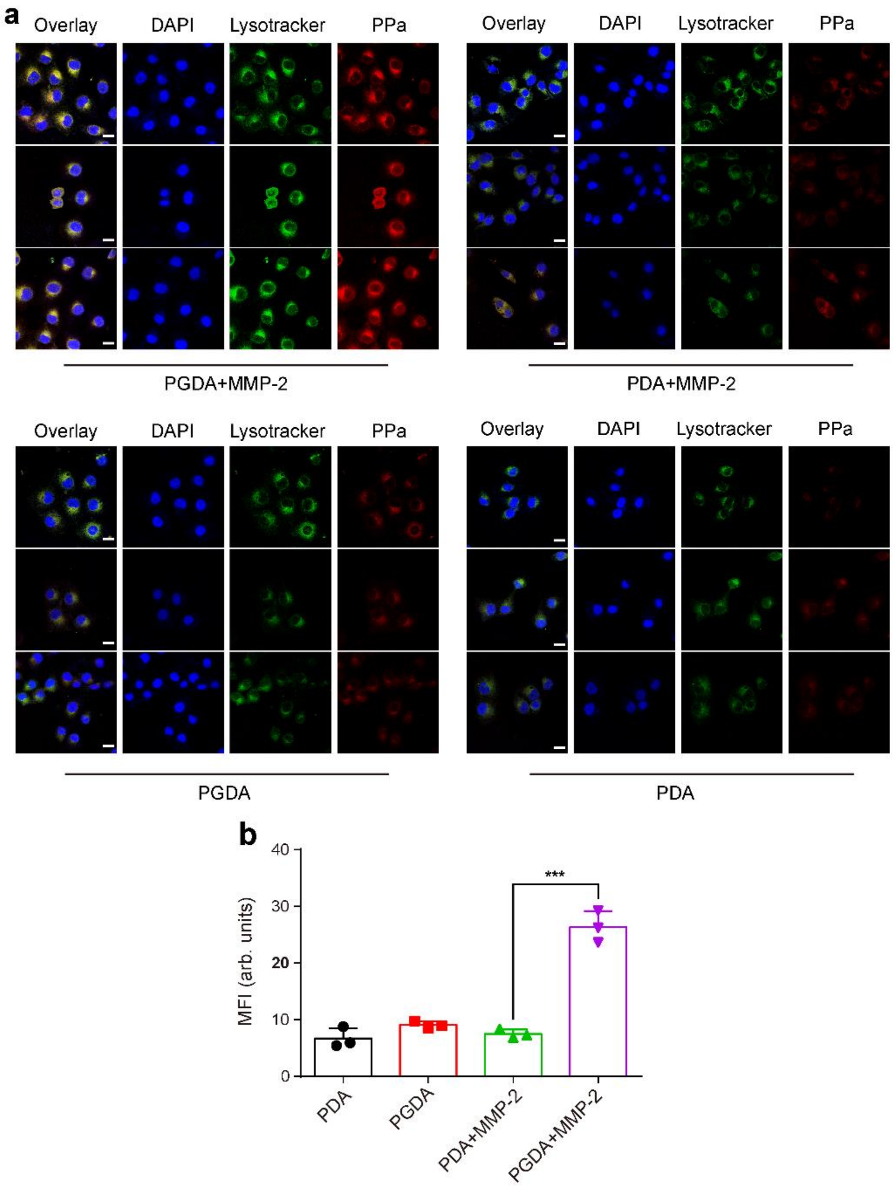

Supplementary Figure 32. a. CLSM examination of intracellular distribution of the PPa-labeled POLY-PROTAC nanoparticles in MDA-MB-231 tumor cells in vitro, and **b**. Integrated intracellular fluorescence intensity of POLY-PROTAC nanoparticles-treated MDA-MB-231 tumor cells upon 12 h incubation (scale bar = 20 μm, n = 3 biologically independent cells, statistical analysis was performed by two-sided unpaired t-test). Data are presented as mean ± SD.

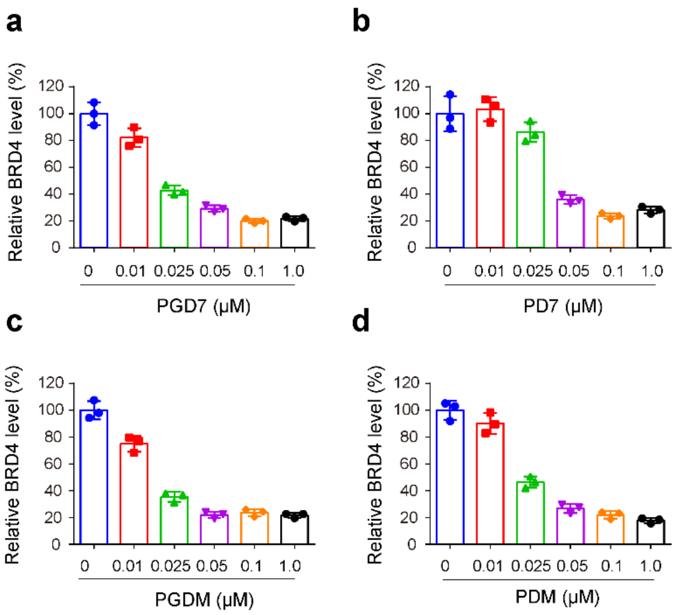

Supplementary Figure 33. Normalization of **a.** PGD7, **b**. PD7, **c**. PGDM, and **d**. PDM NP-mediated BRD4 degradation in MDA-MB-231 tumor cells in vitro as a function of ARV771 concentration. The cells were incubated with the NPs for 24 h and then examined by western blot assay. All data are presented as mean ± SD. (n = 3 biologically independent cells)

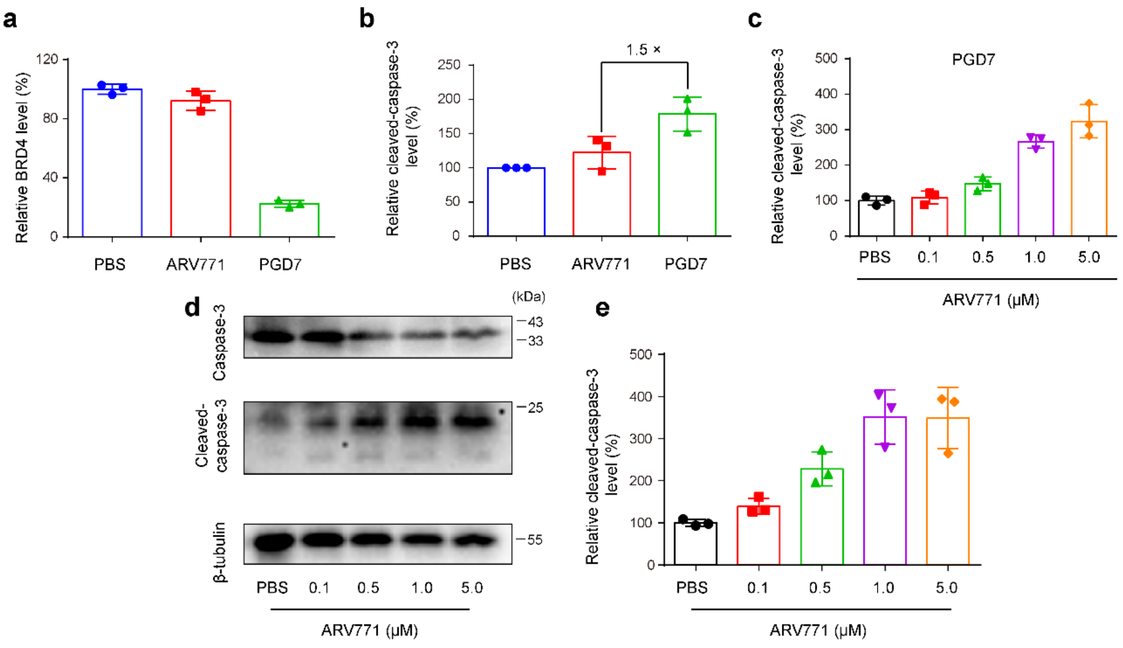

Supplementary Figure 34. a. Semi-quantitation of western blot band showed in Fig. 4h for BRD4 expression in MDA-MB-231 tumor examined at the end of the anti-tumor study in vivo (n = 3 biologically independent mice); **b.** Semi-quantitation of western blot band showed in Fig. 4h for caspase-3 activation in the MDA-MB-231 tumor in vivo upon ARV771 or POLY-PROTAC NPs treatment (the tumors were harvested at the second day post five-cycles treatments, n = 3 biologically independent mice); **c**. Semi-quantitation of the western blot band in Fig. 4i for PGD7 NP-induced caspase-3 activation in MDA-MB-231 tumor cells in vitro (n = 3 biologically independent cells); **d.** Western-blot assay, and **e**. Semi-

quantitation of the western blot band of ARV771-induced caspase-3 activation in MDA-MB-231 cells in vitro. The cells were treated with ARV771 for 24 h and then examined by western blot assay (n = 3 biologically independent cells). All data are presented as mean ± SD.

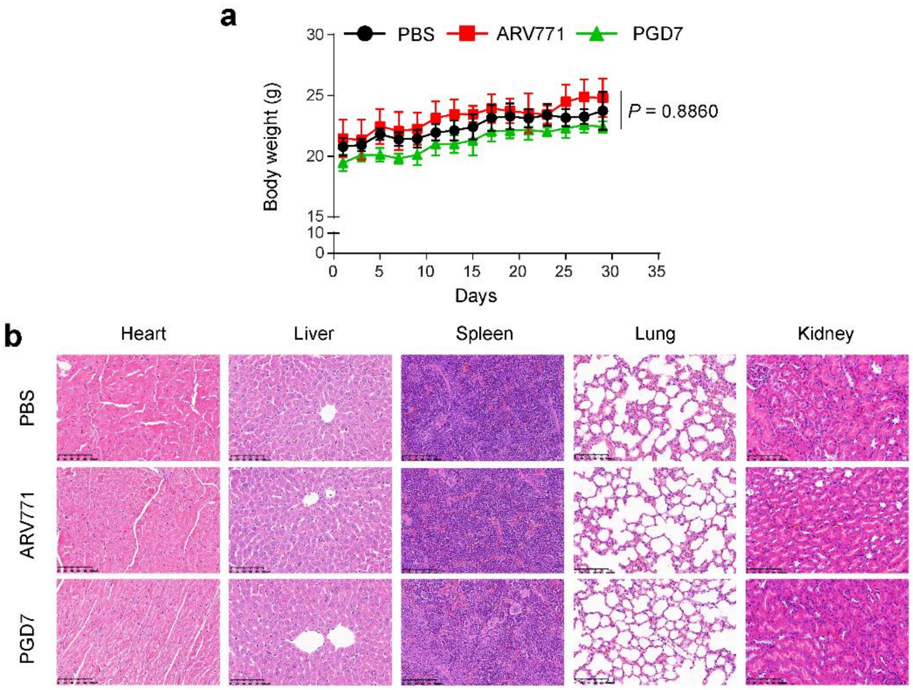

Supplementary Figure 35. a. Body weight change of the tumor-bearing Balb/c nude mice during the experimental period (n = 5 biologically independent mice, statistical analysis was performed by one-way ANOVA with a Brown-Forsythe test); **b.** H&E staining of the major organs at the end of anti-tumor study (heart, liver, spleen, lung, kidney). All data are presented as mean ± SD.

compound **40**

a

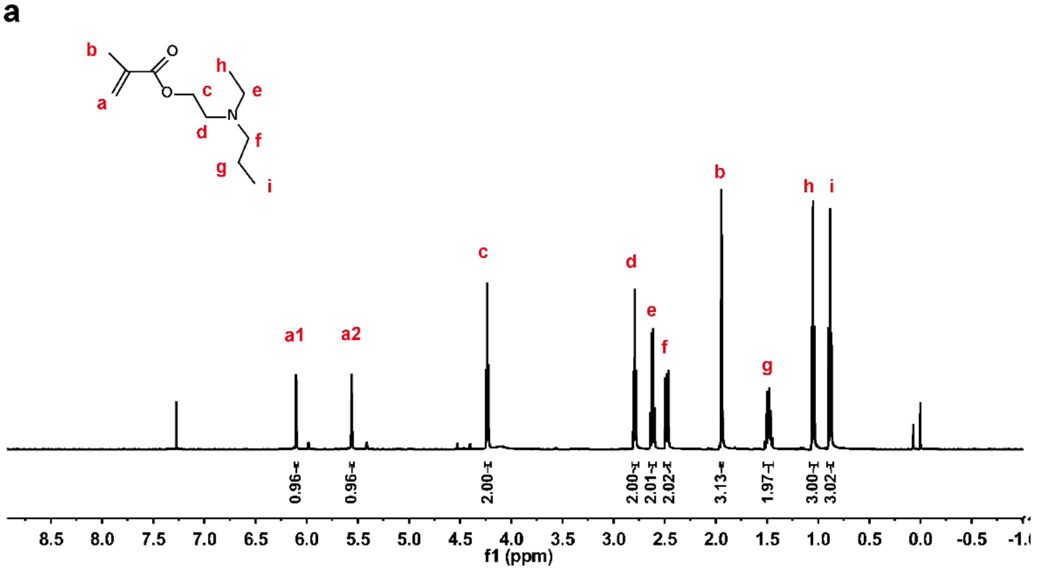

b

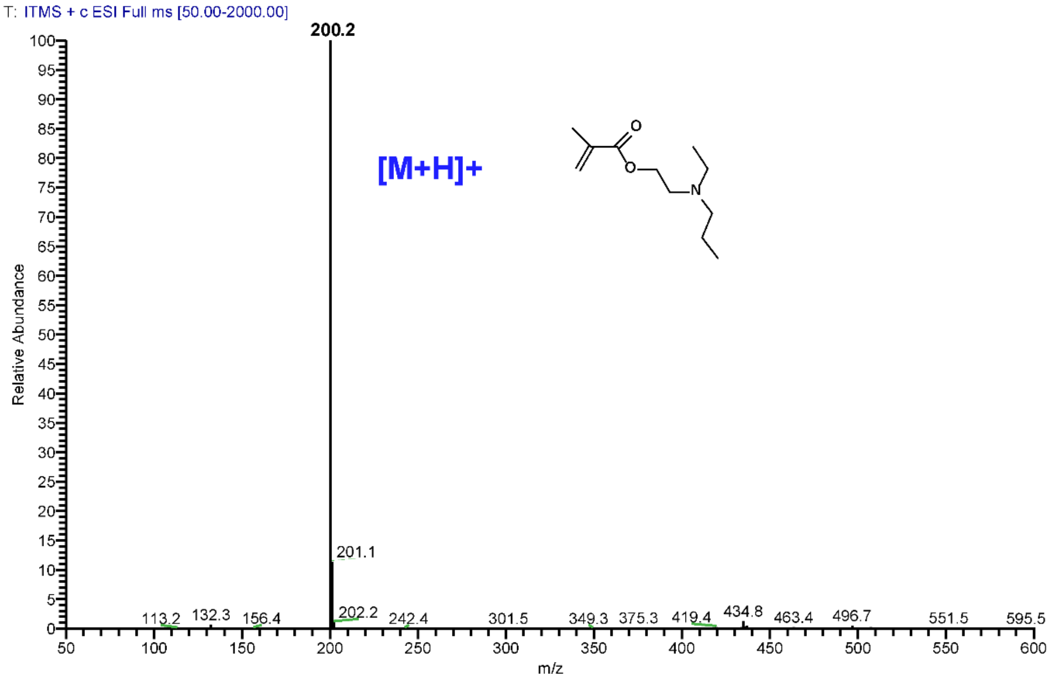

Supplementary Figure 36. a. ^{1}H-NMR spectrum ($CDCl_3$), and **b**. mass spectrum of compound **40**.

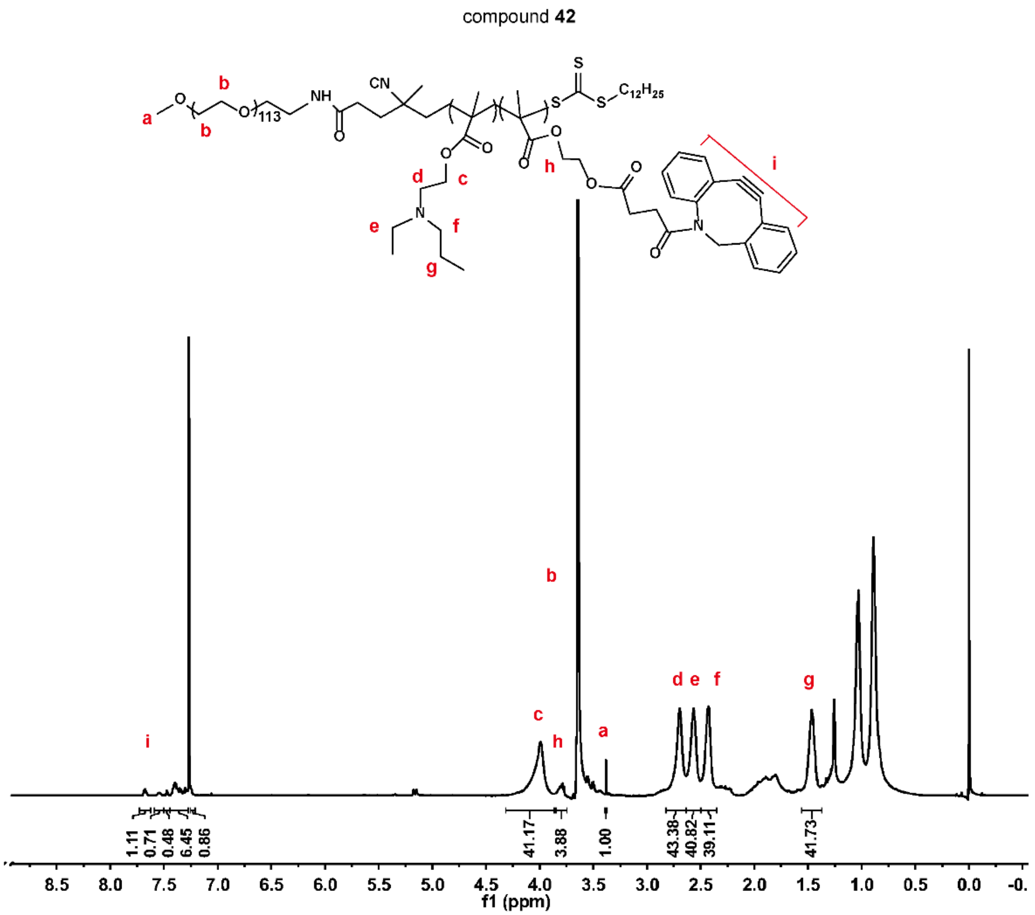

Supplementary Figure 37. ^{1}H-NMR spectrum of compound **42** ($CDCl_3$).

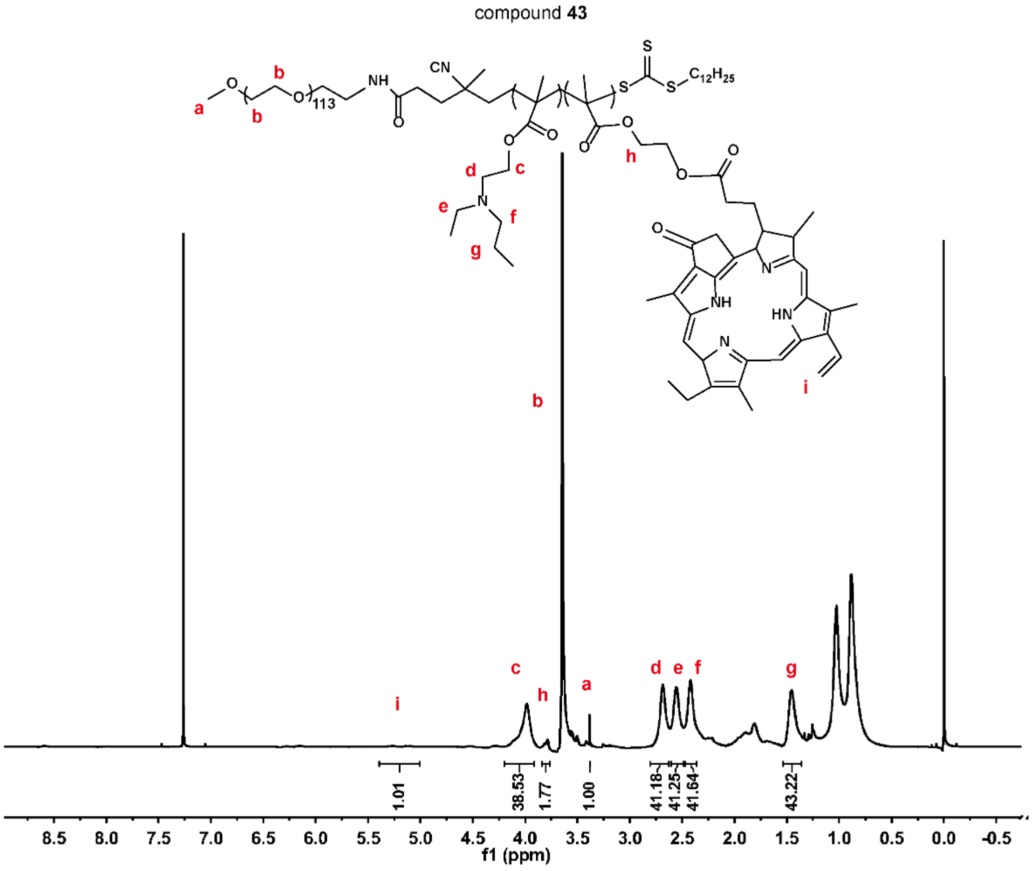

Supplementary Figure 38. ^{1}H-NMR spectrum of compound **43** ($CDCl_3$).

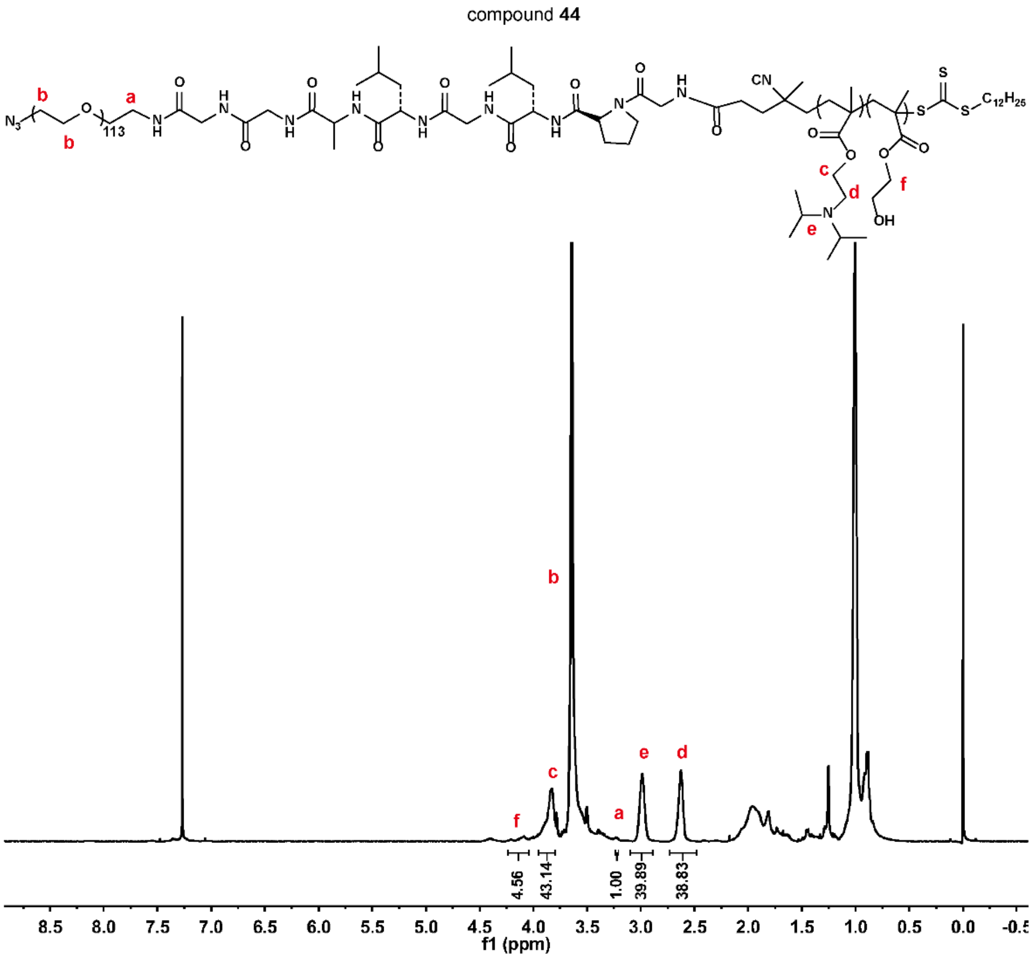

Supplementary Figure 39. ^{1}H-NMR spectrum of compound **44** ($CDCl_3$).

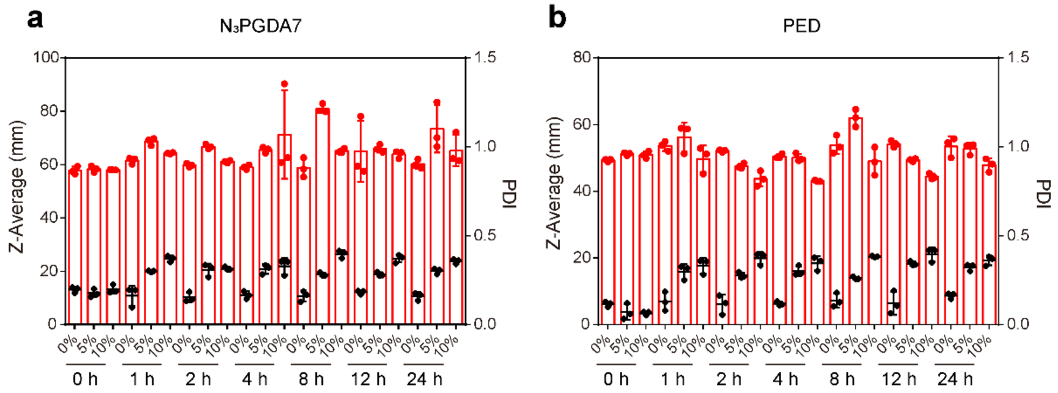

Supplementary Figure 40. The serum stability of the POLY-PROTAC nanoparticles. Averaged hydrodynamic diameter and PDI of **a**. N_3@PGDA7, and **b**. PED nanoparticle as a function of fetal bovine serum concentrations and incubation time (n = 3 independent experiments). All data are presented as mean ± SD.

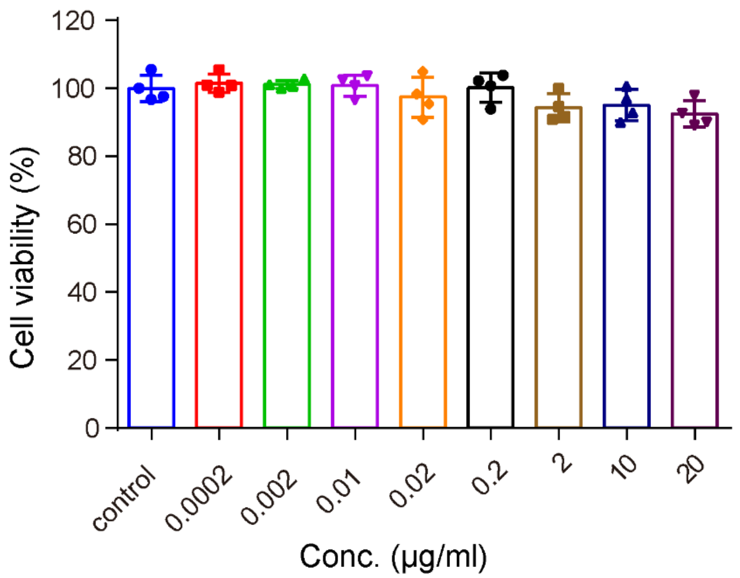

Supplementary Figure 41. Cytotoxicity assay of ARV771-free PGD7 NPs in MDA-MB-231 tumor cells in vitro. The cells were incubated with the NPs for 72 h, and examined by CCK-8 assay (n = 4 biologically independent cells). All data are presented as mean ± SD.

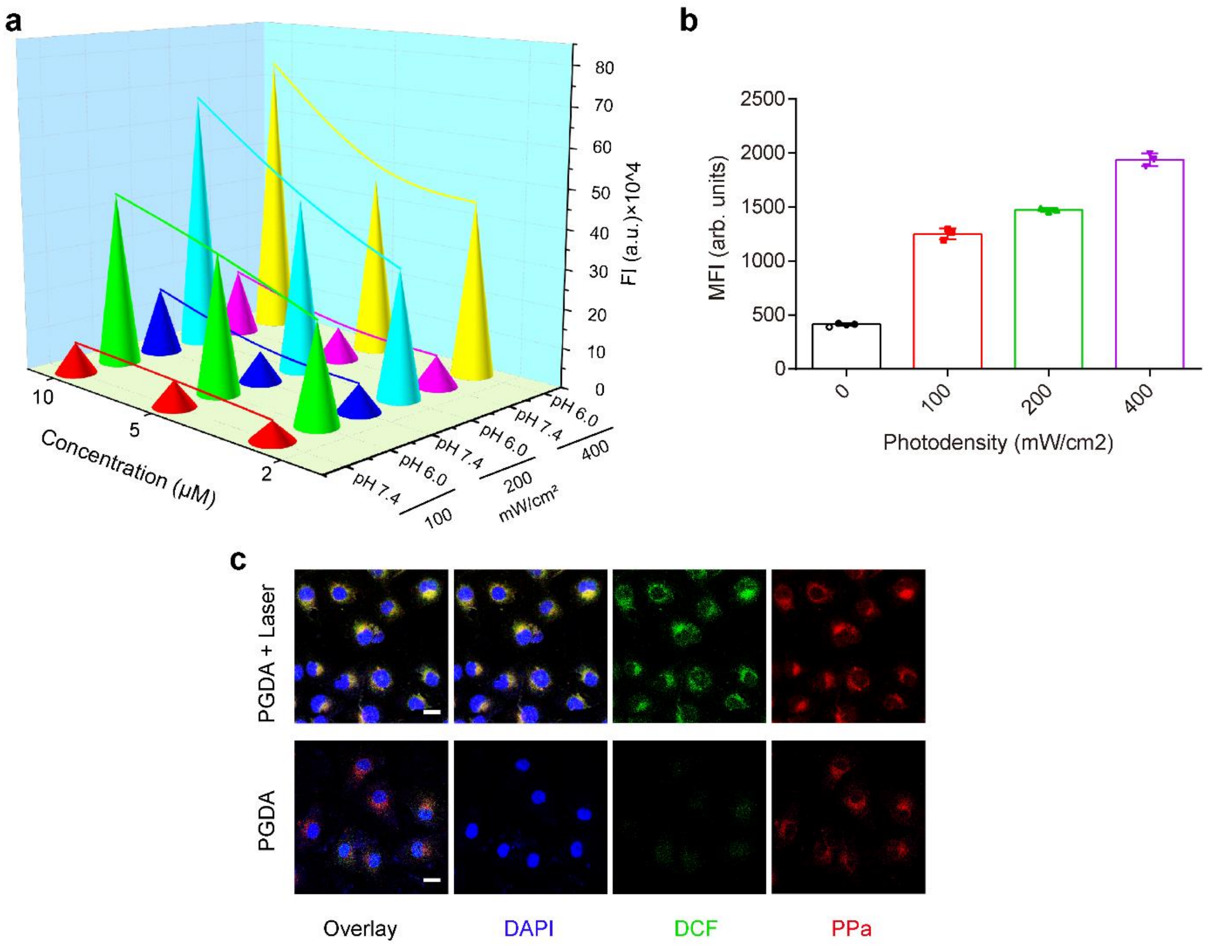

Supplementary Figure 42. PDT activity of the PGDA NPs in MBA-MB-231 tumor cells in vitro. **a.** Fluorescence intensity of SOSG, a ROS probe, which incubated with PGDA nanoparticle at different PPa

concentration in pH 7.4 or pH 6.0 and then irradiated with 671 nm laser at various photodensity; **b.** Flow cytometry examination of intracellular fluorescence signal of DCF. The MDA-MB-231 cells were incubated with PGDA nanoparticles at identical PPa concentration for 12 h, and then irradiated with 671 nm laser at pre-determined photodensity (n = 3 biologically independent cells); **c.** CLSM examination of PDT-induced ROS generation in MDA-MB-231 cells in vitro. The cells were pretreated with PGDA NPs for 12 h, and then irradiated with 671 nm laser (photodensity: 200 mW/cm^2, scale bar = 20 μm). All data are presented as mean ± SD.

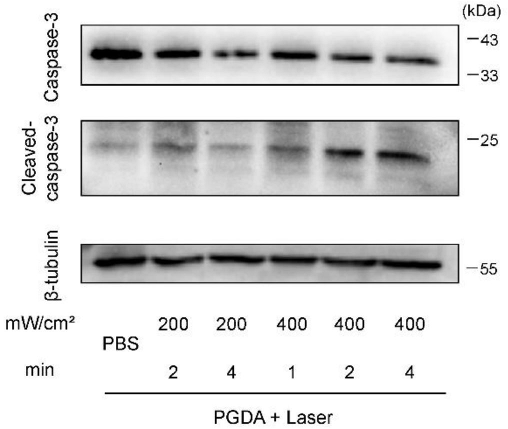

Supplementary Figure 43. Western blot assay of PDT-induced caspase-3 activation in the MDA-MB-231 cells in vitro. MDA-MB-231 tumor cells were treated with PGDA nanoparticle at a PPa concentration of 2.5 μM, and then were irradiated by 671 nm laser with different photodensity and irradiation time. The cells were collected for western blot assay after 24 h incubation.

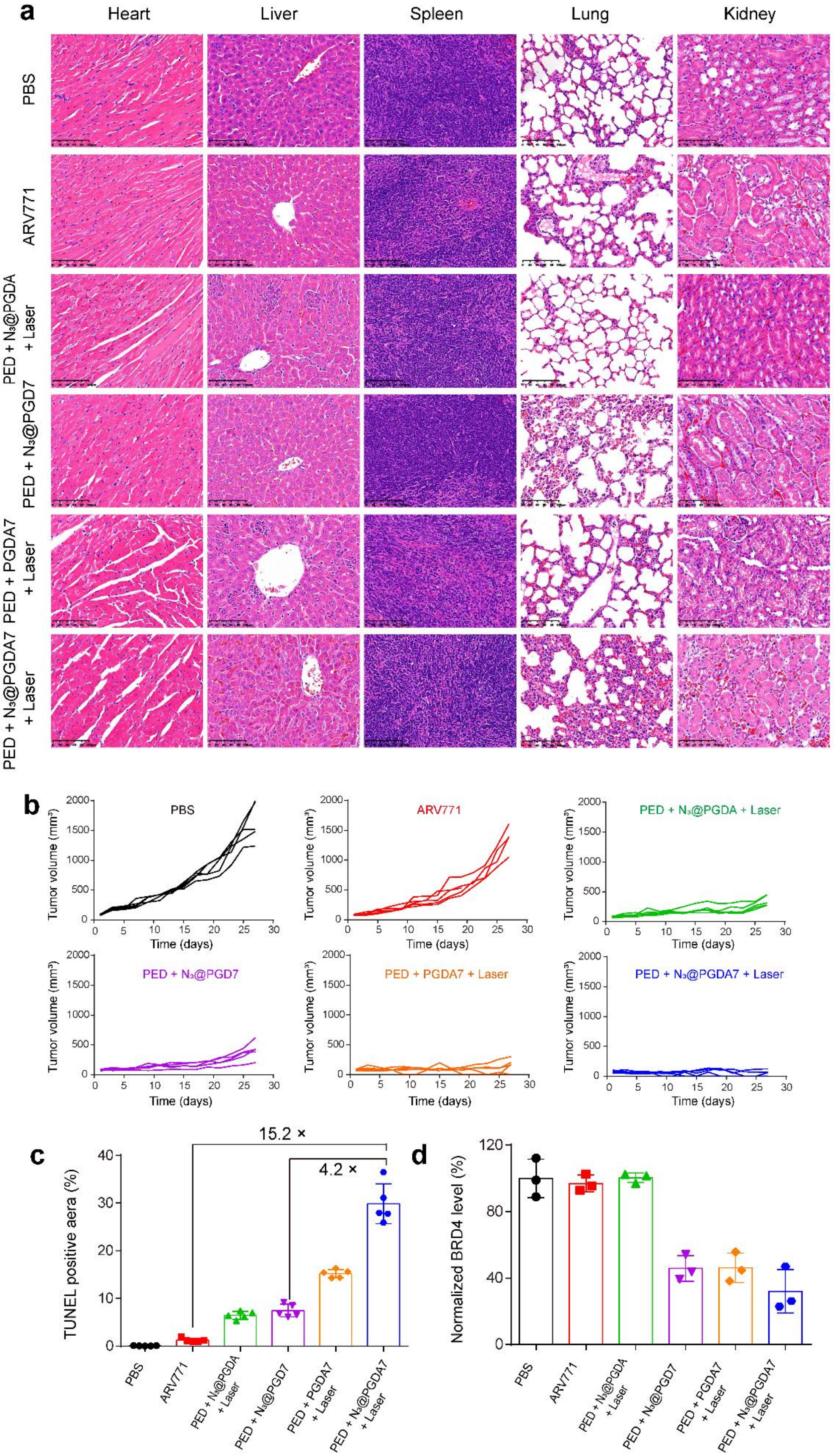

Supplementary Figure 44. Anti-tumor performance of the bioorthogonal POLY-PROTAC nanoparticle via BRD4 degradation and PDT. **a.** H&E staining of the major organs (heart, liver, spleen, lung, kidney)

of MDA-MB-231 tumor-bearing mice (scale bar = 100 μm); **b.** The individual tumor growth curves after treating with various formulations (PBS, ARV771, PED + N_3@PGDA + Laser, PED + N_3@PGD7, PED + PGD7 + Laser, PED + N_3@PGDA7 + Laser (n = 5 biologically independent mice, photodensity: 400 mW/cm^2, 5 min); **c.** Semi-quantitative analysis of TUNEL staining of the tumor sections (n = 5 biologically independent mice); **d.** Semi-quantitation of western-blot-determined BRD4 expression in the tumor tissues post-treatments (n = 3 biologically independent mice). All data are presented as mean ± SD.

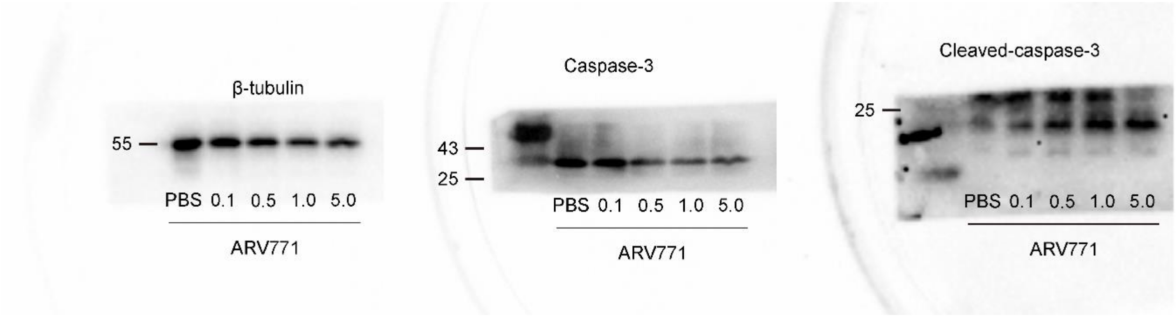

Supplementary Figure 45. Uncropped western blot source data for Supplementary Figure 34d.

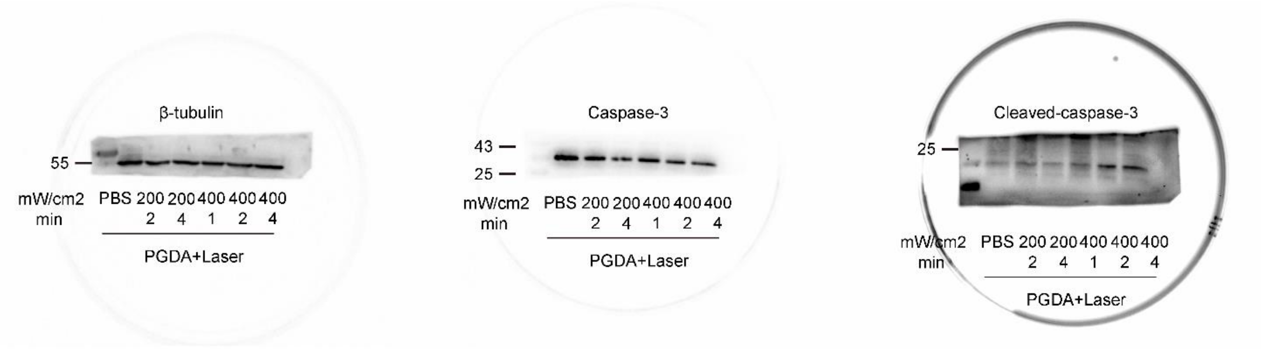

Supplementary Figure 46. Uncropped western blot source data for supplementary Figure 43.

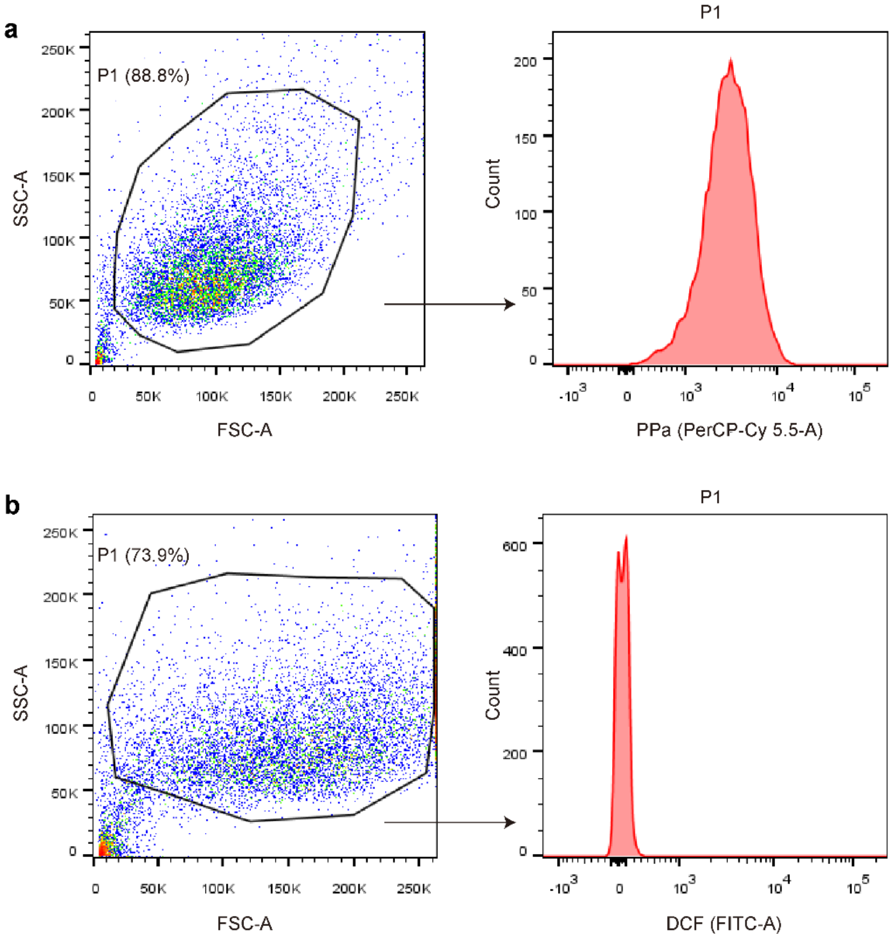

Supplementary Figure 47. FACS gating strategies for (a) cellular uptake (related to Figure 3b) and (b) ROS generation (related to Supplementary Figure 42b).
